# The Dutch cardiomyopathy registry (DCR); rationale and registry design

**DOI:** 10.1007/s12471-026-02063-5

**Published:** 2026-08-10

**Authors:** Peter-Paul Zwetsloot, Michiel T. H. M. Henkens, Erik P. A. van Iperen, Michiel Minten, Evelien van der Schaaf-de Wolf, Linda W. van Laake, Anneline S. J. M. te Riele, Pim van der Harst, Jessica van Setten, Vanessa P. M. van Empel, Hans-Peter Brunner-La Rocca, D. Job A. J. Verdonschot, Moniek G. P. J. Cox, Imke Christiaans, Nina Ajmone Marsan, Karin van Spaendonck-Zwarts, Daniela QCM Barge-Schaapveld, Etienne Cramer, Yvonne M. Hoedemakers, Michelle Michels, Judith M. A. Verhagen, Henk S. Schipper, Malou van den Boogaard, Folkert W. Asselbergs, Dennis van Veghel, Katja Zeppenfeld, Robin Nijveldt, Peter van Tintelen, Peter van der Meer, J. Wouter Jukema, Jolanda van der Velden, Connie R. Bezzina, Rudolf A. de Boer, Stephane R. B. Heymans

**Affiliations:** 1 Netherlands Heart Institute, Utrecht, The Netherlands; 2 Thoraxcenter, Department of Cardiology, Erasmus Medical Center, Cardiovascular Institute, Rotterdam, The Netherlands; 3 European Reference Network for rare, low prevalence and complex diseases of the heart: ERN GUARD-Heart, ERN GUARDHEART, Rotterdam, The Netherlands; 4 Department of Pathology, Maastricht University Medical Center (MUMC+), Maastricht, The Netherlands; 5 MEMIC, Center for data and information management Maastricht University, Maastricht, The Netherlands; 6 Department of Cardiology, University Medical Center Utrecht and Utrecht University, Utrecht, The Netherlands; 7 Department of Cardiology, Maastricht University Medical Center, Cardiovascular Research Institute Maastricht (CARIM), Maastricht, The Netherlands; 8 Department of Clinical Genetics, Maastricht University, Cardiovascular Research Institute Maastricht (CARIM), Maastricht, The Netherlands; 9 Department of Cardiology, University Medical Center Groningen, Groningen, The Netherlands; 10 Department of Genetics, University Medical Center Groningen, University of Groningen, Groningen, The Netherlands; 11 Department of Cardiology, Leiden University Medical Center, Leiden, The Netherlands; 12 Department of Clinical Genetics, Leiden University Medical Center, Leiden, The Netherlands; 13 Department of Cardiology, Radboud University Medical Center, Nijmegen, The Netherlands; 14 Department of Clinical Genetics, Radboud University Medical Center, Nijmegen, The Netherlands; 15 Department of Clinical Genetics, Erasmus MC, University Medical Center Rotterdam, Rotterdam, The Netherlands; 16 Department of Pediatric Cardiology, Erasmus MC: Sophia’s Children Hospital, Rotterdam, The Netherlands; 17 Department of Cardiology, Amsterdam University Medical Center, Amsterdam, The Netherlands; 18 Department of Experimental Cardiology, Amsterdam University Medical Center, Amsterdam, The Netherlands; 19 Netherlands Heart Registration, Utrecht, The Netherlands; 20 Department of Clinical Genetics, University Medical Center Utrecht, Utrecht, The Netherlands; 21 Department of Physiology, Amsterdam Cardiovascular Sciences, Amsterdam UMC, Amsterdam, The Netherlands

**Keywords:** Cardiomyopathy, Hypertrophic, Cardiomyopathy, Dilated, Cardiomyopathies, Genetics, Registries

## Abstract

**Background:**

Cardiomyopathies are important causes of arrhythmias, sudden cardiac death, and heart failure. With accumulating knowledge in clinical, genetic, and molecular phenotyping, the number of distinct disease entities is growing. There is a need for broad, inclusive, high-quality prospective multi-center registries to better analyze these phenotypes, provide accurate risk stratification, and tailor therapies. The Dutch Cardiomyopathy Registry (DCR) will serve as a longitudinal registry for all cardiomyopathy patients, exploiting previously built local databases, allowing adequately powered research studies.

**Methods:**

The DCR is a multi-center observational registry that unites all Dutch University Medical centers. The registry will have local and central shared databases, which will be hosted and coordinated by the Netherlands Heart Institute. Patients with an established cardiomyopathy diagnosis and carriers of pathogenic or likely-pathogenic cardiomyopathy gene variants will be included. Furthermore, selected family members and individuals who are referred for screening for potential cardiomyopathy are eligible for inclusion. The subjects will receive, per best clinical practice, guideline-recommended diagnostics and treatment. Clinical data will be collected from clinical care at baseline and follow-up, including clinical visits, imaging studies, laboratory assessments, ECG, and additional functional tests. Outcome data consists of clinical performance status, cardiovascular complications and major adverse events, recorded through regular electronic health record files.

**Conclusion:**

The DCR will function as a national database, providing relevant information on epidemiology, demographics, natural history, diagnosis, and treatment. This will aid risk prediction and monitoring of new therapies. The DCR can identify patients who are eligible for future studies, including registry-based research.

**Supplementary Information:**

The online version of this article (10.1007/s12471-026-02063-5) contains supplementary material, which is available to authorized users.

## Introduction

Cardiomyopathies are considered rare diseases, often hereditary, affecting young individuals. They are increasingly recognized and diagnosed as causes of arrhythmias, sudden cardiac death, and heart failure. Advances in genetic testing, insights into the underlying complex genetic architecture, phenotyping, and disease mechanisms have led to a dynamic disease landscape, with changing nomenclature and diagnostic algorithms, new therapies, and improved prognoses [1]. Apart from phenotypic descriptions, such as hypertrophic cardiomyopathy (HCM), dilated cardiomyopathy (DCM), non-dilated left ventricular cardiomyopathy (NDLVC), arrhythmogenic right ventricular cardiomyopathy (ARVC), restrictive cardiomyopathy (RCM), and infiltrative cardiomyopathy (ICM), the field is increasingly shifting towards combined genotype- and phenotype-specific characterization, which affords advantages for prognostication and targeted therapies. These include phenotype-specific therapies (e.g. myosin inhibitors for obstructive HCM), and other specific therapies, including gene therapy and next-generation targeted therapies [1]. Specifically, disease-specific or even gene/variant-specific risk models have been developed for the prediction of adverse outcomes. [2–4]. The absence of P/LP (pathogenic or likely-pathogenic) gene variants in well-defined phenotypes, the occurrence of P/LP variants in inflammatory cardiomyopathy phenotypes [5], the known association between common cardiac (metabolic) risk factors and cardiomyopathies [6, 7] and the observation that P/LP variants in the same gene can cause different cardiomyopathy forms [8, 9] are various reasons to promote broad cardiomyopathy registries across genotypes, phenotypes and gene-elusive patients. These complexities in genotype-phenotype relations [10] and other external factors necessitate retrospective and prospective collection of both phenotype- and gene-specific data.

The Dutch Cardiomyopathy Registry (DCR) is a new Dutch multi-center longitudinal registry that will potentially span all cardiomyopathy phenotypical expressions, building upon different local cardiomyopathy databases and protocols [11]. It aims to harmonize existing registries and will include all cardiomyopathy patients and relatives/family members, incorporating phenotyping, genetic testing, risk stratification, and guideline-recommended follow-up. It will facilitate research on all cardiomyopathy entities and serve as a validation cohort for other registries. Importantly, the DCR will start with the domains of HCM and DCM for practical reasons and hopes to welcome other entities in the future.

## Methods

The DCR is a multi-center prospective observational registry, coordinated by the Netherlands Heart Institute (NLHI). Its objectives are listed (Tab. 1). The DCR is structured around local databases that feed into a centralized national database, designed based on the Maastricht Cardiomyopathy registry platform [11]. A universal data dictionary was drafted in consultation with representatives from all centers, with participating centers approving the final first version.

**Table 1:** The objectives and informed consent harmonizations within the DCR.

Objectives
Unite and uniform local research registries, under similar harmonized informed consents in compliance with the latest data protection regulations (e.g. GDPR) and ethical standards, to enable lawful data sharing, reuse, and cross-site interoperability, while adhering to FAIR data principles (Findable, Accessible, Interoperable, Reusable) to maximize long-term scientific value and transparency
Provide longitudinal, high-quality data on existing cardiomyopathies on a national level
Collect data to inform diagnosis, risk stratification and therapies, while also improving insights into pathophysiology, prevention and socio-economic impact
Catalyse existing and new research projects through increased power and availability checks of patient data and biobanks
Serve as a dashboard and virtual waiting room for future studies on specific populations (specific gene variants, specific phenotypes)
Serve as a validation cohort for collaborative research
Share findings with the research, clinical and patient communities

### Ethics and informed consent

The registry integrates existing protocols, approved by Dutch ethical committees or waived by an institutional research ethics committee. Informed consent is obtained through these protocols, with consents being harmonized for the topics of data usage, potential biobanking, and data/sample sharing (Supplementary Tab. 1). Governance and databases are coordinated through NLHI, and a joint data registry agreement is signed at the initiation of the registry. The domains HCM/DCM are the disorders being installed first; additional cardiomyopathy domains will be potentially incorporated in due course.

The following registry protocols are united: The multi-center Cardiology Management and Prevention registry (previously known as Maastricht Cardiomyopathy Registry & Maastricht Biobank) (WMO NL765.85.068.21; METC21-017) [11]; Erasmus Medical Center Inherited Cardiomyopathy Registry and biobank [12]; Utrecht Cardiovascular Cohort—(UCC)-UNRAVEL [13]; Amsterdam University Medical Center (UMC) Biobank Cardiogenetica; Leiden UMC Biobank Cardiomyopathie; UMC Groningen GeneticLines and CardioLines Biobank

### Study population

We expect a total of 15,000 patients to be included. Eligible subjects are: Patients with a proven cardiomyopathy (HCM/DCM/NDLVC/ARVC/RCM, as well as patients with myocarditis, sarcoidosis, amyloidosis, mitochondrial diseases, or other (rare) forms of myocardial disease). For diseases with a potential hereditary background, this includes both genotype-positive patients with identified P/LP variant and gene-elusive patients without a known genetic cause. Individuals who are genotype-positive but phenotype-negative, identified either through cascade screening or as an incidental finding, and who are at risk of developing cardiomyopathy. Selected family members of gene-elusive cardiomyopathy patients, per clinical judgment e.g., those with a clear positive family history and phenotype suggestive of inherited cardiomyopathy, without phenotype, who are at risk of developing cardiomyopathy.

### Registry design

**Fig. 1:**
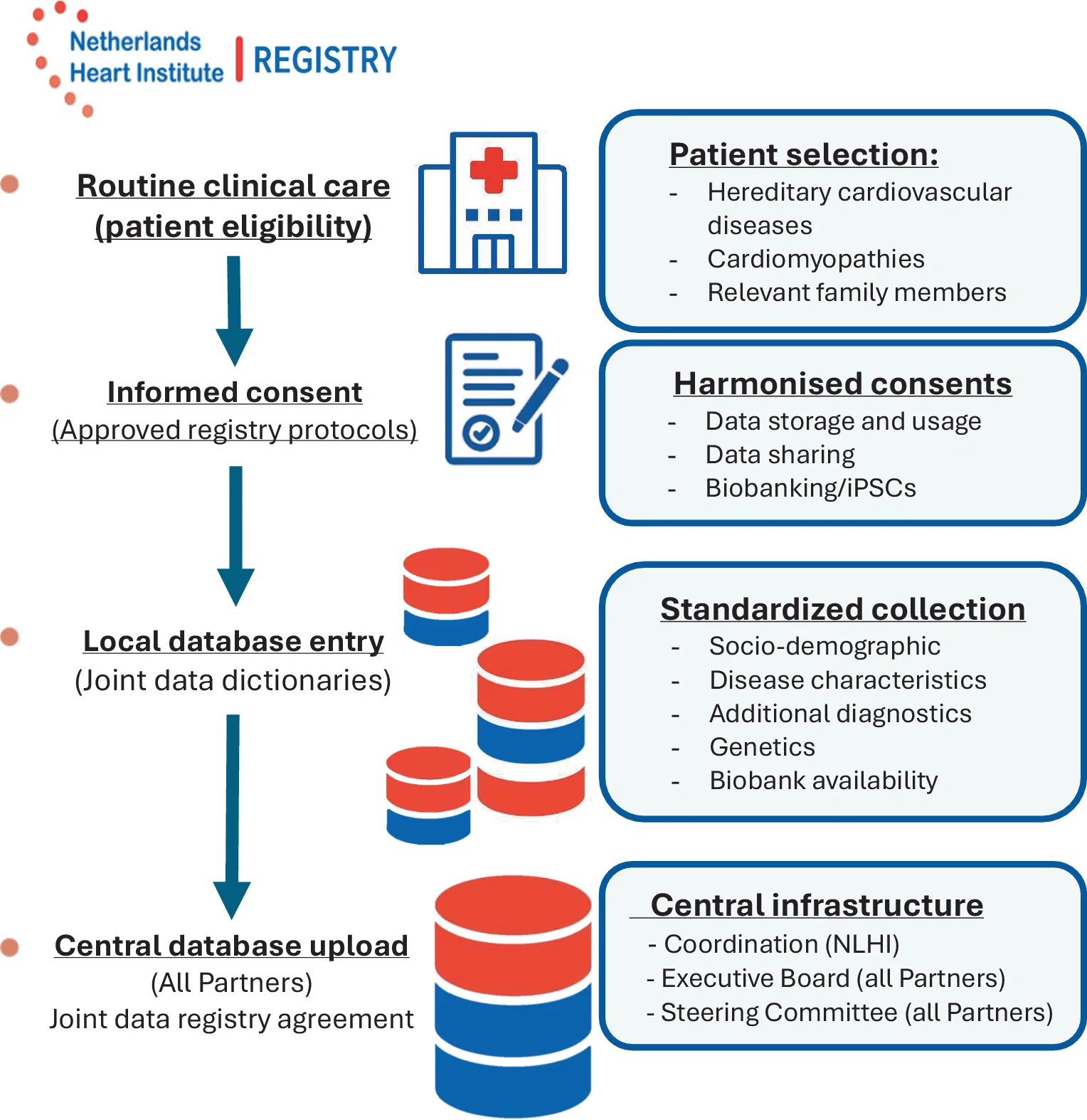
Design and flowchart of the Dutch Cardiomyopathy Registry

Patients will be approached in routine clinical care. Harmonized informed consent will facilitate local database entry (manual or automatic) and standardized data collection. Periodic uploads will be performed to the central database. A central overview of the infrastructure and procedures is provided (Fig. 1).

### Data collection

Subject data are collected by researchers in the study centers using electronic case record forms (eCRF) hosted in Castor [14] or REDCap (Research Electronic Data Capture, Vanderbilt University, Nashville, TN, USA). [15] The complete current data dictionary and data collection instruments are shown in Supplementary File 1 and are listed (and updated when needed) on the NLHI website. In short, baseline data consists of socio-demographics, lifestyle factors, genetic test performed and its results, cardiomyopathy phenotype, symptoms, medication use, device implantations, and other cardiac interventions (e.g. (supra)ventricular catheter ablations, percutaneous interventions of coronaries or valves, cardiac surgery), and diagnostic tests. Test results are collected at first presentation and at every follow-up visit, including: electrocardiography, echocardiography, cardiac magnetic resonance imaging, Holter monitoring, laboratory parameters, exercise testing, computed tomography scans, bone scintigraphy, ventricular/coronary cine-angiograms, and cardiac tissue from biopsy or surgery. Outcome data that are collected are (among others): clinical performance status (e.g., NYHA class and optional quality of life scores), heart failure onset, (heart failure) hospitalization, atrial arrhythmias, (non-)sustained ventricular arrhythmias, Implantable Cardioverter Defibrillator (ICD) implantation, ICD therapy, left ventricular assist device implantation, heart transplantation and (cardiac) death. Follow-up will ideally follow current guideline recommendations and local care paths through regular clinical visits.

### Governance, data sharing, and logistics

Governance is coordinated by the NLHI through an Executive Board with founding partners, alongside domain-specific steering committees that oversee data quality, proposals, etc., through regular meetings. Central data are securely hosted by NLHI with tiered access, shared only after steering committee and individual center approval, governed by local informed consent and a national joint data registry agreement to ensure compliant and collaborative use. See Supplemental File 2 for full details.

### Potential of biobanking

Virtual biobanking will be optional for all centers and will be performed in a decentralized manner under local existing biobanking protocols. Sample availability will be visible within the DCR and can be part of study requests. Sample requests and usage will, by definition, follow local protocols, with a future standard operating procedure in place for joint national requests. Any sample requests within a study proposal through the DCR will first be approved by the steering committee. Afterwards, each local center will proceed to final local approval and distribution of samples. Additionally, registry participants who provided informed consent to be contacted for future cardiovascular studies will be informed about the possibility of donating potential future cardiovascular residual tissue (for instance, if spare material becomes available through cardiovascular surgery or autopsy) to the Netherlands Heart Tissue Bank (NLHI-HTB), which is also hosted by the NLHI (more details at www.hearttissuebank.nl) [16].

### Study proposals

**Fig. 2:**
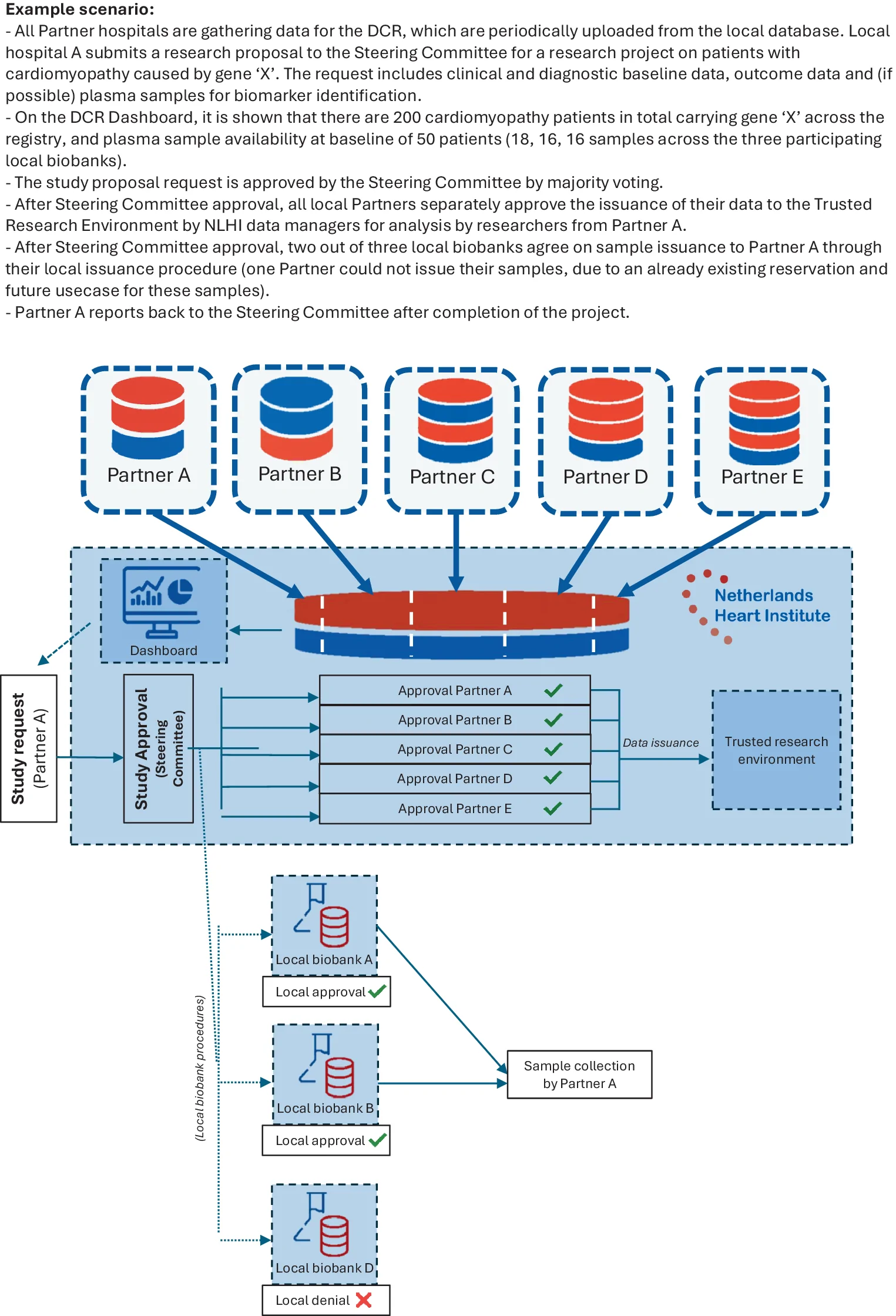
Example of initial governance of the Dutch Cardiomyopathy Registry and local biobanks

Researchers from all partners can submit study proposals to the steering committee. Study proposals include applicants, research question, requested data/samples, analysis plan, and publication plan. A standardized form will be available on the NLHI website. Study proposals will be reviewed at each steering committee meeting. When approved by the Steering Committee, local partners will have the opportunity to approve or deny their data issuance. If the study proposal is approved and biobank samples are also requested, the local biobank procedure will be activated. An example scenario is shown in Fig. 2. Non-partners can submit a study proposal in collaboration with at least one partner.

## Discussion

The DCR is a new Dutch multi-center cardiomyopathy registry with the potential of encompassing the full spectrum of cardiomyopathies. The DCR benefits from collaborative efforts that have been established within the NLHI and the Dutch Cardiovascular Alliance (DCVA) consortia, which have been funded by the Dutch Heart Foundation and other funders in the past [17, 18]. The DCR addresses an important unmet need, as there is currently a paucity of well-phenotyped, longitudinal datasets that include genetic test results spanning cardiomyopathy phenotypes. The Netherlands’ clinical infrastructure is ideal, as genetic testing is reimbursed and performed only in university medical centers (UMCs). The DCR, therefore, will cover a substantial amount of genotype-positive cardiomyopathy patients, genotype-positive phenotype-negative individuals, and gene-elusive patients. Furthermore, all centers are regional reference centers, also representing official national expertise centers and including the national members of ERN GUARD-Heart [19]. Outreach through patients, care networks, and patient organizations will be done to optimize inclusion.

The current situation, with multiple isolated registry protocols working in parallel, is sub-optimal in particular for rare diseases like cardiomyopathies. Collaboration in this area is key, which in the Netherlands is commonly organized through the NLHI. The current alignment of data collection for this domain will hopefully serve as a model for similar future research initiatives. Although a current limitation is the inclusion of only HCM and DCM patients and variant-carriers, the DCR will likely facilitate broader cardiomyopathy groups in the near future. Of note, the Netherlands ACM Registry, another cardiomyopathy registry originating in the Netherlands, will be maintained in its current form, given its partnerships with international counterparts [20]. Possible expansion of the DCR to include the Netherlands ACM Registry will be discussed and communicated with collaborating partners, as there is considerable overlap between phenotypes and specific variants.

As we enter the era of precision therapy and genotype- and phenotype-specific risk stratification in cardiomyopathies, the need for prospective registries of these patients is growing. This will allow further risk stratification, prognostication, and identification of cardiomyopathy patients who are most likely to benefit from new and existing treatment options [21, 22].

Currently, other registry initiatives have already proven their worth for cardiomyopathies, distinguishing multi-modal broad biobanks, genetics-first registries, dedicated cardiomyopathy registries, and even a recently established patient-driven registry (Tab. 2; [20, 23–27]).^.^ It is therefore important to emphasize unique aspects of the DCR. Deep phenotyping, including genetic testing, is standard of care in the Netherlands and will be supported by sophisticated, harmonized national data dictionaries and consents. Furthermore, the possibilities of data and material sharing amongst groups and local access to raw ECG/imaging data under the same national law, agreements, and uniform and harmonized informed consents (including biobank samples) are unique and important future aspects of this database. These aspects will make this registry a welcome addition and potential collaborative entity for other larger-scale research initiatives, including the recently initiated ESC GRASP initiative (https://www.escardio.org/Research/registries/global-registries-and-surveys-programme).

**Table 2:** Overview of known national and international registries.

Name	Core Data	Data Link
*Multi-Modal Broad Biobanks (incl cardiomyopathy)*		
UK Biobank	EHR (ICD-10); CMR (~40 k); WES (200 k); arrays; biomarkers; PROs	https://biobank.ndph.ox.ac.uk/showcase/
Tohoku Medical Megabank Project	Genome; methylome; proteome; surveys; EHR-ICD	https://www.megabank.tohoku.ac.jp/english/
Penn Medicine Biobank	EHR (ICD); labs; CMR/echo; arrays; WES	https://pennmedicine.org/biobank
*Genetics-first registries (incl cardiomyopathy)*		
BioBank Japan	Clinical data; WGS; serum	https://biobankjp.org/en/
China Kadoorie Biobank	Questionnaires; physicals; blood; genotyping	https://www.ckbiobank.org/site/
Generation Scotland	EHR linkage; genotyping; questionnaires	https://www.ed.ac.uk/generation-scotland
FinnGen	National health registers (ICD, procedures), genotyping	https://www.finngen.fi/en
All of Us Research Program	EHR (ICD); surveys; physicals; WGS	https://www.researchallofus.org/data-tools/data-snapshots/
Million Veteran Program	EHR (ICD/CPT); surveys; arrays; WGS	https://www.research.va.gov/mvp/
MyCode Community Health Initiative	EHR (ICD); arrays; WES	https://research.geisinger.org/mycode/
Estonian Biobank	EHR (ICD); arrays; WGS	https://genomics.ee/estonian-biobank
*Dedicated Cardiomyopathy Registries*		
Sarcomeric Human Cardiomyopathy Registry (SHARE)	Clinical HCM/DCM/ARVC phenotypes; family pedigrees; echo/CMR; genotyping	https://www.theshareregistry.org/
ESC EORP Cardiomyopathy & Myocarditis Registry	Demographics; ECG; histology; CMR & LGE; genotyping	https://www.escardio.org/Research/registries/Cardiomyopathy-and-Myocarditis-Registry
National Cardiac Inherited Diseases Registry NZ	Clinical diagnoses; family screening; genotyping	https://www.health.govt.nz/our-work/inherited-cardiac-diseases
Hypertrophic Cardiomyopathy Registry (HCMR)	Echo; CMR cine, LGE, T1 mapping; genotyping; NT-proBNP; hs-cTnT	https://www.jacc.org/doi/10.1016/j.jacc.2019.08.1057
Genetic Cardiomyopathy Registry (by patients)	Self-reported diagnosis; family history; genotyping; PRO surveys	https://geneticcardiomyopathyregistry.org/
Pediatric Cardiomyopathy Registry	Clinical exam; functional status; annual echo; genotype-phenotype sub-studies	https://clinicaltrials.gov/ct2/show/NCT00005391
China Hypertrophic Cardiomyopathy Project	Echo; ECG; CMR subset; genotyping	https://bmcmedgenet.biomedcentral.com/articles/10.1186/s12881-024-01456-7
ChinaCORE-ACM Registry	Clinical & ECG data; echo & CMR; gene panels	https://www.jacc.org/doi/10.1016/j.jacasi.2025.04.005
Netherlands ACM Registry	Clinical data (including outcomes); ECG; Holter; pathology; echo; CMR; EP studies; ICD interrogation; exercise info	https://www.acmregistry.nl/en/home2/
North American ARVC Registry	Clinical data ECG; Holter; echo; CMR; gene panels	https://www.heartrhythmjournal.org/article/S1547-5271(15)00723-1/fulltext

### Future perspectives

The DCR is currently comprised of a central database, fed by local decentralized DCR databases. The central database is expected to be embedded in the future in the Netherlands Heart Registration (NHR). Using the NHR pseudonymization methods, this transition will allow linkage with other NHR registries and ultimately embedding in the infrastructure of the DCVA Health Data Hub, developed in the HEART4DATA consortium [28]. This hub includes procedures for linkage with the Central Bureau of Statistics (CBS) and Dutch Hospital Data and all components needed for conducting registry-based RCTs, such as legal frameworks, contracts, and IT infrastructure, including randomization tools and eCRF’s. Embedding the DCR in this infrastructure will likely increase automated linkage, create synergy between current initiatives, speed up many research projects, and hopefully will be the first of many research registries joining this framework.

Of note, the DCR will be directly incorporated as one of the domains of the planned CardioResource initiative, supported by funding from the Dutch Research Council (NWO) [29]. CardioResource will comprise a sustainable state-of-the-art longitudinal registry infrastructure encompassing patients with different heritable cardiac conditions from all Dutch UMCs, building on existing resources across the disease categories, such as the DCR. It will enable access to diverse data types, including clinical data extracted from electronic health records (EHR), genetic and genomic data (clinical and research data), cardiac imaging and ECG data, available patient biomaterials, and wearables datasets. The collected DCR data will serve as a valuable benchmark for the automated data-extraction procedures that will be developed within CardioResource. Of note, CardioResource will also include new governance procedures coordinated through NLHI, replacing older governance agreements for the applicable disease domains where appropriate.

Lastly, the DCR is installed to not only accommodate HCM/DCM, but has the potential to accommodate other (hereditary) myocardial diseases (covered by the included registry protocols), including, for example, infiltrative cardiac diseases like sarcoidosis and amyloidosis, other rare cardiomyopathies (restrictive, metabolic/mitochondrial), myocarditis, and Takotsubo (stress) cardiomyopathy. Also, collaboration with the multi-center Netherlands ACM Registry and PLN Registry will be explored.

Given the low patient numbers across these diseases, structures like this could unite national stakeholders, without requiring new research agreements or infrastructure building. We hope this initiative is the start of many national collaborations, including the potential future addition of non-UMCs. Within the recently funded PRECISE consortium, these initiatives will likely flourish and will benefit from this concerted action [30].

## Conclusion

The field of cardiomyopathies is entering a defining stage, as our understanding of these diseases improves, enabling better treatment and prediction. A major challenge in this growing patient population is the timely identification of individuals at risk of adverse events and disease progression. In addition, preventive treatment based on genetic background and phenotype requires data on genetics, co-morbidities, and the natural history of the disease. Longitudinal, high-quality, robust datasets and biobanks are needed to tackle these outstanding challenges. This NLHI initiative will enable expansion of domains within the cardiovascular disease space to study overlapping disease manifestations, the role of genetics, phenotype-negative DNA variant carriers, and non-genetic factors. The DCR will contribute to these dynamic areas of research and strives to be an example of intense and fruitful collaboration.

## Supplementary Information

ESM1: Supplementary material 1

ESM2: Supplementary material 2

ESM3: Supplementary material 3

## Data Availability

All data supporting the findings of this work are included in the article.
