# Supplementary material for: The Dutch cardiomyopathy registry (DCR); rationale and registry design
Source: Neth Heart J. 2026 Aug 10;34(9):288–98. doi: 10.1007/s12471-026-02063-5 (PMC13504138; doi:10.1007/s12471-026-02063-5)
Supplement: Supplementary file 1 [file 12471_2026_2063_MOESM1_ESM.pdf]

Castor CDMS

Dutch Cardiomyopathy Registry (DCR)

Case Report Forms

Study ID	B8FEBA4C-E68F-48DA-BCA5-5CC82B915747
Study Version	76.51
Generated at	20-Nov-2025 15:37:56
Generated by	Michiel Minten

Contents

Visits 2
Consent . . . 2
1. Informed Consent Items . . . 2
Baseline (first presentation in this center) . . . 6
2. Inclusion information . . . 6
Baseline (DCM/HNDC) . . . 10
3. Clinical presentation . . . 10
4. Risk factors . . . 12
5. Medication . . . 16
Baseline (HCM) . . . 17
6. Clinical presentation . . . 17
7. Risk factors . . . 19
8. Medication . . . 23
Baseline (Family Member CMP Patient) . . . 24
9. Clinical Presentation . . . 24
10. Risk Factors . . . 26
11. Medication . . . 30
Medical Events/history & Family history . . . 31
12. Cardiac History/Events . . . 31
13. Non Cardiac History/Events . . . 47
14. Hospitalisation (incl Cardiac Arrest /LTA/LVAD/HTx) . . . 70
15. Devices & device therapy . . . 73
16. (Loss to) follow-up . . . 82
17. Mortality . . . 85
18. Family History . . . 88
Additional diagnostics . . . 91
19. Lab . . . 91
20. ICD readout . . . 93
21. ECG . . . 94
22. Echocardiography . . . 95
23. Holter . . . 96
24. CMR . . . 97
25. Genetics . . . 98
Medical consultations at Follow-up . . . 114
26. Medical consultations . . . 114
27. Medication . . . 116
Biobanking . . . 117
28. Biobanking . . . 117

Repeating Data 119
Cardiac magnetic resonance (CMR) . . . 119
1. Cardiac magnetic resonance (CMR) . . . 119
Echocardiography . . . 145
1. Echocardiography . . . 145
ICD readout . . . 170
1. ICD readout . . . 170
Medication . . . 175
1. Medication . . . 175
Familiy Member Sudden Cardiac Death . . . 196
1. Family Member Sudden Cardiac Death . . . 196
Holter . . . 198
1. Holter . . . 198
Hospitalization . . . 206
1. Hospitalization details . . . 206
Medical consultations . . . 213
1. Consultations . . . 213

Laboratory 221
1. Laboratory 221
Electrocardiography 229
1. Electrocardiography 229
Cardiac implantable electronic devices 236
1. Cardiac implantable electronic devices 236

Option Groups **238**
Supraventricular tachycardia conclusion 238
Type cancer 238
type of SVT 238
GENE_LIST 238
HF etiologie 239
conduction abnormalities 239
Rhythm during echo 240
auto-immune diseases 240
Beta Blocker Types 240

Visits

Consent

Consent

1. Informed Consent Items

The participant has consented to:

1.1 **Collect data from mediacal record***

- Yes
- No

Field type Radiobutton
Option group Yes/no
Variable name IC_collect_data_medical_file

1.2 **Request information GP or medical specialists***

- Yes
- No

Field type Radiobutton
Option group Yes/no
Variable name IC_request_GP

1.3 **Store body material***

- Yes
- No

Field type Radiobutton
Option group Yes/no
Variable name IC_store_body_material

1.4 **Extra venipuncture and blood sampling***

- Yes
- No

Field type	Radiobutton	Variable name	IC_venipunct_blood_samp
Option group	Yes/no		

1.5 **DNA research***

- ○ Yes
- ○ No

Field type	Radiobutton	Variable name	IC_DNA_research
Option group	Yes/no		

1.6 **Make and perform research on iPSC's***

- ○ Yes
- ○ No

Field type	Radiobutton	Variable name	IC_iPSCs
Option group	Yes/no		

1.7 **Make and perform research on organoids***

- ○ Yes
- ○ No

Field type	Radiobutton	Variable name	IC_organoids
Option group	Yes/no		

1.8 **Report incidental findings to participant***

- ○ Yes
- ○ No

Field type	Radiobutton	Variable name	IC_report_incident_participant
Option group	Yes/no		

1.9 **Report incidental findings to GP or medical specialists***

- ◯ Yes
- ◯ No

Field type: Radiobutton
Option group: Yes/no
Variable name: IC_report_incident_GP

1.10 **Receive questionnaires***

- ◯ Yes
- ◯ No

Field type: Radiobutton
Option group: Yes/no
Variable name: IC_questionnaires

1.11 **Link medical data with Dutch organisations or registries***

- ◯ Yes
- ◯ No

Field type: Radiobutton
Option group: Yes/no
Variable name: IC_link_data

If **1.11 Link medical data with Dutch organisations or r...** is equal to **Yes** answer this question:

1.11.1 **Link to which Dutch organisations or registries?***

- ☐ Basis Registratie Personen (BRP)
- ☐ Centraal Bureau Statistiek (CBS)
- ☐ Nederlandse Kankerregistratie (NKR)
- ☐ Pathologisch Anatomisch Landelijk Geautomatiseerd Archief (PALGA)
- ☐ Nederlandse Hart Registratie (NHR)
- ☐ Hartenbank
- ☐ Hersenbank

Field type: Checkbox
Option group: Link_data_organisations
Variable name: IC_link_data_which

1.12 **Share data or body material with other institutions or commercial companies***

- ○ Yes
- ○ No

Field type: Radiobutton
Option group: Yes/no
Variable name: IC_share_institutions_companies

1.13 **Share data or body material within the EU***

- ○ Yes
- ○ No

Field type: Radiobutton
Option group: Yes/no
Variable name: IC_share_within_EU

1.14 **Share data or body material outsidethe EU***

- ○ Yes
- ○ No

Field type: Radiobutton
Option group: Yes/no
Variable name: IC_share_outside_EU

1.15 **Use of data and body material for other research***

- ○ Yes
- ○ No

Field type: Radiobutton
Option group: Yes/no
Variable name: IC_use_other_research

Baseline (first presentation in this center)

Baseline (first presentation in this center)

2. Inclusion information

In this form, general information is collected e.g. to gain insights into where the individual presents themselves within this hospital and to determine whether the individual has a medical history of heart disease(s).

2.1 **Date medical information checked***
essential for future analyses, as it represents the most recent point up to which information is available.

DD MM YYYY

Error message shown if field's value is greater than **NOW**

date cannot be in the future

Field type Date

Variable name Inc_d

2.2 **The included subject is a:***
Persons without (previous) heart failure / cardiomyopathy: e.g. family members

- ○ Patient with (previous) heart failure/cardiomyopathy
- ○ Person without (previous) heart failure/cardiomyopathy

Field type Dropdown
Option group type subject

Variable name Incl_type

If date is not exactly known, use the median date as an estimation (15th of the month if exact day is unknown, or July 1st when only the year is known). If completely unknown, use 01-01-1800.

If **2.2 The included subject is a:** is equal to **Patient with (previous) heart failure/cardiomyopathy** answer this question:

2.2.2 date check

Calculated value

Error message shown if field's value is equal to **0**

Date "medical information checked" cannot be earlier than date of first presentation/diagnosis in this hospital

Field type: Calculation
Variable name: HF_inclusionform_check_diagowncentrum_d

If **2.2 The included subject is a:** is equal to **Patient with (previous) heart failure/cardiomyopathy** answer this question:

2.2.3 **First presentation with heart failure or cardiomyopathy in this hospital***

- ○ Hospitalization
- ○ Emergency department (SEH/EHH) without hospitalization
- ○ Cardiology outpatient
- ○ Unknown

Field type: Dropdown
Option group: HF first presentation
Variable name: Incl_HF_first_pres

If **2.2 The included subject is a:** is equal to **Patient with (previous) heart failure/cardiomyopathy** answer this question:

2.2.4 **Has the diagnosis of heart failure/cardiomyopathy already been established elsewhere?***

- ○ Yes
- ○ No

Field type: Radiobutton
Option group: Yes/No
Variable name: Incl_HF_previous

If date is not exactly known, use the median date as an estimation (15th of the month if exact day is unknown, or July 1st when only the year is known). If completely unknown, use 01-01-1800.

If **2.2.4 Has the diagnosis of heart failure/cardiomyopat...** is equal to **Yes** answer this question:

2.2.4.2 date check — Calculated value

Error message shown if field's value is equal to **0**

Date diagnosis in this hospital cannot be earlier than date of first presentation/diagnosis elsewhere

Field type	Calculation	Variable name	HF_diagnosis_check_d

If 2.2.4 **Has the diagnosis of heart failure/cardiomyopat...** is equal to **Yes** answer this question:

2.2.4.3 date check

Calculated value

Error message shown if field's value is equal to **0**

Date "medical information checked" cannot be earlier than date of first presentation/diagnosis

Field type	Calculation	Variable name	HF_inclusionform_check_diagfirst_d

If 2.2.4 **Has the diagnosis of heart failure/cardiomyopat...** is equal to **Yes** answer this question:

2.2.4.4 **Location first diagnosis heart failure/cardiomyopathy***

- ○ 1e lijn (General practitioner)
- ○ 2e lijn (General hospital)
- ○ 3e lijn (Specialized hospital)
- ○ Unknown

Field type	Dropdown	Variable name	Incl_HF_first_loc
Option group	eerste diagnose HF locatie		

If 2.2 **The included subject is a:** is equal to **Patient with (previous) heart failure/cardiomyopathy** answer this question:

2.2.5 **What is the primary ***cause(s)*** of heart failure/cardiomyopathy (diagnosis at first presentation in this hospital)***
Please also include diagnosis if it has been made within 6 months after first presentation

This checkbox field has too many options to list here. See Option Group - HF etiologie for its options.

ⓘ **Warning** message shown if field's value is equal to **-1**

are you sure?

Field type Checkbox
Option group HF etiologie

Variable name Incl_HF_type

If 2.2.5 What is the primary ***cause(s)*** of heart fa... is checked on **Other reasons** answer this question:

2.2.5.1 **What is the primary cause of heart failure/cardiomyopathy***

Field type Multiline Textfield

Variable name Incl_HF_type_other

Baseline (DCM/HNDC)

Baseline (DCM/HNDC)

3. Clinical presentation

3.1 **First outpatient clinic visit with diagnosis of DCM/HNDC in this centre (T=0)***
based on expert opinion

DD MM YYYY

Error message shown if field's value is greater than **NOW**

No future dates can be entered

Field type Date

Variable name DCM_CP_d

3.2 **Referral source***

- General physician (huisarts)
- Cardiology outpatient
- After cardiology admission
- Clinical Geneticist
- Other specialism
- Unknown

Field type Radiobutton
Option group Referral source type

Variable name DCM_CP_refer

3.3 date check

Calculated value

Error message shown if field's value is equal to **Error**

Date of first outpatient clinic visit must be later than date first presentation at this hospital

Information message shown if field's value is equal to **Okay**

Add clinical presentation report for this date below.

Field type Calculation

Variable name DCM_first_outpatient_check

3.4 Add clinical presentation
(linked Repeating Data Form: "Medical consultations")

Created on	Information changed after Date data cleaned?	Variables changed? (report numbers of variables)	Date of consultation	Type of consultation	Consult moment	NYHA classification

Field type Repeated measures

Baseline (DCM/HNDC)

4. Risk factors

4.1 Add Intoxication information based on baseline date:

Summary

Field type: Summary

4.2 **Smoking***

- Non smoker
- Former smoker
- Current smoker

Field type: Dropdown
Option group: Smoking
Variable name: DCM_intox_smok

If **4.2.1 Ever smoked** is equal to **1** answer this question:

4.2.1.1 **Smoking packyears***
1 pack year = 20 cigarettes/day OR 2.5 packs of shaque/week OR 7 sigars/day for 1 year. Calculator available here: https://www.smokingpackyears.com/

packyears

Field type: Numeric field
Variable name: DCM_intox_smok_py

If **4.2 Smoking** is equal to **Former smoker** answer this question:

4.2.2 Year of last smoking

YYYY

Field type: Year
Variable name: DCM_intox_smok_fd

4.3 **Alcohol usage***
Current or previous alcohol usage

- ○ Yes
- ○ No
- ○ Unknown

Field type: Radiobutton
Option group: Yes/No/Unknown
Variable name: DCM_intox_alc

If **4.3 Alcohol usage** is equal to **Yes** answer this question:

4.3.1 **Heavy alcohol usage***
Heavy = 21 units men & 17 units women/ week (0,8g alc = 1 unit).

- ○ Yes
- ○ No
- ○ Unknown

Field type: Radiobutton
Option group: Yes/No/Unknown
Variable name: DCM_intox_alc_h

If **4.3 Alcohol usage** is equal to **Yes** answer this question:

4.3.2 How many units of alcohol per week?

[ ]

Field type: Numeric field
Variable name: DCM_intox_alc_u

If **4.3 Alcohol usage** is equal to **Yes** answer this question:

4.3.3 **Alcohol use for how many years?***

[ ] years

Field type: Numeric field
Variable name: DCM_intox_alc_years

4.4 **Drugs usage (current and/or previous)***

- ○ Yes
- ○ No
- ○ Unknown

Field type	Radiobutton	Variable name	DCM_intox_drugs
Option group	Yes/No/Unknown		

If **4.4 Drugs usage (current and/or previous)** is equal to **Yes** answer this question:

4.4.1 **Drugs usage current or previous***

- ○ Previous drugs usage
- ○ Current drugs usage
- ○ Unknown

Field type	Radiobutton	Variable name	DCM_intox_drugs_current
Option group	previous or current		

If **4.4 Drugs usage (current and/or previous)** is equal to **Yes** answer this question:

4.4.2 **Drugs Type***

- ☐ Cocaine
- ☐ Cannabis/Marijuana
- ☐ Heroine
- ☐ XTC
- ☐ Andere drugs, namelijk:

Field type	Checkbox	Variable name	DCM_intox_drugs_type
Option group	Drugs soort (Q)		

If **4.4.2 Drugs Type** is checked on **Andere drugs, namelijk:** answer this question:

4.4.2.1 **Specify other drugs types***

Field type Textfield Variable name DCM_intox_drugs_type_oth

If 4.4 **Drugs usage (current and/or previous)** is equal to **Yes** answer this question:

4.4.3 **Frequency of drug use***

Field type Textfield Variable name DCM_intox_drugs_freq

If 4.4 **Drugs usage (current and/or previous)** is equal to **Yes** answer this question:

4.4.4 **Quantity of drug use***

Field type Textfield Variable name DCM_intox_drugs_quant

4.5 **Exercise before development of phenotype***
Exercise in the last year before 'development of phenotype', meaning "average regular exercise when healthy". If it is likely that symptoms were present for a longer time, before first consultation, please consdier that as expression of the phenotype and not the period to consider for this question.

- Low (<2.5 hrs moderate activity/week)
- Moderate (>=2.5 hrs moderate or >0 but <2.5hrs high activity/week)
- High (>=2.5 hrs high or >5 moderate activity/week)

Field type Radiobutton Variable name DCM_RF_exercise
Option group Exercise before phenotype

Please lock this form if it has been completed and is filled in correctly

Baseline (DCM/HNDC)

5. Medication

5.1 Add Medication information based on **the exact T=0 moment of the DCM/HNDC outpatient clinic** in this centre
Atleast at full list of medication before the outpatient clinic visit! If no medication information is available, add report on the date of T=0 and set date of medication in the report as missing.

Summary

Field type: Summary
Variable name: NICMP_add_med

5.2 **Baseline medication checked***
if baseline information is checked but information is not available please choose "Yes", create the report and set medication date to user missing.

○ Yes
○ No

Field type: Radiobutton
Option group: Yes/No
Variable name: DCM_med_avail

If 5.2 **Baseline medication checked** is equal to **Yes**:

5.2.1 Add medication before and after outpatient visit
(linked Repeating Data Form: "Medication")

Created on	Date Medication	Medication moment	Beta-blocker

Field type: Repeated measures

Baseline (HCM)

Baseline (HCM)

6. Clinical presentation

6.1 **First outpatient clinic visit with diagnosis of HCM in this centre (T=0)***

DD MM YYYY

Error message shown if field's value is greater than **NOW**

No future dates can be entered

Field type: Date

Variable name: HCM_CP_d

6.2 **Referral source***

- General physician (huisarts)
- Cardiology outpatient
- After cardiology admission
- Clinical Geneticist
- Other specialism
- Unknown

Field type: Radiobutton

Option group: Referral source type

Variable name: HCM_CP_refer

6.3 date check

Calculated value

Error message shown if field's value is equal to **Error**

Date of first outpatient clinic visit must be later than date first presentation at this hospital

Information message shown if field's value is equal to **Okay**

Add clinical presentation report for this date below.

Field type Calculation

Variable name HCM_first_outpatient_check

6.4 Add clinical presentation
(linked Repeating Data Form: "Medical consultations")

Created on	Information changed after Date data cleaned?	Variables changed? (report numbers of variables)	Date of consultation	Type of consultation	Consult moment	NYHA classification

Field type Repeated measures

Baseline (HCM)

7. Risk factors

7.1 Add Intoxication information based on baseline date:

Summary

Field type: Summary

7.2 **Smoking***

- Non smoker
- Former smoker
- Current smoker

Field type: Dropdown
Option group: Smoking
Variable name: HCM_intox_smok

If 7.2.1 **Ever smoked** is equal to **1** answer this question:

7.2.1.1 **Smoking packyears***
1 pack year = 20 cigarettes/day OR 2.5 packs of shaque/week OR 7 sigars/day for 1 year. Calculator available here: https://www.smokingpackyears.com/

packyears

Field type: Numeric field
Variable name: HCM_intox_smok_py

If 7.2 **Smoking** is equal to **Former smoker** answer this question:

7.2.2 Year of last smoking

YYYY

Field type: Year
Variable name: HCM_intox_smok_fd

7.3 **Alcohol usage***
Current or previous alcohol usage

- ○ Yes
- ○ No
- ○ Unknown

Field type: Radiobutton
Option group: Yes/No/Unknown
Variable name: HCM_intox_alc

If **7.3 Alcohol usage** is equal to **Yes** answer this question:

7.3.1 **Heavy alcohol usage***
Heavy = 21 units men & 17 units women/ week (0,8g alc = 1 unit).

- ○ Yes
- ○ No
- ○ Unknown

Field type: Radiobutton
Option group: Yes/No/Unknown
Variable name: HCM_intox_alc_h

If **7.3 Alcohol usage** is equal to **Yes** answer this question:

7.3.2 How many units of alcohol per week?

[ ]

Field type: Numeric field
Variable name: HCM_intox_alc_u

If **7.3 Alcohol usage** is equal to **Yes** answer this question:

7.3.3 **Alcohol use for how many years?***

[ ] years

Field type: Numeric field
Variable name: HCM_intox_alc_years

7.4 **Drugs usage (current and/or previous)***

- ○ Yes
- ○ No
- ○ Unknown

Field type	Radiobutton	Variable name	HCM_intox_drugs
Option group	Yes/No/Unknown		

If **7.4 Drugs usage (current and/or previous)** is equal to **Yes** answer this question:

7.4.1 **Drugs usage current or previous***

- ○ Previous drugs usage
- ○ Current drugs usage
- ○ Unknown

Field type	Radiobutton	Variable name	HCM_intox_drugs_current
Option group	previous or current		

If **7.4 Drugs usage (current and/or previous)** is equal to **Yes** answer this question:

7.4.2 **Drugs Type***

- ☐ Cocaine
- ☐ Cannabis/Marijuana
- ☐ Heroine
- ☐ XTC
- ☐ Andere drugs, namelijk:

Field type	Checkbox	Variable name	HCM_intox_drugs_type
Option group	Drugs soort (Q)		

If **7.4.2 Drugs Type** is checked on **Andere drugs, namelijk:** answer this question:

7.4.2.1 **Specify other drugs types***

Field type Textfield Variable name HCM_intox_drugs_type_ot
h

If 7.4 **Drugs usage (current and/or previous)** is equal to **Yes** answer this question:

7.4.3 **Frequency of drug use***

Field type Textfield Variable name HCM_intox_drugs_freq

If 7.4 **Drugs usage (current and/or previous)** is equal to **Yes** answer this question:

7.4.4 **Quantity of drug use***

Field type Textfield Variable name HCM_intox_drugs_quant

7.5 **Exercise before development of phenotype***
Exercise in the last year before 'development of phenotype', meaning "average regular exercise when healthy". If it is likely that symptoms were present for a longer time, before first consultation, please consdier that as expression of the phenotype and not the period to consider for this question.

- ○ Low (<2.5 hrs moderate activity/week)
- ○ Moderate (>=2.5 hrs moderate or >0 but <2.5hrs high activity/week)
- ○ High (>=2.5 hrs high or >5 moderate activity/week)

Field type Radiobutton Variable name HCM_RF_exercise
Option group Exercise before phenotype

Please lock this form if it has been completed and is filled in correctly

Baseline (HCM)

8. Medication

8.1 Add Medication information based on **the exact T=0 moment of the HCM outpatient clinic** in this centre
Atleast at full list of medication before the outpatient clinic visit! If no medication information is available, add report on the date of T=0 and set date of medication in the report as missing.

Summary

Field type: Summary

8.2 **Baseline medication checked***
if baseline information is checked but information is not available please choose "Yes", create the report and set medication date to user missing.

○ Yes
○ No

Field type: Radiobutton
Option group: Yes/No
Variable name: HCM_med_avail

If **8.2 Baseline medication checked** is equal to **Yes**:

8.2.1 Add medication before and after outpatient visit
(linked Repeating Data Form: "Medication")

Created on	Date Medication	Medication moment	Beta-blocker

Field type: Repeated measures

Baseline (Family Member CMP Patient)

Baseline (Family Member CMP Patient)

9. Clinical Presentation

9.1 **First outpatient clinic visit in this centre of Family Member of CMP patient (T=0)***

DD MM YYYY

Error message shown if field's value is greater than **NOW**

No future dates can be entered

Field type: Date

Variable name: nonCMP_CP_d

9.2 **Referral source***

- ○ General physician (huisarts)
- ○ Cardiology outpatient
- ○ After cardiology admission
- ○ Clinical Geneticist
- ○ Other specialism
- ○ Unknown

Field type: Radiobutton

Option group: Referral source type

Variable name: nonCMP_CP_refer

9.3 date check

Calculated value

Error message shown if field's value is equal to **Error**

Date of first outpatient clinic visit must be later than date first presentation at this hospital

Information message shown if field's value is equal to **Okay**

Add clinical presentation report for this date below.

Field type Calculation

Variable name nonCMP_first_outpatient_check

9.4 Add clinical presentation
(linked Repeating Data Form: "Medical consultations")

Created on	Information changed after Date data cleaned?	Variables changed? (report numbers of variables)	Date of consultation	Type of consultation	Consult moment	NYHA classification

Field type Repeated measures

Variable name

Baseline (Family Member CMP Patient)

10. Risk Factors

10.1 Add Intoxication information based on baseline date:

Summary

Field type	Summary	Variable name	

10.2 **Smoking***

- Non smoker
- Former smoker
- Current smoker

Field type	Dropdown	Variable name	nonCMP_intox_smok
Option group	Smoking		

If **10.2.1 Ever smoked** is equal to **1** answer this question:

10.2.1.1 **Smoking packyears***
1 pack year = 20 cigarettes/day OR 2.5 packs of shaque/week OR 7 sigars/day for 1 year. Calculator available here: https://www.smokingpackyears.com/

packyears

Field type	Numeric field	Variable name	nonCMP_intox_smok_py

If **10.2 Smoking** is equal to **Former smoker** answer this question:

10.2.2 Year of last smoking

YYYY

Field type	Year	Variable name	nonCMP_intox_smok_fd

10.3 **Alcohol usage***
Current or previous alcohol usage

- Yes
- No
- Unknown

Field type: Radiobutton
Option group: Yes/No/Unknown
Variable name: nonCMP_intox_alc

If **10.3 Alcohol usage** is equal to **Yes** answer this question:

10.3.1 **Alcohol use for how many years?***

[ ] years

Field type: Numeric field
Variable name: nonCMP_intox_alc_years

If **10.3 Alcohol usage** is equal to **Yes** answer this question:

10.3.2 **Heavy alcohol usage***
Heavy = 21 units men & 17 units women/ week (0,8g alc = 1 unit).

- Yes
- No
- Unknown

Field type: Radiobutton
Option group: Yes/No/Unknown
Variable name: nonCMP_intox_alc_h

If **10.3 Alcohol usage** is equal to **Yes** answer this question:

10.3.3 How many units of alcohol per week?

[ ]

Field type: Numeric field
Variable name: nonCMP_intox_alc_u

10.4 **Drugs usage (current and/or previous)***

- ○ Yes
- ○ No
- ○ Unknown

Field type: Radiobutton
Option group: Yes/No/Unknown
Variable name: nonCMP_intox_drugs

If **10.4 Drugs usage (current and/or previous)** is equal to **Yes** answer this question:

10.4.1 **Drugs usage current or previous***

- ○ Previous drugs usage
- ○ Current drugs usage
- ○ Unknown

Field type: Radiobutton
Option group: previous or current
Variable name: nonCMP_intox_drugs_current

If **10.4 Drugs usage (current and/or previous)** is equal to **Yes** answer this question:

10.4.2 **Drugs Type***

- ☐ Cocaine
- ☐ Cannabis/Marijuana
- ☐ Heroine
- ☐ XTC
- ☐ Andere drugs, namelijk:

Field type: Checkbox
Option group: Drugs soort (Q)
Variable name: nonCMP_intox_drugs_type

If **10.4.2 Drugs Type** is checked on **Andere drugs, namelijk:** answer this question:

10.4.2.1 **Specify other drugs types***

Field type Textfield

Variable name nonCMP_intox_drugs_type_oth

If 10.4 **Drugs usage (current and/or previous)** is equal to **Yes** answer this question:

10.4.3 **Frequency of drug use***

Field type Textfield

Variable name nonCMP_intox_drugs_freq

If 10.4 **Drugs usage (current and/or previous)** is equal to **Yes** answer this question:

10.4.4 **Quantity of drug use***

Field type Textfield

Variable name nonCMP_intox_drugs_quant

10.5 **Exercise before development of phenotype***
Exercise in the last year before 'development of phenotype', meaning "average regular exercise when healthy". If it is likely that symptoms were present for a longer time, before first consultation, please consdier that as expression of the phenotype and not the period to consider for this question.

- ○ Low (<2.5 hrs moderate activity/week)
- ○ Moderate (>=2.5 hrs moderate or >0 but <2.5hrs high activity/week)
- ○ High (>=2.5 hrs high or >5 moderate activity/week)

Field type Radiobutton
Option group Exercise before phenotype

Variable name nonCMP_RF_exercise

Please lock this form if it has been completed and is filled in correctly

Baseline (Family Member CMP Patient)

11. Medication

11.1 Add Medication information based on **the exact T=0 moment of the HCM outpatient clinic**
Atleast at full list of medication before the outpatient clinic visit! If no medication information is available, add report on the date of T=0 and set date of medication in the report as missing.

Summary

Field type: Summary
Variable name:

11.2 **Baseline medication checked***
if baseline information is checked but information is not available please choose "Yes", create the report and set medication date to user missing.

- Yes
- No

Field type: Radiobutton
Option group: Yes/No
Variable name: nonCMP_med_avail

If **11.2 Baseline medication checked** is equal to **Yes**:

11.2.1 Add medication before and after outpatient visit
(linked Repeating Data Form: "Medication")

Created on	Date Medication	Medication moment	Beta-blocker

Field type: Repeated measures
Variable name:

Medical Events/history & Family history

Medical Events/history & Family history

12. Cardiac History/Events

Cardiac medical history

12.1 **Date medical information checked***

DD MM YYYY

ⓘ **Information** message shown if field's value is greater than **1800-01-01**

Update medical history until today (also events after inclusion date and after first presentation with heart failure)

Error message shown if field's value is greater than **NOW**

this cannot be a future date!

Field type Date

Variable name MH_car_upd_d

12.2 Date check

Calculated value

Error message shown if field's value is equal to **Error**

Date information checked must be later than date first presentation at this hospital

Field type Calculation

Variable name MH_car_upd_d_check

Ischemia

12.3 **Cardiac ischemia/coronary stenosis assessed?***
by CT-angio, CT-heart, PET, CMR, MIBI, CAG, CABG If coronary artery disease noted in medical history, look for assessment during that period!

- ○ Yes
- ○ No

ⓘ **Warning** message shown if field's value is equal to **0**

This question asks if cardiac ischemia or coronary stenosis is assessed, not if it is present. So select yes if assessments for these findings have been done (more info at i-symbol)

Field type	Radiobutton	Variable name	MH_ischemia_det
Option group	Yes/No		

If **12.3 Cardiac ischemia/coronary stenosis assessed?** is equal to **Yes** answer this question:

12.3.1 **Coronary artery disease (CAD) ever present?***
Noted in medical history. Select the most severe form of CAD that has ever been present in the patient. Significant CAD as noted in medical history, or >70% stenosis on CAG or proven otherwise. Wand onregelmatigheden = no CAD

- ○ significant CAD
- ○ non-significant CAD
- ○ no CAD

Field type	Radiobutton	Variable name	MH_CAD
Option group	CAD		

If **12.3.1 Coronary artery disease (CAD) ever present?** is equal to **significant CAD** answer this question:

12.3.1.1 **CAD location***
Based on SYNTAX classification

- ☐ Left main
- ☐ LAD
- ☐ RCX
- ☐ RCA
- ☐ Graft
- ☐ Other
- ☐ Location unknown

Field type Checkbox
Option group Coronaries

Variable name MH_CADvessel

If 12.3.1 **Coronary artery disease (CAD) ever present?** is equal to **significant CAD** answer this question:

12.3.1.2 **Date of FIRST diagnosis significant CAD***
If date is not exactly known, use the median date as an estimation (15th of the month if exact day is unknown, or July 1st when only the year is known). If completely unknown, use 01-01-1800.

DD MM YYYY

Error message shown if field's value is greater than **NOW**

No future dates can be entered

Field type Date

Variable name MH_CAD_d

If 12.3.1 **Coronary artery disease (CAD) ever present?** is not equal to **significant CAD** answer this question:

12.3.1.3 **Coronary microvascular disease proven***
Only if specifically noted in medical history OR CAG results of CFR <2.0, IMR $\geq$ 25 or negative acetylcholine provocation test (based on ESC guideline CCO 2019). Also known as coronary microcirculatory impairment or NOCAD.

- Yes
- No
- Unknown

Field type Radiobutton
Option group Yes/No/Unknown

Variable name MH_microCAD

If 12.3.1.3 **Coronary microvascular disease proven** is equal to **Yes** answer this question:

12.3.1.3.1 **Date of first diagnosis coronary microvascular disease***
If date is not exactly known, use the median date as an estimation (15th of the month if exact day is unknown, or July 1st when only the year is known). If completely unknown, use 01-01-1800.

DD MM YYYY

Error message shown if field's value is greater than **NOW**

No future dates can be entered

Field type	Date	Variable name	MH_microCAD_d

12.4 **Acute coronary syndrome***
ACS entails STEMI, NSTEMI and unstable angina

- Yes
- No

Field type	Radiobutton	Variable name	MH_ACS
Option group	Yes/No_		

If 12.4 **Acute coronary syndrome** is equal to **Yes** answer this question:

12.4.1 **Acute coronary syndrome type***

- STEMI
- NSTEMI
- Unstable Angina Pectoris
- Unknown

Field type	Radiobutton	Variable name	MH_ACS_type
Option group	ACS types		

If 12.4 **Acute coronary syndrome** is equal to **Yes** answer this question:

12.4 .2 **Date of first diagnosis ACS**
If date is not exactly known, use the median date as an estimation (15th of the month if exact day is unknown, or July 1st when only the year is known). If completely unknown, use 01-01-1800.

DD | MM | YYYY

Error message shown if field's value is greater than **NOW**

No future dates can be entered

Field type: Date
Variable name: MH_ACS_d

12.5 **Percutaneous coronary intervention (PCI)**

- Yes
- No

Field type: Radiobutton
Option group: Yes/No_
Variable name: MH_PCI

If 12.5 **Percutaneous coronary intervention (PCI)** is equal to **Yes** answer this question:

12.5 .1 **Date of first PCI**
If date is not exactly known, use the median date as an estimation (15th of the month if exact day is unknown, or July 1st when only the year is known). If completely unknown, use 01-01-1800.

DD | MM | YYYY

Error message shown if field's value is greater than **NOW**

No future dates can be entered

Field type: Date
Variable name: MH_PCI_d

12.6 **Coronary artery bypass graft (CABG)**

○ Yes

○ No

ⓘ **Information** message shown if field's value is equal to **1**

Add CABG also to Adverse events > Coronary interventions

Field type	Radiobutton	Variable name	MH_CABG
Option group	Yes/No__		

If 12.6 Coronary artery bypass graft (CABG) is equal to **Yes** answer this question:

12.6.1 **Date of first CABG**
If date is not exactly known, use the median date as an estimation (15th of the month if exact day is unknown, or July 1st when only the year is known). If completely unknown, use 01-01-1800.

DD MM YYYY

Error message shown if field's value is greater than **NOW**

No future dates can be entered

Field type	Date	Variable name	MH_CABG_d

Valvular disease

12.7 **Cardiac valve replacement / repair**
noted in medical history

○ Yes

○ No

Field type	Radiobutton	Variable name	MH_VDR
Option group	Yes/No		

If 12.7 Cardiac valve replacement / repair is equal to **Yes** answer this question:

12.7.1 **Aortic Valve replacement/repair***

- Yes
- No

Field type: Radiobutton
Option group: Yes/No_
Variable name: MH_AV_rep

If 12.7.1 Aortic Valve replacement/repair is equal to **Yes** answer this question:

12.7.1.1 **Type of aortic valve replacement/repair***

- AVR bio
- AVR mechano
- TAVI (intervention)
- TAVI (transapical)
- AV repair
- Other

Field type: Radiobutton
Option group: MH_AV_rep
Variable name: MH_AV_rep_type

If 12.7.1.1 Type of aortic valve replacement/repair is equal to **Other** answer this question:

12.7.1.1.1 **What other aortic valve replacement/repair?***

Field type: Textfield
Variable name: MH_AV_rep_type_other

If 12.7 Cardiac valve replacement / repair is equal to **Yes** answer this question:

12.7.2 **Mitral valve intervention***

- Yes
- No

Field type	Radiobutton	Variable name	MH_MV_int
Option group	Yes/No_		

If **12.7.2 Mitral valve intervention** is equal to **Yes** answer this question:

12.7.2.1 **Type of mitral valve intervention***

- ◯ MVR bio
- ◯ MVR mechano
- ◯ MVP
- ◯ Mitralclip
- ◯ Other

Field type	Radiobutton	Variable name	MH_MV_int_type
Option group	MH_MR_int_type		

If **12.7.2.1 Type of mitral valve intervention** is equal to **Other** answer this question:

12.7.2.1.1 **What other mitral valve intervention?***

Field type	Textfield	Variable name	MH_MV_int_type_other

If **12.7 Cardiac valve replacement / repair** is equal to **Yes** answer this question:

12.7.3 **Other replacement/repair type***

Field type	Multiline Textfield	Variable name	MH_VDR_type_other

If **12.7 Cardiac valve replacement / repair** is equal to **Yes** answer this question:

12.7.4 **Date of first valvular replacement***
If date is not exactly known, use the median date as an estimation (15th of the month if exact day is unknown, or July 1st when only the year is known). If completely unknown, use 01-01-1800.

DD MM YYYY

Error message shown if field's value is greater than **NOW**

No future dates can be entered

Field type	Date	Variable name	MH_VDR_d

Arrhythmia

12.8 **Atrial fibrillation***
noted in medical history (any type)

- Yes
- No

Field type	Radiobutton	Variable name	MH_SVT_AF
Option group	Yes/No_		

If **12.8 Atrial fibrillation** is equal to **Yes** answer this question:

12.8.1 **Atrial fibrillation type***
Choose most severe form that is applicable. Paroxysmal AF = < 7 days; Persistent AF = > 7 days; Permanent AF = >7 days and arrhytmia is accepted (not treated with anti-arrhytmic drugs or interventions, only heart rate control)

- paroxysmal AF
- persistent AF
- permanent AF
- non-specified AF

Field type	Radiobutton	Variable name	MH_SVT_AF_type
Option group	AF type		

If **12.8 Atrial fibrillation** is equal to **Yes** answer this question:

12.8.2 **Date first diagnosis AF (regardless of AF type)***
If date is not exactly known, use the median date as an estimation (15th of the month if exact day is unknown, or July 1st when only the year is known). If completely unknown, use 01-01-1800.

DD MM YYYY

Error message shown if field's value is greater than **NOW**

No future dates can be entered

Field type	Date	Variable name	MH_SVT_AF_d

If **12.8.1 Atrial fibrillation type** is not equal to **paroxysmal AF** answer this question:

12.8.1.1 **Date since current progressed type of AF***
Choose date at which the most progressed stage of AF was diagnosed.

DD MM YYYY

Error message shown if field's value is greater than **NOW**

No future dates can be entered

Field type	Date	Variable name	MH_SVT_AF_progression_d

12.9 **Atrial flutter***
noted in medical history

- Yes
- No

Field type	Radiobutton	Variable name	MH_SVT_AFL
Option group	Yes/No_		

If **12.9 Atrial flutter** is equal to **Yes** answer this question:

12.9.1 **Date first diagnosis Atrial Flutter***
If date is not exactly known, use the median date as an estimation (15th of the month if exact day is unknown, or July 1st when only the year is known). If completely unknown, use 01-01-1800.

DD MM YYYY

Error message shown if field's value is greater than **NOW**

No future dates can be entered

Field type	Date	Variable name	MH_SVT_AFL_d

12.10 **Supraventricular tachycardia (other than AF/Aflut)***
noted in medical history

- Yes
- No

Field type	Radiobutton	Variable name	MH_SVT_oth
Option group	Yes/No_		

If **12.10 Supraventricular tachycardia (other than AF/Aflut)** is equal to **Yes** answer this question:

12.10.1 **Type of SVT***

This radio field has too many options to list here. See Option Group - type of SVT for its options.

Field type	Radiobutton	Variable name	MH_SVT_other_type
Option group	type of SVT		

If **12.10.1 Type of SVT** is equal to **Other** answer this question:

12.10.1.1 **What other type of SVT?***

Field type Textfield

Variable name MH_SVT_other_type_other

If 12.10 **Supraventricular tachycardia (other than AF/Aflut)** is equal to **Yes** answer this question:

12.10.2 **Date first diagnosis supraventricular tachycardia (other than AF/Aflut)***
If date is not exactly known, use the median date as an estimation (15th of the month if exact day is unknown, or July 1st when only the year is known). If completely unknown, use 01-01-1800.

DD MM YYYY

Error message shown if field's value is greater than **NOW**

No future dates can be entered

Field type Date

Variable name MH_SVT_oth_d

If 12.11 **Calculation SVT** is equal to **1** answer this question:

12.11.1 **SVT intervention: ablation/maze***

- Yes
- No

Field type Radiobutton
Option group Yes/No_

Variable name MH_SVT_int_abl

If 12.11.1 **SVT intervention: ablation/maze** is equal to **Yes** answer this question:

12.11.1.1 **Date first ablation/maze***
If date is not exactly known, use the median date as an estimation (15th of the month if exact day is unknown, or July 1st when only the year is known). If completely unknown, use 01-01-1800.

DD MM YYYY

Error message shown if field's value is greater than **NOW**

No future dates can be entered

Field type Date

Variable name MH_SVT_int_abl_d

If **12.11 Calculation SVT** is equal to **1** answer this question:

12.11.2 **Cardioversion for SVT (ECV/Chemical)***

- Yes
- No

Field type Radiobutton
Option group Yes/No_

Variable name MH_SVT_int_ecv

If 12.11.2 Cardioversion for SVT (ECV/Chemical) is equal to **Yes** answer this question:

12.11.2.1 **Date first cardioversion***
If date is not exactly known, use the median date as an estimation (15th of the month if exact day is unknown, or July 1st when only the year is known). If completely unknown, use 01-01-1800.

DD MM YYYY

Error message shown if field's value is greater than **NOW**

No future dates can be entered

Field type Date

Variable name MH_SVT_int_ecv_d

12.12 **Ventricular arrhythmia***

- Yes
- No

Field type Radiobutton
Option group Yes/No_
Variable name MH_VT

If **12.12 Ventricular arrhytmia** is equal to **Yes** answer this question:

12.12.1 **Type of ventricular arrhytmia***

- non-sustained VT
- sustained VT
- VF
- PVCs
- Other

Field type Radiobutton
Option group MH_VT_type
Variable name MH_VT_type

If **12.12.1 Type of ventricular arrhytmia** is equal to **Other** answer this question:

12.12.1.1 **What other type of Ventricular Arrhythmia?***

Field type Textfield
Variable name MH_VT_other_type

If **12.12 Ventricular arrhytmia** is equal to **Yes** answer this question:

12.12.2 **Ventricular arrhytmia intervention: ablation***

- Yes
- No

Field type Radiobutton
Option group Yes/No_
Variable name MH_VT_int

If **12.12.2 Ventricular arrhytmia intervention: ablation** is equal to **Yes** answer this question:

12.12.2.1 **Date of first ablaltion***
If date is not exactly known, use the median date as an estimation (15th of the month if exact day is unknown, or July 1st when only the year is known). If completely unknown, use 01-01-1800.

DD MM YYYY

Error message shown if field's value is greater than **NOW**

No future dates can be entered

Field type	Date	Variable name	MH_VT_int_d

(surgical) interventions

12.13 **Myectomy***

- Yes
- No

Field type	Radiobutton	Variable name	MH_myectomy
Option group	Yes/no		

If **12.13 Myectomy** is equal to **Yes** answer this question:

12.13.1 **Date of first myectomy***
If date is not exactly known, use the median date as an estimation (15th of the month if exact day is unknown, or July 1st when only the year is known). If completely unknown, use 01-01-1800.

DD MM YYYY

Error message shown if field's value is greater than **NOW**

No future dates can be entered

Field type	Date	Variable name	MH_myectomy_d

12.14 **Alcohol septum ablation**

- ◯ Yes
- ◯ No

Field type: Radiobutton
Option group: Yes/no
Variable name: MH_alcohol_septum_abl

If **12.14 Alcohol septum ablation** is equal to **Yes** answer this question:

12.14.1 **Date of first alcohol septum ablation**
If date is not exactly known, use the median date as an estimation (15th of the month if exact day is unknown, or July 1st when only the year is known). If completely unknown, use 01-01-1800.

DD MM YYYY

Error message shown if field's value is greater than **NOW**

No future dates can be entered

Field type: Date
Variable name: MH_alcohol_septum_abl_d

12.15 Additional remarks

Field type: Multiline Textfield
Variable name: MH_car_remarks

Medical Events/history & Family history

13. Non Cardiac History/Events

Non-cardiac medical history

13.1 **Date medical information checked***
essential for future analyses, as it represents the most recent point up to which information is available

DD MM YYYY

Error message shown if field's value is greater than **NOW**

this cannot be a future date!

Field type Date

Variable name MH_nonc_upd_d

13.2 Date check

Calculated value

Error message shown if field's value is equal to **Error**

Date information checked must be later than date first presentation at this hospital

Field type Calculation

Variable name MH_nonc_upd_d_check

Vascular diseases

13.3 **Transient ischemic attack (TIA)***
A brief episode of neurological dysfunction caused by loss of blood flow (ischemia) in the brain, spinal cord, or retina, without tissue death (infarction). This includes for example amaurosis fugax.

- Yes
- No

Field type Radiobutton
Option group Yes/No__
Variable name MH_TIA

If **13.3 Transient ischemic attack (TIA)** is equal to **Yes** answer this question:

13.3.1 **Date (first) TIA***
If date is not exactly known, use the median date as an estimation (15th of the month if exact day is unknown, or July 1st when only the year is known). If completely unknown, use 01-01-1800.

DD MM YYYY

Error message shown if field's value is greater than **NOW**

No future dates can be entered

Field type Date
Variable name MH_TIA_d

13.4 **Stroke***
Permanent neurological dysfunction due to focal ischemia of brain, spine or retina, caused by an acute infraction of neurological tissue due to trombosis, embolus, systemic hypoperfusion or bleeding.

- Yes
- No

Field type Radiobutton
Option group Yes/No__
Variable name MH_stroke

If **13.4 Stroke** is equal to **Yes** answer this question:

13.4.1 **Date (first) stroke***
If date is not exactly known, use the median date as an estimation (15th of the month if exact day is unknown, or July 1st when only the year is known). If completely unknown, use 01-01-1800.

DD MM YYYY

Error message shown if field's value is greater than **NOW**

No future dates can be entered

Field type Date Variable name MH_stroke_d

13.5 **Peripheral artery disease**
Perhiphera artery disease is defined as one of the following: • Claudicatio intermittens • Carotis stenosis of > 50% • Limb amputation due to artery disease • Previous or expected surgical operation of the abdominal aorta, arteries of the limb or carotid artery.

- Yes
- No

Field type Radiobutton
Option group Yes/No_
Variable name MH_PAD

If **13.5 Peripheral artery disease** is equal to **Yes** answer this question:

13.5.1 **Date first diagnosis peripheral artery disease**
If date is not exactly known, use the median date as an estimation (15th of the month if exact day is unknown, or July 1st when only the year is known). If completely unknown, use 01-01-1800.

DD MM YYYY

Error message shown if field's value is greater than **NOW**

No future dates can be entered

Field type Date Variable name MH_PAD_d

Pulmonary diseases

13.6 **Pulmonary hypertension***
Noted in medical history. Please check if according to ESC guidelines pulmonary hypertension 2015: http://eurheartj.oxfordjournals.org/lookup/doi/10.1093/eurheartj/ehv317. If noted in medical history but not according to guidelines, choose yes and use comment on the right to elaborate.

- ◯ Yes
- ◯ No

Field type Radiobutton
Option group Yes/No_
Variable name MH_PHT

If 13.6 **Pulmonary hypertension** is equal to **Yes** answer this question:

13.6.1 **First diagnosis of pulmonary hypertension***
If date is not exactly known, use the median date as an estimation (15th of the month if exact day is unknown, or July 1st when only the year is known). If completely unknown, use 01-01-1800.

DD MM YYYY

Error message shown if field's value is greater than **NOW**

No future dates can be entered

Field type Date
Variable name MH_PHT_d

If 13.6 **Pulmonary hypertension** is equal to **Yes** answer this question:

13.6.2 **Pulmonary hypertension classification***
Based on ESC guidelines pulmonary hypertension 2015: http://eurheartj.oxfordjournals.org/lookup/doi/10.1093/eurheartj/ehv317

- ◯ pulmonary arterial hypertension (group 1)
- ◯ PH due to left heart disease (group 2)
- ◯ PH due to pulmonary pathology and/or hypoxia (group 3)
- ◯ Chronic trombo-embolic PH (CTEPH) (group 4)
- ◯ Multifactorial/mixed PH (group 5)
- ◯ Unknown type

Field type: Radiobutton
Option group: Pulmonary hypertension
Variable name: MH_PHT_group

13.7 **Chronic obstructive pulmonary disease (COPD)***
Noted in medical history. Actively look at letters from pulmonologist and general physician. Watch out for use of pulmonology medication.

- ○ Yes
- ○ No

Field type: Radiobutton
Option group: Yes/No
Variable name: MH_COPD

If **13.7 Chronic obstructive pulmonary disease (COPD)** is equal to **Yes** answer this question:

13.7.1 **COPD gold classification***

- ○ COPD gold 1
- ○ COPD gold 2
- ○ COPD gold 3
- ○ COPD gold 4
- ○ COPD stage unknown

Field type: Radiobutton
Option group: COPD stage
Variable name: MH_COPD_t

If **13.7 Chronic obstructive pulmonary disease (COPD)** is equal to **Yes** answer this question:

13.7.2 **Date first diagnosis of COPD***
If date is not exactly known, use the median date as an estimation (15th of the month if exact day is unknown, or July 1st when only the year is known). If completely unknown, use 01-01-1800.

DD MM YYYY

Error message shown if field's value is greater than **NOW**

No future dates can be entered

Field type	Date	Variable name	MH_COPD_d

13.8 **Asthma***
Noted in medical history. Actively look at letters from pulmonologist and general physician. Watch out for use of pulmonology medication.

- Yes
- No

Field type	Radiobutton	Variable name	MH_Asthma
Option group	Yes/No_		

If **13.8 Asthma** is equal to **Yes** answer this question:

13.8.1 **Date first diagnosis of Asthma***
If date is not exactly known, use the median date as an estimation (15th of the month if exact day is unknown, or July 1st when only the year is known). If completely unknown, use 01-01-1800.

DD MM YYYY

Field type	Date	Variable name	MH_Asthma_d

13.9 **Other pulmonary disease (emphysema, ILD)***
This includes emphysema, interstitial lung disease (ILD)

- Yes
- No

Field type Radiobutton
Option group Yes/No
Variable name MH_PD_oth

If **13.9 Other pulmonary disease (emphysema, ILD)** is equal to **Yes** answer this question:

13.9.1 **Date first diagnosis of other pulmonary disease***
If date is not exactly known, use the median date as an estimation (15th of the month if exact day is unknown, or July 1st when only the year is known). If completely unknown, use 01-01-1800.

DD MM YYYY

Error message shown if field's value is greater than **NOW**

No future dates can be entered

Field type Date
Variable name MH_PD_oth_d

13.10 **Pulmonary embolus***

- Yes
- No

Field type Radiobutton
Option group Yes/No__
Variable name MH_PE

If **13.10 Pulmonary embolus** is equal to **Yes** answer this question:

13.10.1 **Date of first pulmonary embolus***
If date is not exactly known, use the median date as an estimation (15th of the month if exact day is unknown, or July 1st when only the year is known). If completely unknown, use 01-01-1800.

DD MM YYYY

Error message shown if field's value is greater than **NOW**

No future dates can be entered

Field type Date Variable name MH_PE_d

13.11 **Sleep apnea / sleep breathing disorder***

- Yes
- No

Field type Radiobutton Variable name MH_sleepapnea
Option group Yes/No_

If 13.11 Sleep apnea / sleep breathing disorder is equal to **Yes** answer this question:

13.11.1 **Sleep apnea type***

- OSAS
- CSAS
- Both OSAS and CSAS
- Unknown

Field type Radiobutton Variable name MH_sleepapnea_type
Option group Sleep apnea

If 13.11 Sleep apnea / sleep breathing disorder is equal to **Yes** answer this question:

13.11.2 **Date first diagnosis of sleep apnea***
If date is not exactly known, use the median date as an estimation (15th of the month if exact day is unknown, or July 1st when only the year is known). If completely unknown, use 01-01-1800.

DD MM YYYY

Error message shown if field's value is greater than **NOW**

No future dates can be entered

Field type	Date	Variable name	MH_sleepapnea_d

If 13.11 **Sleep apnea / sleep breathing disorder** is equal to **Yes** answer this question:

13.11.3 **Sleep apnea intervention***
Select applicable therapy. If patient is prescribed a therapy but has never used it after prescription/refused to use it, please select none. Still, patients could receive other therapy such as weight reduction.

- ☐ none
- ☐ CPAP
- ☐ mandibular reposition device
- ☐ sleep position therapy
- ☐ surgery
- ☐ weight reduction
- ☐ other
- ☐ unknown

Field type	Checkbox	Variable name	MH_sleepapnea_int
Option group	Sleep apnea intervention		

If 13.11.3 **Sleep apnea intervention** is checked on **other** answer this question:

13.11.3.1 Specify other sleep intervention device

Field type	Textfield	Variable name	MH_sleepapnea_int_other

Endocrine diseases

13.12 **Diabetes Mellitus***
Noted in medical history. Actively look for use of antidiabetic medication and letters of general practitioner.

- ○ Yes
- ○ No

Field type Radiobutton
Option group Yes/No_
Variable name MH_DM

If **13.12 Diabetes Mellitus** is equal to **Yes** answer this question:

13.1 2.1 **Diabetes Mellitus type***

- Type 1 DM
- Type 2 DM
- Diabetes Insipidus
- other Type DM
- Unknown

Field type Radiobutton
Option group Diabetes mellitus types
Variable name MH_DM_type

If **13.12 Diabetes Mellitus** is equal to **Yes** answer this question:

13.1 2.2 **Diabetes treatment***
When multiple options are possible. Please select one treatment (strongest) in the following order: insuline > oral medication > diet > no treatment.

- No treatment
- Diet
- Oral medication
- Insulin
- Other
- Treatment unknown

Field type Radiobutton
Option group DM treatment
Variable name MH_DM_rx

If **13.12 Diabetes Mellitus** is equal to **Yes** answer this question:

13.1 2.3 **Date first diagnosis diabetes mellitus***
If date is not exactly known, use the median date as an estimation (15th of the month if exact day is unknown, or July 1st when only the year is known). If completely unknown, use 01-01-1800.

DD MM YYYY

Error message shown if field's value is greater than **NOW**

No future dates can be entered

Field type Date

Variable name MH_DM_d

13.13 **Kidney disease***
based on medical history or GFR <60 during more than 3 months (at least 2 or more measurements)

- ○ Yes
- ○ No

Field type Radiobutton
Option group Yes/No_

Variable name MH_KD

If **13.13 Kidney disease** is equal to **Yes** answer this question:

13.13.1 **Date first diagnosis kidney disease***
If date is not exactly known, use the median date as an estimation (15th of the month if exact day is unknown, or July 1st when only the year is known). If completely unknown, use 01-01-1800.

DD MM YYYY

Error message shown if field's value is greater than **NOW**

No future dates can be entered

Field type Date

Variable name MH_KD_d

If **13.13 Kidney disease** is equal to **Yes** answer this question:

13.13.2 **Dialysis***

- ○ Yes
- ○ No

Field type Radiobutton
Option group Yes/No__
Variable name MH_KD_dial

If **13.13.2 Dialysis** is equal to **Yes** answer this question:

13.13.2.1 **Date first dialysis***
If date is not exactly known, use the median date as an estimation (15th of the month if exact day is unknown, or July 1st when only the year is known). If completely unknown, use 01-01-1800.

DD MM YYYY

Error message shown if field's value is greater than **NOW**

No future dates can be entered

Field type Date
Variable name MH_KD_dial_d

If **13.13 Kidney disease** is equal to **Yes** answer this question:

13.13.3 **Kidney transplantation***

- Yes
- No

Field type Radiobutton
Option group Yes/No_
Variable name MH_KD_ntx

If **13.13.3 Kidney transplantation** is equal to **Yes** answer this question:

13.13.3.1 **Date of kidney transplantation***
If date is not exactly known, use the median date as an estimation (15th of the month if exact day is unknown, or July 1st when only the year is known). If completely unknown, use 01-01-1800.

DD MM YYYY

Error message shown if field's value is greater than **NOW**

No future dates can be entered

Field type Date

Variable name MH_KD_ntx_d

13.14 **Thyreoid disease***
Noted in medical history. Watch out for use of thyroid medication such as euthyrox. Subclinical hyper/hypothyreoidism should be noted as 'no'. HyperPARAthyreoidism is not a thyroid disease.

- Yes
- No

Field type Radiobutton
Option group Yes/No__

Variable name MH_Thy

If 13.14 **Thyreoid disease** is equal to **Yes** answer this question:

13.14.1 **Type of thyreoid disease***
if multiple take first diagnosis

- HYPERthyreoidism
- HYPOthyreoidism
- other
- unknown

Field type Radiobutton
Option group Thyroid disease

Variable name MH_Thy_type

If 13.14.1 **Type of thyreoid disease** is equal to **other** answer this question:

13.14.1.1 **type of thyreoid disease***

Field type Multiline Textfield

Variable name MH_Thy_type_other

If 13.14 **Thyroid disease** is equal to **Yes** answer this question:

13.14.2 **Date first diagnosis thyreoid disease***
If date is not exactly known, use the median date as an estimation (15th of the month if exact day is unknown, or July 1st when only the year is known). If completely unknown, use 01-01-1800.

DD MM YYYY

Error message shown if field's value is greater than **NOW**

No future dates can be entered

Field type Date

Variable name MH_Thy_d

13.15 **Anaemia***
in medical history. Anaemia as a consequence of surgery is NOT considered anaemia.

- Yes
- No

Field type Radiobutton
Option group Yes/No_

Variable name MH_anaemia

If 13.15 **Anaemia** is equal to **Yes** answer this question:

13.15.1 **Date first diagnosis anaemia***
If date is not exactly known, use the median date as an estimation (15th of the month if exact day is unknown, or July 1st when only the year is known). If completely unknown, use 01-01-1800.

DD MM YYYY

Error message shown if field's value is greater than **NOW**

No future dates can be entered

Field type Date

Variable name MH_anaemia_d

13.16 **Iron deficiency***
Noted in medical history

- Yes
- No

Field type Radiobutton
Option group Yes/No_

Variable name MH_FeD

If 13.16 Iron deficiency is equal to **Yes** answer this question:

13.16.1 **Date first diagnosis iron deficiency***
If date is not exactly known, use the median date as an estimation (15th of the month if exact day is unknown, or July 1st when only the year is known). If completely unknown, use 01-01-1800.

DD MM YYYY

Error message shown if field's value is greater than **NOW**

No future dates can be entered

Field type Date

Variable name MH_FeD_d

Malignant diseases

13.17 **Cancer (current or previous)***
Any form of cancer except basal cell carcinoma.

- Yes
- No

Field type Radiobutton
Option group Yes/No_
Variable name MH_Can

⊰ If **13.17 Cancer (current or previous)** is equal to **Yes** answer this question:

13.17.1 **Is cancer still present?***

- Yes
- No

Field type Radiobutton
Option group Yes/No_
Variable name MH_Can_current

⊰ If **13.17 Cancer (current or previous)** is equal to **Yes** answer this question:

13.17.2 **Type of cancer***
Only primary cancer, no metastasis.

This checkbox field has too many options to list here. See Option Group - Type cancer for its options.

Field type Checkbox
Option group Type cancer
Variable name MH_Can_type

⊰ If **13.17.2 Type of cancer** is checked on **Other** answer this question:

13.17.2.1 **type of cancer***

Field type Multiline Textfield
Variable name MH_Can_type_other

⊰ If **13.17 Cancer (current or previous)** is equal to **Yes** answer this question:

13.17.3 **First diagnosis cancer***
If date is not exactly known, use the median date as an estimation (15th of the month if exact day is unknown, or July 1st when only the year is known). If completely unknown, use 01-01-1800.

DD MM YYYY

Error message shown if field's value is greater than **NOW**

No future dates can be entered

Field type	Date	Variable name	MH_Can_d

If 13.17 **Cancer (current or previous)** is equal to **Yes** answer this question:

13.17.4 **Underwent chest radiation***

- Yes
- No

Field type	Radiobutton	Variable name	MH_Can_CHR
Option group	Yes/No_		

If 13.17.4 **Underwent chest radiation** is equal to **Yes** answer this question:

13.17.4.1 **Date of start chest-radiation***
If date is not exactly known, use the median date as an estimation (15th of the month if exact day is unknown, or July 1st when only the year is known). If completely unknown, use 01-01-1800.

DD MM YYYY

Error message shown if field's value is greater than **NOW**

No future dates can be entered

Field type	Date	Variable name	MH_Can_CHR_d

If **13.17 Cancer (current or previous)** is equal to **Yes** answer this question:

13.17.5 **Received chemotherapy***

- Yes
- No

Field type: Radiobutton
Option group: Yes/No_
Variable name: MH_Can_chemo

If date is not exactly known, use the median date as an estimation (15th of the month if exact day is unknown, or July 1st when only the year is known). If completely unknown, use 01-01-1800.

Other diseases / risk factors

13.18 **Hypertension***
noted in medical history

- Yes
- No

Field type: Radiobutton
Option group: Yes/No_
Variable name: MH_HT

If **13.18 Hypertension** is equal to **Yes** answer this question:

13.18.1 **Date of first diagnosis hypertension***
If date is not exactly known, use the median date as an estimation (15th of the month if exact day is unknown, or July 1st when only the year is known). If completely unknown, use 01-01-1800.

DD MM YYYY

Error message shown if field's value is greater than **NOW**

No future dates can be entered

Field type: Date
Variable name: MH_HT_d

13.19 **Hypercholesterolemia***
noted in medical history

- Yes
- No

Field type: Radiobutton
Option group: Yes/No_
Variable name: MH_HC

If 13.19 **Hypercholesterolemia** is equal to **Yes** answer this question:

13.19.1 **Date of first diagnosis hypercholesterolemia***
If date is not exactly known, use the median date as an estimation (15th of the month if exact day is unknown, or July 1st when only the year is known). If completely unknown, use 01-01-1800.

DD MM YYYY

Error message shown if field's value is greater than **NOW**

No future dates can be entered

Field type: Date
Variable name: MH_HC_d

13.20 **Auto-immune/-inflammatory disease***
(Rheumatoid) arthritis; Gout; Morbus Bechterew; Psoriasis; Crohn's disease; Celiac disease; Systemic lupus erythematodes (SLE); Sjogren's syndrome; Vasculitis;for a complete overview of auto-immune/-inflammatory diseases: doi:10.1093/eurheartj/ehx321

- Yes
- No

Field type: Radiobutton
Option group: Yes/No_
Variable name: MH_SD_auto

If 13.20 **Auto-immune/-inflammatory disease** is equal to **Yes** answer this question:

13.20.1 **Type auto-immune/-inflammatory disease(s)***

This checkbox field has too many options to list here. See Option Group - auto-immune diseases for its options.

Field type	Checkbox	Variable name	MH_SD_auto_type
Option group	auto-immune diseases		

If 13.20.1 **Type auto-immune/-inflammatory disease(s)** is checked on **Other** answer this question:

13.20.1.1 **Other auto-immune/auto-inflammatory disease(s)***

Field type	Multiline Textfield	Variable name	MH_SD_auto_type_other

If 13.20 **Auto-immune/-inflammatory disease** is equal to **Yes** answer this question:

13.20.2 **Date first diagnosis auto-immune/-inflammatory disease(s)***
If date is not exactly known, use the median date as an estimation (15th of the month if exact day is unknown, or July 1st when only the year is known). If completely unknown, use 01-01-1800.

DD MM YYYY

Error message shown if field's value is greater than **NOW**

No future dates can be entered

Field type	Date	Variable name	MH_SD_auto_d

13.21 **Carpal tunnel syndrome (CTS)***

- Yes
- No

Field type	Radiobutton	Variable name	MH_CTS
Option group	Yes/No		

If 13.21 **Carpal tunnel syndrome (CTS)** is equal to **Yes** answer this question:

13.21.1 **CTS location***

- Unilateral
- Bilateral
- Unspecified
- Unknown

Field type	Radiobutton	Variable name	MH_CTS_loc
Option group	Location		

If 13.21 **Carpal tunnel syndrome (CTS)** is equal to **Yes** answer this question:

13.21.2 **Date first diagnosis CTS***
If date is not exactly known, use the median date as an estimation (15th of the month if exact day is unknown, or July 1st when only the year is known). If completely unknown, use 01-01-1800.

DD MM YYYY

Error message shown if field's value is greater than **NOW**

No future dates can be entered

Field type	Date	Variable name	MH_CTS_d

13.22 **Polyneuropathy (PNP)***

- ○ Yes
- ○ No

Field type: Radiobutton
Option group: Yes/No
Variable name: MH_PNP

If **13.22 Polyneuropathy (PNP)** is equal to **Yes** answer this question:

13.22.1 **Date first diagnosis PNP***
If date is not exactly known, use the median date as an estimation (15th of the month if exact day is unknown, or July 1st when only the year is known). If completely unknown, use 01-01-1800.

DD MM YYYY

Error message shown if field's value is greater than **NOW**

No future dates can be entered

Field type: Date
Variable name: MH_PNP_d

13.25 **Neuromuscular disease***
proven or high suspicion

- ○ Yes
- ○ No
- ○ Unknown

Field type: Radiobutton
Option group: Yes/No/Unknown
Variable name: MH_NM

If **13.25 Neuromuscular disease** is equal to **Yes** answer this question:

13.25.1 **What Neuromuscular disease?***

Field type Textfield Variable name MH_NM_type

If **13.25 Neuromuscular disease** is equal to **Yes** answer this question:

13.25.2 **Date first diagnosis Neuromuscular disease***
If date is not exactly known, use the median date as an estimation (15th of the month if exact day is unknown, or July 1st when only the year is known). If completely unknown, use 01-01-1800.

DD MM YYYY

Error message shown if field's value is greater than **NOW**

No future dates can be entered

Field type Date Variable name MH_NM_d

13.26 Additional remarks

Field type Multiline Textfield Variable name MH_noncar_remarks

Medical Events/history & Family history

14. Hospitalisation (incl Cardiac Arrest /LTA/LVAD/HTx)

14.1 **Date form last updated***

DD MM YYYY

ⓘ **Warning** message shown if field's value is smaller than **NOW**

Please update this form until today and subsequently fill in today's date in the field above

❗ **Error** message shown if field's value is greater than **NOW**

No future dates can be entered

Field type	Date	Variable name	Hosp_up_d

14.2 Date check

Calculated value

❗ **Error** message shown if field's value is equal to **Error**

Date form last updated must be later than date first presentation at this hosptial

Field type	Calculation	Variable name	Hosp_up_d_check

14.3 **Heart transplantation***

- ○ Yes
- ○ No

Field type<br>Option group	Radiobutton<br>Yes/No	Variable name	HTx

If **14.3 Heart transplantation** is equal to **Yes** answer this question:

14.3.1 **Heart transplantation date**
If date is not exactly known, use the median date as an estimation (15th of the month if exact day is unknown, or July 1st when only the year is known). If completely unknown, use 01-01-1800.

DD MM YYYY

Field type: Date
Variable name: HTx_d

14.4 **Ventricular assist device**

- Yes
- No

Field type: Radiobutton
Option group: Yes/No
Variable name: VAD

If **14.4 Ventricular assist device** is equal to **Yes** answer this question:

14.4.1 **VAD date**
If date is not exactly known, use the median date as an estimation (15th of the month if exact day is unknown, or July 1st when only the year is known). If completely unknown, use 01-01-1800.

DD MM YYYY

Error message shown if field's value is greater than **NOW**

No future dates can be entered

Field type: Date
Variable name: VAD_d

14.5 **Hospitalisation**

- Yes
- No

Field type Radiobutton
Option group Yes/No
Variable name Hosp_yn

If **14.5 Hospitalisation** is equal to **Yes**:

14.5.1 Hospitalisation information (**please don't include elective hospitalisations for additional diagnostics!**)
(linked Repeating Data Form: "Hospitalization")

Created on	Admission date (see info in case of referred admission)	Discharge date	Date check	Admitted at cardiology department	Cardiology consultation during hospitalisation	Reason for hospitalization

Field type Repeated measures

Medical Events/history & Family history

15. Devices & device therapy

15.1 **Last Updated***

DD MM YYYY

Warning message shown if field's value is smaller than **NOW**

Please update this form until today and subsequently fill in today's date in the field above

Field type: Date

Variable name: Dev_up_d

15.2 Date check

Calculated value

Error message shown if field's value is equal to **Error**

Date form last updated must be later than date first presentation at this hospital

Field type: Calculation

Variable name: Dev_up_d_check

15.3 **Device implantation (ICD/PM)***
ILR/reveal is not considered a cardiac device

- Yes
- No

Field type: Radiobutton

Option group: Yes/No

Variable name: Dev_yn

If **15.3 Device implantation (ICD/PM)** is equal to **Yes** answer this question:

15.3.1 Device implantation(s)

	Type of device	Reason implantation	Date of implantation	Remarks
First implantation	○ Pacemaker- VVI<br>○ Pacemaker- DDD<br>○ CRT-P<br>○ CRT-D<br>○ ICD -VVI<br>○ ICD- DDD<br>○ ICD<br>○ Yes, type unknown	○ primary prevention<br>○ secondary prevention<br>○ battery end of life<br>○ removal of device without new implantation (or device turned off)<br>○ other		
Implantation 2	○ Pacemaker- VVI<br>○ Pacemaker- DDD<br>○ CRT-P<br>○ CRT-D<br>○ ICD -VVI<br>○ ICD- DDD<br>○ ICD<br>○ Yes, type unknown	○ primary prevention<br>○ secondary prevention<br>○ battery end of life<br>○ removal of device without new implantation (or device turned off)<br>○ other		
Implantation 3	○ Pacemaker- VVI<br>○ Pacemaker- DDD<br>○ CRT-P<br>○ CRT-D<br>○ ICD -VVI<br>○ ICD- DDD<br>○ ICD<br>○ Yes, type unknown	○ primary prevention<br>○ secondary prevention<br>○ battery end of life<br>○ removal of device without new implantation (or device turned off)<br>○ other		
Implantation 4	○ Pacemaker- VVI<br>○ Pacemaker- DDD<br>○ CRT-P<br>○ CRT-D<br>○ ICD -VVI<br>○ ICD- DDD<br>○ ICD<br>○ Yes, type unknown	○ primary prevention<br>○ secondary prevention<br>○ battery end of life<br>○ removal of device without new implantation (or device turned off)<br>○ other		

Implantation 5	◯ Pacemaker- VVI<br>◯ Pacemaker- DDD<br>◯ CRT-P<br>◯ CRT-D<br>◯ ICD -VVI<br>◯ ICD- DDD<br>◯ ICD<br>◯ Yes, type unknown	◯ primary prevention<br>◯ secondary prevention<br>◯ battery end of life<br>◯ removal of device without new implantation (or device turned off)<br>◯ other		
Implantation 6	◯ Pacemaker- VVI<br>◯ Pacemaker- DDD<br>◯ CRT-P<br>◯ CRT-D<br>◯ ICD -VVI<br>◯ ICD- DDD<br>◯ ICD<br>◯ Yes, type unknown	◯ primary prevention<br>◯ secondary prevention<br>◯ battery end of life<br>◯ removal of device without new implantation (or device turned off)<br>◯ other		
Implantation 7	◯ Pacemaker- VVI<br>◯ Pacemaker- DDD<br>◯ CRT-P<br>◯ CRT-D<br>◯ ICD -VVI<br>◯ ICD- DDD<br>◯ ICD<br>◯ Yes, type unknown	◯ primary prevention<br>◯ secondary prevention<br>◯ battery end of life<br>◯ removal of device without new implantation (or device turned off)<br>◯ other		
Implantation 8	◯ Pacemaker- VVI<br>◯ Pacemaker- DDD<br>◯ CRT-P<br>◯ CRT-D<br>◯ ICD -VVI<br>◯ ICD- DDD<br>◯ ICD<br>◯ Yes, type unknown	◯ primary prevention<br>◯ secondary prevention<br>◯ battery end of life<br>◯ removal of device without new implantation (or device turned off)<br>◯ other		

Implantation 9	◯ Pacemaker- VVI<br>◯ Pacemaker- DDD<br>◯ CRT-P<br>◯ CRT-D<br>◯ ICD -VVI<br>◯ ICD- DDD<br>◯ ICD<br>◯ Yes, type unknown	◯ primary prevention<br>◯ secondary prevention<br>◯ battery end of life<br>◯ removal of device without new implantation (or device turned off)<br>◯ other		
Implantation 10	◯ Pacemaker- VVI<br>◯ Pacemaker- DDD<br>◯ CRT-P<br>◯ CRT-D<br>◯ ICD -VVI<br>◯ ICD- DDD<br>◯ ICD<br>◯ Yes, type unknown	◯ primary prevention<br>◯ secondary prevention<br>◯ battery end of life<br>◯ removal of device without new implantation (or device turned off)<br>◯ other		

Field type Grid

Variable name Dev_d

⊰ If **15.3 Device implantation (ICD/PM)** is equal to **Yes** answer this question:

15.3.2 Received (un)justified shock(s)/anti-tachypacing (ATP)

○ Yes

○ No

Field type: Radiobutton
Option group: Yes/No
Variable name: Dev_shocks_ATP_yn

⊰ If **15.3.2 Received (un)justified shock(s)/anti-tachypacin...** is equal to **Yes**:

15.3.2 Add ICD readout
(linked Repeating Data Form: "ICD readout")

Date ICD readout	ICD therapy given?	ATP(s)?	ICD shock(s)?

Field type: Repeated measures

If 15.3.2 **Received (un)justified shock(s)/anti-tachypacin...** is equal to **Yes** answer this question:

15.3.2.2 Shocks/ATP

	Type	Date event	Date read out device	Remarks
event 1	○ Anti tachy Pacing (ATP)<br>○ Justified shock<br>○ Unjustified shock			
event 2	○ Anti tachy Pacing (ATP)<br>○ Justified shock<br>○ Unjustified shock			
event 3	○ Anti tachy Pacing (ATP)<br>○ Justified shock<br>○ Unjustified shock			
event 4	○ Anti tachy Pacing (ATP)<br>○ Justified shock<br>○ Unjustified shock			
event 5	○ Anti tachy Pacing (ATP)<br>○ Justified shock<br>○ Unjustified shock			
event 6	○ Anti tachy Pacing (ATP)<br>○ Justified shock<br>○ Unjustified shock			
event 7	○ Anti tachy Pacing (ATP)<br>○ Justified shock<br>○ Unjustified shock			
event 8	○ Anti tachy Pacing (ATP)<br>○ Justified shock<br>○ Unjustified shock			
event 9	○ Anti tachy Pacing (ATP)<br>○ Justified shock<br>○ Unjustified shock			
event 10	○ Anti tachy Pacing (ATP)<br>○ Justified shock<br>○ Unjustified shock			
event 11	○ Anti tachy Pacing (ATP)<br>○ Justified shock<br>○ Unjustified shock			

event 12	◯ Anti tachy Pacing (ATP)<br>◯ Justified shock<br>◯ Unjustified shock			
event 13	◯ Anti tachy Pacing (ATP)<br>◯ Justified shock<br>◯ Unjustified shock			
event 14	◯ Anti tachy Pacing (ATP)<br>◯ Justified shock<br>◯ Unjustified shock			
event 15	◯ Anti tachy Pacing (ATP)<br>◯ Justified shock<br>◯ Unjustified shock			

Field type Grid

Variable name Dev_shocks_ATP_d

If **15.3 Device implantation (ICD/PM)** is equal to **Yes** answer this question:

15.3.3 **Is the patient after device implantation referred to another center (for device follow- up)***

○ Yes
○ No

Field type Radiobutton
Option group Yes/No
Variable name Dev_referral

If **15.3.3 Is the patient after device implantation referr...** is equal to **Yes** answer this question:

15.3.3.1 **Date of referral***
If date is not exactly known, use the median date as an estimation (15th of the month if exact day is unknown, or July 1st when only the year is known). If completely unknown, use 01-01-1800.

DD MM YYYY

Field type Date
Variable name Dev_referral_d

Medical Events/history & Family history

16. (Loss to) follow-up

Patients are **not** loss to follow-up if they are still seen by **any** specialist in this hospital, or when they are living in the neighborhood

16.1 **Last Updated***

DD MM YYYY

Information message shown if field's value is greater than **1800-01-01**

Indien een patient terugverwezen is naar de huisarts en niet door een ander specialismen opgevolgd wordt dan is de patient LOF indien hij **NIET** uit de directe omgeving van dit ziekenhuis afkomstig is

Warning message shown if field's value is smaller than **NOW**

Please update this form until today and subsequently fill in today's date in the field above

Error message shown if field's value is greater than **NOW**

No future dates can be entered

Field type Date

Variable name LOF_up_d

16.2 Date check

Calculated value

Error message shown if field's value is equal to **Error**

Date form last updated must be later than date first presentation at this hospital

Field type Calculation

Variable name LOF_up_d_check

16.3 **Loss to follow-up***

- ○ Yes
- ○ No

Field type: Radiobutton
Option group: Yes/No
Variable name: LOF_yn

If **16.3 Loss to follow-up** is equal to **Yes** answer this question:

16.3.1 **Date Loss to follow-up***
If date is not exactly known, use the median date as an estimation (15th of the month if exact day is unknown, or July 1st when only the year is known). If completely unknown, use 01-01-1800.

DD MM YYYY

Error message shown if field's value is greater than **NOW**

No future dates can be entered

Field type: Date
Variable name: LOF_d

If **16.3 Loss to follow-up** is equal to **Yes** answer this question:

16.3.2 **Location for further follow-up***
If the location for further follow-up is both 2nd and 3rd line center, score the appropriate line that the patient is referred back for.

- ○ Is and stays in 2nd line, heart failure clinic
- ○ Is and stays in 3rd line, heart failure clinic
- ○ Return to general practitioner (1st line)
- ○ Goes to general cardiology outpatient
- ○ No follow-up, subject died
- ○ Other
- ○ Unknown

Field type: Radiobutton
Option group: Followup location
Variable name: LOF_FU_loc

If **16.3.2 Location for further follow-up** is equal to **Other** answer this question:

16.3.2.1 **Location follow-up***

Field type Multiline Textfield

Variable name LOF_FU_loc_other

Medical Events/history & Family history

17. Mortality

Mortality

17.1 **Last Updated***

DD MM YYYY

Error message shown if field's value is greater than **NOW**

No future dates can be entered

Field type: Date

Variable name: Mor_up_d

17.2 Date check

Calculated value

Error message shown if field's value is equal to **Error**

Date form last updated must be later than date first presentation at this hospital

Field type: Calculation

Variable name: Mor_up_d_check

17.3 **Died***

- Yes
- No

Field type: Radiobutton

Option group: Yes/No

Variable name: Mor_yn

If **17.3 Died** is equal to **Yes** answer this question:

17.3.1 **Date of death***

DD MM YYYY

Error message shown if field's value is greater than **NOW**

No future dates can be entered

Field type	Date	Variable name	Mor_d

If 17.3 Died is equal to **Yes** answer this question:

17.3.2 **Died during hospitalisation at our institution***

- Yes
- No

Field type	Radiobutton	Variable name	Mor_studysite_yn
Option group	Yes/No		

If 17.3.2 Died during hospitalisation at our institution is equal to **No** answer this question:

17.3.2.1 **GP contacted for cause of death?***

- No, GP still needs to be contacted
- No, GP will not be contacted (cause of death is clear)
- Yes, GP contacted
- Yes, GP contacted but no response after multiple attempts

Field type	Radiobutton	Variable name	Mor_GPcontact_yn
Option group	GP_contact_mor		

If 17.3 Died is equal to **Yes** answer this question:

17.3.3 **Sudden death***
Non-traumatic, unexpected fatal event occurring within 1 hour of the onset of symptoms (or within 24h if not witnessed).

- Yes
- No

Field type	Radiobutton	Variable name	Mor_sudden_yn
Option group	Yes/No		

If **17.3 Died** is equal to **Yes** answer this question:

17.3.4 **Cardiac death**•
• Autopsy has a cardiac or vascular anomaly as the probable cause of the event; OR • No obvious extra-cardiac causes have been by post-mortem examination and therefore an arrhythmic event is a likely cause of death.

- ◯ Yes
- ◯ No
- ◯ Unknown

Field type	Radiobutton	Variable name	Mor_y_car
Option group	Yes/No/Unknown		

If **17.3 Died** is equal to **Yes** answer this question:

17.3.5 **Reason death**•

Field type	Multiline Textfield	Variable name	Mor_oth

Medical Events/history & Family history

18. Family History

Family history

18.1 **Date medical information checked***
essential for future analyses, as it represents the most recent point up to which information is available

DD MM YYYY

Error message shown if field's value is greater than **NOW**

this cannot be a future date!

Field type Date

Variable name FH_upd_d

18.2 Date check

Calculated value

Error message shown if field's value is equal to **Error**

Date information checked must be later than date first presentation at this hospital

Field type Calculation

Variable name FH_upd_d_check

18.3 **Cardiomyopathy***

- Yes
- No
- Unknown

Field type Radiobutton
Option group Yes/No/Unknown

Variable name FH_CMP

If **18.3 Cardiomyopathy** is equal to **Yes** answer this question:

18.3.1 **Etiology of familial cardiomyopathy***

This checkbox field has too many options to list here. See Option Group - HF etiologie for its options.

Field type	Checkbox	Variable name	FH_HF_type
Option group	HF etiologie		

18.4 **Coronary interventions or myocardiac infarction (<65y)***

- Yes
- No
- Unknown

Field type	Radiobutton	Variable name	FH_Cor
Option group	Yes/No/Unknown		

18.5 Other familial diseases

Field type	Multiline Textfield	Variable name	FH_other

18.6 **Sudden cardiac death in famiy history***

- Yes
- No
- Unknown

Field type Radiobutton
Option group Yes/No/Unknown

Variable name FH_SCD

If **18.6 Sudden cardiac death in famiy history** is equal to **Yes**:

18.6.1 Family member with sudden cardiac death
(linked Repeating Data Form: "Familiy Member Sudden Cardiac Death")

Degree family member(s) with sudden cardiac death	Age of family member with sudden cardiac death	Cardiomyopathy Phenotype in the family member with SCD?	Family member description

Field type Repeated measures

Additional diagnostics

Additional diagnostics

19. Lab

19.1 **Lab analyses performed?***

- Yes
- No

Field type: Radiobutton
Option group: Yes/No
Variable name: lab_p

If 19.1 **Lab analyses performed?** is equal to **Yes** answer this question:

19.1.1 **Date last data update performed*** essential for future analyses, as it represents the most recent point up to which information is available

DD MM YYYY

Error message shown if field's value is greater than **NOW**

No future dates can be entered

Field type: Date
Variable name: lab_up_d

If 19.1 **Lab analyses performed?** is equal to **Yes**:

19.1.2 Laboratory analysis:
(linked Repeating Data Form: "Laboratory")

Created on	Submission method	Lab date	Lab time	Laboratory analysis location	serum hemoglobin	Serum hematocrit

Field type	Repeated measures	Variable name	lab_add

Additional diagnostics

20. ICD readout

20.1 **ICD readout performed?***

○ Yes

○ No

Field type: Radiobutton
Option group: Yes/No
Variable name: ICD_p

If **20.1 ICD readout performed?** is equal to **Yes** answer this question:

20.1.1 **Date last data update performed*** essential for future analyses, as it represents the most recent point up to which information is available

DD MM YYYY

Error message shown if field's value is greater than **NOW**

No future dates can be entered

Field type: Date
Variable name: ICD_up_d

If **20.1 ICD readout performed?** is equal to **Yes**:

20.1.2 ICD readouts
(linked Repeating Data Form: "ICD readout")

Created on	Date ICD readout	Date of first VT in current readout	VT count	Dominant VT cycle length	PVC count	ICD therapy given?

Field type: Repeated measures

Additional diagnostics

21. ECG

21.1 **ECG performed?***

- Yes
- No

Field type: Radiobutton
Option group: Yes/No
Variable name: ecg_p

If 21.1 **ECG performed?** is equal to **Yes** answer this question:

21.1.1 **Date last data update performed***
essential for future analyses, as it represents the most recent point up to which information is available

DD MM YYYY

Error message shown if field's value is greater than **NOW**

No future dates can be entered

Field type: Date
Variable name: ecg_up_d

If 21.1 **ECG performed?** is equal to **Yes**:

21.1.2 ECG:
(linked Repeating Data Form: "Electrocardiography")

Created on	Submission method	Date ECG	Rhythm	Rhythm other	Ventricular rate (average)	P axis

Field type: Repeated measures
Variable name: ecg_add

Additional diagnostics

22. Echocardiography

22.1 **Echocardiography performed?***

- Yes
- No

Field type: Radiobutton
Option group: Yes/No
Variable name: echo_p

If 22.1 **Echocardiography performed?** is equal to **Yes** answer this question:

22.1.1 **Date last data update performed***
essential for future analyses, as it represents the most recent point up to which information is available

DD MM YYYY

Error message shown if field's value is greater than **NOW**

No future dates can be entered

Field type: Date
Variable name: echo_up_d

If 22.1 **Echocardiography performed?** is equal to **Yes**:

22.1.2 Echocardiography overview:
(linked Repeating Data Form: "Echocardiography")

Created on	Information changed after Date data cleaned?	Variables changed? (report numbers of variables)	Submission method	Date echocardiography	Type of echocardiography	Location echocardiography performed

Field type: Repeated measures
Variable name: echo_add

Additional diagnostics

23. Holter

23.1 **Holter performed?***

○ Yes

○ No

Field type Radiobutton
Option group Yes/No

Variable name hol_p

If 23.1 **Holter performed?** is equal to **Yes** answer this question:

23.1.1 **Date last data update performed***

DD MM YYYY

Error message shown if field's value is greater than **NOW**

No future dates can be entered

Field type Date

Variable name hol_up_d

If 23.1 **Holter performed?** is equal to **Yes**:

23.1.2 Holter:
(linked Repeating Data Form: "Holter")

Created on	Submission method	Holter date performed (aankoppeldatum)	Duration holter registration	QRS complexes count total	Rhythm holter	Rhythm holter

Field type Repeated measures

Variable name hol_add

Additional diagnostics

24. CMR

24.1 **CMR performed?***

- ○ Yes
- ○ No

Field type: Radiobutton
Option group: Yes/No
Variable name: CMR_p

If 24.1 **CMR performed?** is equal to **Yes** answer this question:

24.1.1 **Date last data update performed***

DD | MM | YYYY

Error message shown if field's value is greater than **NOW**

No future dates can be entered

Field type: Date
Variable name: CMR_up_d

If 24.1 **CMR performed?** is equal to **Yes**:

24.1.2 CMR:
(linked Repeating Data Form: "Cardiac magnetic resonance (CMR)")

Created on	Submission method	Height	Weight	BSA	Date MRI	In which center is the CMR performed?

Field type: Repeated measures
Variable name: CMR_overview

Additional diagnostics

25. Genetics

25.1 **Date last data update performed***

DD MM YYYY

Error message shown if field's value is greater than **NOW**

No future dates can be entered

Field type: Date

Variable name: gen_up_d

25.2 **Received Genetic diagnostic testing***
Genetic diagnostic testing involves examining the patient's genome through blood analysis

- ○ Yes
- ○ No

Field type: Radiobutton
Option group: Yes/No

Variable name: gen_p

If **25.2 Received Genetic diagnostic testing** is equal to **Yes** answer this question:

25.2.1 **Genetic testing performed at which center***

- ☐ Amsterdam UMC
- ☐ Erasmus Universitair Medisch Centrum Rotterdam
- ☐ Leids Universitair Medisch Centrum
- ☐ Maastricht UMC+
- ☐ Radboud universitair medisch centrum
- ☐ Universitair Medisch Centrum Groningen
- ☐ Universitair Medisch Centrum Utrecht
- ☐ Other

Field type: Checkbox
Option group: Test_center

Variable name: genetic_testing_center

If 25.2 **Received Genetic diagnostic testing** is equal to **Yes** answer this question:

25.2.2 **Date of testing performed***

DD MM YYYY

Field type: Date
Variable name: date_of_testing_performed

If 25.2 **Received Genetic diagnostic testing** is equal to **Yes** answer this question:

25.2.3 **Individual gene tested***

- Yes
- No
- Unknown

Field type: Radiobutton
Option group: Yes/No/Unknown
Variable name: individual_gene_tested

If 25.2.3 **Individual gene tested** is equal to **Yes** answer this question:

25.2.3.1 **Which gene was tested?***

Field type: Textfield
Variable name: tested_gene_name

If 25.2 **Received Genetic diagnostic testing** is equal to **Yes** answer this question:

25.2.4 **Was Next-Generation Sequencing (NGS) performed?***

- Yes
- No

Field type: Radiobutton
Option group: Yes/No
Variable name: NGS_performed

If 25.2.4 **Was Next-Generation Sequencing (NGS) performed?** is equal to **Yes** answer this question:

25.2.4.1 **Which NGS was performed?***

- ☐ NGS cardiomyopathy
- ☐ NGS arrhythmia
- ☐ NGS other

Field type: Checkbox
Option group: NGS
Variable name: NGS_type

If 25.2 **Received Genetic diagnostic testing** is equal to **Yes** answer this question:

25.2.5 **Was Whole Exome Sequencing (WES) performed?***

- ○ Yes
- ○ No

Field type: Radiobutton
Option group: Yes/No
Variable name: WES_performed

If 25.2.5 **Was Whole Exome Sequencing (WES) performed?** is equal to **Yes** answer this question:

25.2.5.1 **Which WES was performed?***

- ☐ WES cardiomyopathy
- ☐ WES pediatric cardiomyopathy
- ☐ WES arrhythmia
- ☐ open exome

Field type: Checkbox
Option group: WES_type
Variable name: WES_type

If 25.2 **Received Genetic diagnostic testing** is equal to **Yes** answer this question:

25.2.6 **Was Whole Genome Sequencing (WGS) performed?***

- ○ Yes
- ○ No

Field type	Radiobutton	Variable name	WGS_performed
Option group	Yes/No		

If **25.2.6 Was Whole Genome Sequencing (WGS) performed?** is equal to **Yes** answer this question:

25.2.6.1 **Which WGS was performed?***

- ☐ WGS cardiomyopathy
- ☐ WGS pediatric cardiomyopathy
- ☐ WGS arrhythmia
- ☐ open genome

Field type	Checkbox	Option group	WGS

If **25.2 Received Genetic diagnostic testing** is equal to **Yes** answer this question:

25.2.7 **Was mitochondrial DNA (mtDNA) analysis performed?***

- ○ Yes
- ○ No

Field type	Radiobutton	Variable name	mtDNS_performed
Option group	Yes/No		

If **25.2 Received Genetic diagnostic testing** is equal to **Yes** answer this question:

25.2.8 **Was other genetic test performed?***

- ☐ Yes
- ☐ No

Field type	Checkbox	Variable name	Other_genetic_test_performed
Option group	Yes/No		

If **25.2.8 Was other genetic test performed?** is checked on **Yes** answer this question:

25.2.8.1 **Other_genetic_test_discription***

Field type	Textfield	Variable name	Other_genetic_test_discrip tion

25.3 **Family pseudonym***

Field type	Textfield	Variable name	gen_fam_pseudonym

25.4 **Pedigree present in PASS***

- Yes
- No

Field type<br>Option group	Radiobutton<br>Yes/No	Variable name	gen_pass

25.5 **Status***

- Index patient
- Family member

Field type<br>Option group	Radiobutton<br>Status of subject in genetic screening	Variable name	gen_status

If **25.5 Status** is equal to **Family member** answer this question:

25.5.1 **Degree of family member to index patient***

- First degree
- Second degree
- Third degree
- Unknown

Field type	Radiobutton	Variable name	gen_degree_fm
Option group	Degree of family member to index		

25.6 **Familial disease of cardiomyopathy***

- Yes
- No

Field type	Radiobutton	Variable name	gen_fam_cmp
Option group	Yes/No		

If 25.6 **Familial disease of cardiomyopathy** is equal to **Yes** answer this question:

25.6.1 **Degree family member***
A first-degree relative is a person's parent (father or mother), full sibling (brother or sister), or child. A second-degree relative is someone's uncles, aunts, nephews, nieces, grandparents, grandchildren, half-siblings. Third-degree relatives include first cousins, great grandparents, and great grandchildren

- ☐ First degree
- ☐ Second degree
- ☐ Third degree
- ☐ Unknown

Field type	Checkbox	Variable name	gen_fam_cmp_degree
Option group	Degree family member		

If 25.2 **Received Genetic diagnostic testing** is equal to **Yes**:

(likely) Pathogenic Mutations

If 25.2 **Received Genetic diagnostic testing** is equal to **Yes** answer this question:

25.2.10 **Class 4 or 5 mutation***

- Yes
- No

Field type	Radiobutton	Variable name	gen_lpp
Option group	Yes/No		

If 25.2.10 **Class 4 or 5 mutation** is equal to **Yes** answer this question:

25.2.10 **Gene name of 4/5 mutation***

This dropdown field has too many options to list here. See Option Group - GENE_LIST for its options.

Field type	Dropdown	Variable name	gen_lpp_gene
Option group	GENE_LIST		

If 25.2.10.1 **Gene name of 4/5 mutation** is equal to **Other** answer this question:

25.2.10.1.1 **gene LPP other***

Field type	Multiline Textfield	Variable name	gen_lpp_gene_other

If 25.2.10.1 **Gene name of 4/5 mutation** is equal to **TTN** answer this question:

25.2.10.1.2 **TTN variant position***

- A-band
- Z-disk
- I-band
- M-band

Field type	Dropdown	Variable name	gen_lpp_gene_TTN
Option group	TTN variant position		

If **25.2.10 Class 4 or 5 mutation** is equal to **Yes** answer this question:

25.2.10.2 **Nucleotide change of mutation***

Field type: Textfield
Variable name: gen_lpp_cpos

If **25.2.10 Class 4 or 5 mutation** is equal to **Yes** answer this question:

25.2.10.3 **Amino acid change of mutation***

Field type: Textfield
Variable name: gen_lpp_ppos

If **25.2.10 Class 4 or 5 mutation** is equal to **Yes** answer this question:

25.2.10.4 **Mutation zygosity***

- heterozygous
- homozygous
- hemizygous
- compound heterozygous

Field type: Radiobutton
Option group: zygosity
Variable name: gen_lpp_zygosity

If **25.2.10 Class 4 or 5 mutation** is equal to **Yes** answer this question:

25.2.10.5 **second Class 4 or 5 mutation***

- Yes
- No

Field type: Radiobutton
Option group: Yes/No
Variable name: gen_lpp2

If 25.2.10.5 second Class 4 or 5 mutation is equal to **Yes** answer this question:

25.2.10.5.1 **Gene name of 4/5 mutation***

This dropdown field has too many options to list here. See Option Group - GENE_LIST for its options.

Field type	Dropdown	Variable name	gen_lpp2_gene
Option group	GENE_LIST		

If 25.2.10.5.1 Gene name of 4/5 mutation is equal to **TTN** answer this question:

25.2.10.5.1.1 **TTN variant position***

- A-band
- Z-disk
- I-band
- M-band

Field type	Dropdown	Variable name	gen_lpp2_gene_TTN
Option group	TTN variant position LPP gene2		

If 25.2.10.5 second Class 4 or 5 mutation is equal to **Yes** answer this question:

25.2.10.5.2 **Nucleotide change of mutation***

Field type	Textfield	Variable name	gen_lpp2_cpos

If 25.2.10.5 second Class 4 or 5 mutation is equal to **Yes** answer this question:

25.2.10.5.3 **G.position***

Field type	Textfield	Variable name	G_position

If **25.2.10.5 second Class 4 or 5 mutation** is equal to **Yes** answer this question:

25.2.10.5.4 **Amino acid change of mutation***

Field type: Textfield
Variable name: gen_lpp2_ppos

If **25.2.10.5 second Class 4 or 5 mutation** is equal to **Yes** answer this question:

25.2.10.5.5 **Mutation zygosity***

- heterozygous
- homozygous
- hemizygous
- compound heterozygous

Field type: Radiobutton
Option group: zygosity
Variable name: gen_lpp2_zygosity

If **25.2 Received Genetic diagnostic testing** is equal to **Yes**:

Class 3 Mutations

If **25.2 Received Genetic diagnostic testing** is equal to **Yes** answer this question:

25.2.12 **Class 3 mutation***

- Yes
- No

Field type: Radiobutton
Option group: Yes/No
Variable name: gen_vus1

If **25.2 Received Genetic diagnostic testing** is equal to **Yes** answer this question:

25.2.13 **Cyp2c19 genotype***

- slow
- intermediate
- normal
- fast
- ultrafast

Field type	Radiobutton	Variable name	gen_vus1_Cyp2c19_genotype
Option group	Cyp2c19_genotype		

If 25.2.12 Class 3 mutation is equal to **Yes** answer this question:

25.2.12 **Gene name of class 3 mutation***

This dropdown field has too many options to list here. See Option Group - GENE_LIST for its options.

Field type	Dropdown	Variable name	gen_vus1_gene
Option group	GENE_LIST		

If 25.2.12.1 Gene name of class 3 mutation is equal to **TTN** answer this question:

25.2.12.1.1 **TTN variant position***

- A-band
- Z-disk
- I-band
- M-band

Field type	Dropdown	Variable name	gen_vus1_gene_TTN
Option group	TTN variant position vus1		

If 25.2.12 Class 3 mutation is equal to **Yes** answer this question:

25.2.12.2 **Nucleotide change of class 3 mutation***

Field type	Textfield	Variable name	gen_vus1_cpos

If 25.2.12 Class 3 mutation is equal to **Yes** answer this question:

25.2.12.3 **G.position***

Field type Textfield

Variable name G_position_1

If 25.2.12 **Class 3 mutation** is equal to **Yes** answer this question:

25.2.12.4 **Amino acid change of class 3 mutation***

Field type Textfield

Variable name gen_vus1_ppos

If 25.2.12 **Class 3 mutation** is equal to **Yes** answer this question:

25.2.12.5 **Mutation zygosity***

- heterozygous
- homozygous
- hemizygous
- compound heterozygous

Field type Radiobutton
Option group zygosity

Variable name gen_vus1_zygosity

If 25.2.12 **Class 3 mutation** is equal to **Yes** answer this question:

25.2.12.6 **Second Class 3 mutation***

- Yes
- No

Field type Radiobutton
Option group Yes/No

Variable name gen_vus2

If 25.2.12 **Class 3 mutation** is equal to **Yes** answer this question:

25.2.12.7 **Cyp2c19 genotype***

- slow
- intermediate
- normal
- fast
- ultrafast

Field type: Radiobutton
Option group: Cyp2c19_genotype
Variable name: gen_vus2_Cyp2c19_genotype

If 25.2.12.6 **Second Class 3 mutation** is equal to **Yes** answer this question:

25.2.12.6.1 **Gene name of class 3 mutation***

This dropdown field has too many options to list here. See Option Group - GENE_LIST for its options.

Field type: Dropdown
Option group: GENE_LIST
Variable name: gen_vus2_gene

If 25.2.12.6.1 **Gene name of class 3 mutation** is equal to **TTN** answer this question:

25.2.12.6.1.1 **TTN variant position***

- A-band
- Z-disk
- I-band
- M-band

Field type: Dropdown
Option group: TTN variant position vus2
Variable name: gen_vus2_gene_TTN

If 25.2.12.6 **Second Class 3 mutation** is equal to **Yes** answer this question:

25.2.12.6.2 **Nucleotide change of class 3 mutation***

Field type: Textfield
Variable name: gen_vus2_cpos

If 25.2.12.6 **Second Class 3 mutation** is equal to **Yes** answer this question:

25.2.12.6.3 **Amino acid change of class 3 mutation***

Field type: Textfield
Variable name: gen_vus2_ppos

If 25.2.12.6 **Second Class 3 mutation** is equal to **Yes** answer this question:

25.2.12.6.4 **Mutation zygosity***

- heterozygous
- homozygous
- hemizygous
- compound heterozygous

Field type: Radiobutton
Option group: zygosity
Variable name: gen_vus2_zygosity

If 25.2.12.6 **Second Class 3 mutation** is equal to **Yes** answer this question:

25.2.12.6.5 **Third Class 3 mutation***

- Yes
- No

Field type: Radiobutton
Option group: Yes/No
Variable name: gen_vus3

If 25.2.12.6 **Second Class 3 mutation** is equal to **Yes** answer this question:

25.2.12.6.6 **Cyp2c19 genotype***

- slow
- intermediate
- normal
- fast
- ultrafast

Field type Radiobutton
Option group Cyp2c19_genotype
Variable name gen_vus3_Cyp2c19_genotype

If **25.2.12.6.5 Third Class 3 mutation** is equal to **Yes** answer this question:

25.2.12.6.5.1 **Gene name of class 3 mutation***

This dropdown field has too many options to list here. See Option Group - GENE_LIST for its options.

Field type Dropdown
Option group GENE_LIST
Variable name gen_vus3_gene

If **25.2.12.6.5.1 Gene name of class 3 mutation** is equal to **TTN** answer this question:

25.2.12.6.5.1.1 **TTN variant position***

- A-band
- Z-disk
- I-band
- M-band

Field type Dropdown
Option group TTN variant position vus3
Variable name gen_vus3_gene_TTN

If **25.2.12.6.5 Third Class 3 mutation** is equal to **Yes** answer this question:

25.2.12.6.5.2 **Nucleotide change of class 3 mutation***

Field type Textfield
Variable name gen_vus3_cpos

If **25.2.12.6.5 Third Class 3 mutation** is equal to **Yes** answer this question:

25.2.12.6.5.3 **Amino acid change of class 3 mutation***

Field type: Textfield
Variable name: gen_vus3_ppos

If **25.2.12.6.5 Third Class 3 mutation** is equal to **Yes** answer this question:

25.2.12.6.5.4 **Mutation zygosity***

- heterozygous
- homozygous
- hemizygous
- compound heterozygous

Field type: Radiobutton
Option group: zygosity
Variable name: gen_vus3_zygosity

If **25.2.12.6.5 Third Class 3 mutation** is equal to **Yes** answer this question:

25.2.12.6.5.5 **Cyp-genotype***

- Yes
- No

Field type: Radiobutton
Option group: Yes/No
Variable name: cyp_genotype

If **25.2.12.6.5.5 Cyp-genotype** is equal to **Yes** answer this question:

25.2.12.6.5.5.1 **Which Cyp tested***

Field type: Textfield
Variable name: Cyp_test_type

Medical consultations at Follow-up

Medical consultations at Follow-up

26. Medical consultations

26.1 **Date last updated***
essential for future analyses, as it represents the most recent point up to which information is available

DD MM YYYY

Error message shown if field's value is greater than **NOW**

No future dates can be entered

Field type Date

Variable name OC_up_d

26.2 Date check

Calculated value

Error message shown if field's value is equal to **Error**

Date form last updated must be later than date first presentation at this hospital

Field type Calculation

Variable name OC_up_d_check

26.3 Add medical consultation
(linked Repeating Data Form: "Medical consultations")

Created on	Information changed after Date data cleaned?	Variables changed? (report numbers of variables)	Date of consultation	Type of consultation	Consult moment	NYHA classification

Field type	Repeated measures

Medical consultations at Follow-up

27. Medication

27.1 **Date last updated***

DD MM YYYY

Error message shown if field's value is greater than **NOW**

No future dates can be entered

Field type Date

Variable name med_up_d

27.2 Date check

Calculated value

Error message shown if field's value is equal to **Error**

Date form last updated must be later than date first presentation at this hospital

Field type Calculation

Variable name med_d_check

27.3 Medication
(linked Repeating Data Form: "Medication")

Created on	Date Medication	Medication moment	Beta-blocker

Field type Repeated measures

Biobanking

Biobanking

28. Biobanking

28.1 **Are there (tissue) samples available for CARDIO-NET?***

- ○ Yes
- ○ No
- ○ Unknown

Field type	Radiobutton	Variable name	biobank_available
Option group	Yes/No/Unknown		

If 28.1 **Are there (tissue) samples available for CARDIO...** is equal to **Yes** answer this question:

28.1.1 **Serum samples?***

- ○ Yes
- ○ No

Field type	Radiobutton	Variable name	biobank_serum
Option group	Yes/no		

If 28.1 **Are there (tissue) samples available for CARDIO...** is equal to **Yes** answer this question:

28.1.2 **Plasma samples?***

- ○ Yes
- ○ No

Field type	Radiobutton	Variable name	biobank_plasma
Option group	Yes/no		

If 28.1 **Are there (tissue) samples available for CARDIO...** is equal to **Yes** answer this question:

28.1.3 **Tissue samples?***

- ○ Yes
- ○ No

Field type Radiobutton
Option group Yes/no
Variable name biobank_tissue

If 28.1.3 **Tissue samples?** is equal to **Yes** answer this question:

28.1.3.1 **What tissue samples?***

- ☐ Endomyocardial biopsy
- ☐ LVAD sample
- ☐ HTx
- ☐ Other

Field type Checkbox
Option group Biobank_tissue_types
Variable name biobank_tissue_type

If 28.1.3.1 **What tissue samples?** is checked on **Other** answer this question:

28.1.3.1.1 **What other tissue types?***

Field type Multiline Textfield
Variable name biobank_tissue_type_other

Repeating Data

Cardiac magnetic resonance (CMR)

Cardiac magnetic resonance (CMR)

1. Cardiac magnetic resonance (CMR)

Report data

1.1 **Submission method***

- ○ manual
- ○ semi-automated

Field type	Radiobutton	Variable name	CMR_sub
Option group	Submission method		

1.2 **Height***

[cm]

Field type	Numeric field	Variable name	CMR_height
Limits	100 - 250		

1.3 **Weight***

[kg]

Field type	Numeric field	Variable name	CMR_weight
Limits	40 - 170		

1.4 BSA

Calculated value

Field type Calculation Variable name CMR_BSA

1.5 **Date MRI***

DD MM YYYY

Error message shown if field's value is greater than **NOW**

No future dates can be entered

Field type Date Variable name CMR_date

1.6 **In which center is the CMR performed?***

- this hospital
- Other

Field type Radiobutton Variable name CMR_location
Option group Study_site

If **1.6 In which center is the CMR performed?** is equal to **Other** answer this question:

1.6.1 **which center***

Field type Multiline Textfield Variable name CMR_location_other

1.7 **Type of MRI scanner***
GE (type) 1.5Tesla, etc

Field type Textfield Variable name CMR_scanner_type

1.8 **Amount of Tesla of MRI scanner***

- 1.5T
- 3T
- Other

Field type Radiobutton Variable name CMR_tesla
Option group CMR_tesla

If **1.8 Amount of Tesla of MRI scanner** is equal to **Other** answer this question:

1.8.1 **What other amount of Tesla?***

Field type Textfield Variable name CMR_tesla_other

LV volumina

1.9 **LVEDV***

mL

Field type Numeric field Variable name CMR_LV_EDV

1.10 LVEDV, indexed
man <35: 90 ± 11 (68-112) man >35: 75 ± 11 (53-97) vrouw <35: 80 ± 9 (62-98) vrouw >35: 73 ± 11 (51-95)

Calculated value

Field type Calculation Variable name CMR_LVEDVi

1.11 **LVESV***

mL

Field type Numeric field Variable name CMR_LV_ESV

1.12 LVESV, indexed
man <35: 30 ± 7 (16-44) man >35: 22 ± 6 (10-34) vrouw <35: 25 ± 6 (13-37) vrouw >35: 23 ± 6 (11-35)

Calculated value

Field type Calculation Variable name CMR_LVESVi

1.13 **LV mass***

gram

Field type Numeric field Variable name CMR_LVmass

1.14 LV mass, indexed
man <35: 67 ± 10 (47-87) man >35: 60 ± 9 (42-78) vrouw <35: 53 ± 9 (35-71) vrouw >35: 52 ± 9 (34-70) *Hudsmith et al. JCMR 2005* Man Vrouwnorm 50-95 35-75gering 96-130 76-115matig 131-190 116-150ernstig >190 >150*Salton et al. JACC 2002*

Calculated value

Field type Calculation Variable name CMR_LVmassi

1.15 LV stroke volume

Calculated value

Field type Calculation Variable name CMR_LVSV

1.16 LVEF

Calculated value

Field type Calculation Variable name CMR_LVEF

1.17 **LV cardiac output***

ml/min

Field type Numeric field Variable name CMR_LVCO

1.18 LV cardiac index

Calculated value

Field type Calculation Variable name CMR_LVCI

1.19 **Septal wall thickness***

mm

Field type Numeric field Variable name CMR_SWT

1.20 **Maximal wall thickness***

mm

Field type Numeric field Variable name CMR_MWT

RV volumina

1.21 **RVEDV***

ml

Field type Numeric field Variable name CMR_RVEDV

1.22 RVEDV, indexed
man <35: 104 ± 15 (74-134) man >35: 89 ± 11 (67-111)vrouw <35: 89 ± 11 (67-111) vrouw >35: 80 ± 19 (42-118)

Calculated value

Field type Calculation Variable name CMR_RVEDVi

1.23 **RVESV***

ml

Field type Numeric field Variable name CMR_RVESV

1.24 RVESV, indexed
man <35: 44 ± 9 (26-62) man >35: 34 ± 7 (20-48) vrouw <35: 35 ± 5 (25-45) vrouw >35: 30 ± 12 (6-54)

Calculated value

Field type Calculation
Variable name CMR_RVESVi

1.25 RV slagvolume

Calculated value

Field type Calculation
Variable name CMR_RVSV

1.26 RVEF

Calculated value

Field type Calculation
Variable name CMR_RVEF

1.27 **RV cardiac output***

ml/min

Field type Numeric field
Variable name CMR_RVCO

Other volumes

1.28 **LAVI***

Field type Numeric field Variable name CMR_LAVI

Valvulopathy

1.29 **Valvulopathy***

- ☐ aortic valve stenosis
- ☐ aortic valve insufficiency
- ☐ mitral valve stenosis
- ☐ mitral valve insufficiency
- ☐ tricuspid regurgitation
- ☐ other

Field type Checkbox Variable name CRM_valvulopathy
Option group CMR_valvulopathy

If **1.29 Valvulopathy** is checked on **other** answer this question:

1.29.1 **What other valvulopathy?***

Field type Textfield Variable name CMR_valvulopathy_other

Late gadolinium enhancement

1.30 **LGE present***

- ○ Yes
- ○ No

Field type Radiobutton Variable name CMR_LGE
Option group Yes/No

If **1.30 LGE present** is equal to **No** answer this question:

1.30.1 **Reason LGE missing***

- ○ Non-diagnostic
- ○ No LGE performed
- ○ Other

Field type	Radiobutton	Variable name	CMR_LGE_missing
Option group	LGE missing		

If **1.30 LGE present** is equal to **Yes** answer this question:

1.30.2 **Distribution of late gadolinium enhancement***

- ☐ Ischemic (subendocardial or transmural)
- ☐ Non-ischemic, epicardial
- ☐ Non-ischemic, mid-myocardial
- ☐ Patchy
- ☐ Non-ischemic, insertion RV
- ☐ Right ventricular enhancement
- ☐ Pericardial enhancement
- ☐ Other

Field type	Checkbox	Variable name	CMR_LGE_distrib
Option group	distributieLGE		

If **1.30.2 Distribution of late gadolinium enhancement** is checked on **Other** answer this question:

1.30.2.1 **What kind of other distribution of LGE?***

Field type	Textfield	Variable name	CMR_LGE_otherdistrib

If **1.30 LGE present** is equal to **Yes** answer this question:

1.30.3 **Location of late gadolinium enhancement***

- ☐ septal
- ☐ lateral
- ☐ inferoseptal
- ☐ inferolateral
- ☐ anteroseptal
- ☐ anterolateral
- ☐ inferior
- ☐ anterior

Field type: Checkbox
Option group: LGE location
Variable name: CMR_LGE_loc

If **1.30 LGE present** is equal to **Yes** answer this question:

1.30.4 **LGE quantification performed?***

- Yes
- No

Field type: Radiobutton
Option group: Yes/No
Variable name: CMR_LGE_quantified

If **1.30.4 LGE quantification performed?** is equal to **Yes** answer this question:

1.30.4.1 **LGE quantification performed with which software?***

- Qmedis
- CAAS

Field type: Radiobutton
Option group: LGE_quantification_software
Variable name: CMR_LGE_quantified_software

If **1.30.4 LGE quantification performed?** is equal to **Yes** answer this question:

1.30.4.2 **LGE amount (g)***

[g]

Field type: Numeric field
Variable name: CMR_LGE_amount

If **1.30.4 LGE quantification performed?** is equal to **Yes** answer this question:

1.30.4.3 LGE extent (%)

Calculated value

Field type Calculation
Variable name CMR_LGE_percentage

Tissue characterisation

1.31 **Tissue characterisation performed?***
T2-weighted images, T1 mapping, T2 mapping etc.

- ○ Yes
- ○ No

Field type Radiobutton
Option group Yes/No
Variable name CMR_mappingsequences

If 1.31 **Tissue characterisation performed?** is equal to **Yes** answer this question:

1.31.1 **T1 mapping performed?***

- ○ Yes
- ○ No

Field type Radiobutton
Option group Yes/No
Variable name CMR_T1mapperformed

If 1.31.1 **T1 mapping performed?** is equal to **Yes** answer this question:

1.31.1.1 **Native T1 myocard***

[ms]

Field type Numeric field
Variable name CMR_T1_native_myocard

If 1.31.1 **T1 mapping performed?** is equal to **Yes** answer this question:

1.31.1.2 **Post GD T1 mycoard***

[ms]

Field type Numeric field Variable name CMR_T1_postGD_myocard

If 1.31.1 T1 mapping performed? is equal to **Yes** answer this question:

1.31.1.3 **Native T1 blood***

ms

Field type Numeric field Variable name CMR_T1_native_blood

If 1.31.1 T1 mapping performed? is equal to **Yes** answer this question:

1.31.1.4 **Post GD T1 blood***

ms

Field type Numeric field Variable name CMR_T1_postGD_blood

If 1.31 Tissue characterisation performed? is equal to **Yes** answer this question:

1.31.2 **T2-weighted oedema images performed?***

- Yes
- No

Field type Radiobutton
Option group Yes/No
Variable name CMR_T2weighted

If 1.31.2 T2-weighted oedema images performed? is equal to **Yes** answer this question:

1.31.2.1 **T2 weighted images***

- Normal
- Abnormal (high)
- Non-diagnostic

Field type	Radiobutton	Variable name	CMR_T2weighted_conclusion
Option group	T2 weighted		

Myocardial Strain

1.32 **Strain analysis performed?***

- Yes
- No

Field type	Radiobutton	Variable name	CMR_strain_
Option group	Yes/No		

If **1.32 Strain analysis performed?** is equal to **Yes** answer this question:

1.32.1 **Which software was used for strain analysis?***

- Medis Qstrain
- Pie Medical

Field type	Radiobutton	Variable name	CMR_strain_software
Option group	CMR_strain_software		

If **1.32 Strain analysis performed?** is equal to **Yes** answer this question:

1.32.2 **Number of phases of cine images? (temporal resolution)***

Field type	Numeric field	Variable name	CMR_cines_TR

If **1.32 Strain analysis performed?** is equal to **Yes**:

Left Ventricular Strain

If **1.32 Strain analysis performed?** is equal to **Yes** answer this question:

1.32.4 **LV strain analysis performed?***

- ○ Yes
- ○ No

Field type: Radiobutton
Option group: Yes/No
Variable name: CMR_strain__LV

If **1.32.4 LV strain analysis performed?** is equal to **Yes**:

Left Ventricular Global Circumferential Strain

If **1.32.4 LV strain analysis performed?** is equal to **Yes** answer this question:

1.32.4.2 **LV GCS***

[ ] %

Field type: Numeric field
Variable name: CMR_LVGCS

If **1.32.4 LV strain analysis performed?** is equal to **Yes** answer this question:

1.32.4.3 **LV GCS TTP***

[ ] ms

Field type: Numeric field
Variable name: CMR_LVGCSTTP

If **1.32.4 LV strain analysis performed?** is equal to **Yes** answer this question:

1.32.4.4 **LV GCS ED fase***

[ ] ms

Field type: Numeric field
Variable name: CMR_LVGCS_ED

If **1.32.4 LV strain analysis performed?** is equal to **Yes** answer this question:

1.32.4.5 **Cardiac cycle duration mid-ventriculair shortaxis (TD***

ms

Field type Numeric field Variable name CMR_strainSAX_TD

If **1.32.4 LV strain analysis performed?** is equal to **Yes**:

Left Ventricular Global Radial Strain

If **1.32.4 LV strain analysis performed?** is equal to **Yes** answer this question:

1.32.4.7 **LV GRS***

%

Field type Numeric field Variable name CMR_LVGRS

If **1.32.4 LV strain analysis performed?** is equal to **Yes** answer this question:

1.32.4.8 **LV GRS TTP***

ms

Field type Numeric field Variable name CMR_LVGRSTTP

If **1.32.4 LV strain analysis performed?** is equal to **Yes**:

Left Ventricular Global Longitudinal Strain

If **1.32.4 LV strain analysis performed?** is equal to **Yes** answer this question:

1.32.4.10 **LV GLS***

%

Field type Numeric field Variable name CMR_LVGLS

If **1.32.4 LV strain analysis performed?** is equal to **Yes** answer this question:

1.32.4.11 **LV GLS TTP***

ms

Field type: Numeric field | Variable name: CMR_LVGLSTTP

If **1.32.4 LV strain analysis performed?** is equal to **Yes** answer this question:

1.32.4.12 **LV GLS ED fase***

ms

Field type: Numeric field | Variable name: CMR_LVGLS_ED

If **1.32.4 LV strain analysis performed?** is equal to **Yes** answer this question:

1.32.4.13 **Cardiac cycle duration longaxis (TD)***

ms

Field type: Numeric field | Variable name: CMR_strainLAX_TD

If **1.32.4 LV strain analysis performed?** is equal to **Yes** answer this question:

1.32.4.14 LV GLS 4-Chamber

%

Field type: Numeric field | Variable name: CMR_LVGLS_4Ch

If **1.32.4 LV strain analysis performed?** is equal to **Yes** answer this question:

1.32.4.15 LV GLS 4-Chamber TTP

ms

Field type Numeric field Variable name CMR_LVGLSTTP_4Ch

If 1.32.4 LV strain analysis performed? is equal to **Yes** answer this question:

1.32.4.16 LV GLS free wall

%

Field type Numeric field Variable name CMR_LVGLS_fw

If 1.32.4 LV strain analysis performed? is equal to **Yes** answer this question:

1.32.4.17 LV GLS free wall TTP

ms

Field type Numeric field Variable name CMR_LVGLSTTP_4Ch_fw

If 1.32.4 LV strain analysis performed? is equal to **Yes** answer this question:

1.32.4.18 LV GLS septal wall

%

Field type Numeric field Variable name CMR_LVGLS_sw

If 1.32.4 LV strain analysis performed? is equal to **Yes** answer this question:

1.32.4.19 LV GLS septal wall TTP

ms

Field type	Numeric field	Variable name	CMR_LVGLSTTP_4Ch_sw

If **1.32 Strain analysis performed?** is equal to **Yes**:

Right Ventricular Global Longitudinal Strain

If **1.32 Strain analysis performed?** is equal to **Yes** answer this question:

1.32.6 **RV strain analysis performed?***

- Yes
- No

Field type	Radiobutton	Variable name	CMR_strain__RV
Option group	Yes/No		

If **1.32.6 RV strain analysis performed?** is equal to **Yes** answer this question:

1.32.6.1 **RV GLS***

[ ] %

Field type	Numeric field	Variable name	CMR_RVGLS

If **1.32.6 RV strain analysis performed?** is equal to **Yes** answer this question:

1.32.6.2 **RV GLS TTP***

[ ] ms

Field type	Numeric field	Variable name	CMR_RVGLSTTP

If **1.32.6 RV strain analysis performed?** is equal to **Yes** answer this question:

1.32.6.3 **RV GLS ED fase***

[ ] ms

Field type Numeric field Variable name CMR_RVGLS_ED

If 1.32.6 **RV strain analysis performed?** is equal to **Yes** answer this question:

1.32.6.4 RV GLS free wall

%

Field type Numeric field Variable name CMR_RVGLS_fw

If 1.32.6 **RV strain analysis performed?** is equal to **Yes** answer this question:

1.32.6.5 RV GLS free wall TTP

ms

Field type Numeric field Variable name CMR_RVGLSTTP_fw

If 1.32.6 **RV strain analysis performed?** is equal to **Yes** answer this question:

1.32.6.6 RV GLS septal wall

%

Field type Numeric field Variable name CMR_RVGLS_sw

If 1.32.6 **RV strain analysis performed?** is equal to **Yes** answer this question:

1.32.6.7 RV GLS septal wall TTP

ms

Field type Numeric field Variable name CMR_RVGLSTTP_sw

If **1.32.6 RV strain analysis performed?** is equal to **Yes** answer this question:

1.32.6.8 **RV FAC***

Field type: Numeric field
Variable name: CMR_RVFAC

If **1.32 Strain analysis performed?** is equal to **Yes**:

Left Atrial Strain

If **1.32 Strain analysis performed?** is equal to **Yes** answer this question:

1.32.8 **LA strain analysis performed?***

- Yes
- No

Field type: Radiobutton
Option group: Yes/No
Variable name: CMR_strain_LA

If **1.32.8 LA strain analysis performed?** is equal to **Yes**:

Left Atrial Strain 2-Chamber

If **1.32.8 LA strain analysis performed?** is equal to **Yes** answer this question:

1.32.8.2 **LA reservoir (Es) 2-Ch*** %

Field type: Numeric field
Variable name: CMR_LA_reservoir_2Ch

If **1.32.8 LA strain analysis performed?** is equal to **Yes** answer this question:

1.32.8.3 **LA booster (Ea) 2-Ch*** %

Field type Numeric field Variable name CMR_LA_booster_2Ch

If 1.32.8 **LA strain analysis performed?** is equal to **Yes** answer this question:

1.32.8.4 LA conduit (Ea) 2-Ch

Calculated value

Field type Calculation Variable name CMR_LA_conduit_2Ch

If 1.32.8 **LA strain analysis performed?** is equal to **Yes** answer this question:

1.32.8.5 **LA EDV 2-Ch***

ml

Field type Numeric field Variable name CMR_LA_EDV_2Ch

If 1.32.8 **LA strain analysis performed?** is equal to **Yes** answer this question:

1.32.8.6 **LA ESV 2-Ch***

ml

Field type Numeric field Variable name CMR_LA_ESV_2Ch

If 1.32.8 **LA strain analysis performed?** is equal to **Yes** answer this question:

1.32.8.7 **LA EF 2-Ch***

%

Field type Numeric field Variable name CMR_LA_EF_2Ch

If **1.32.8 LA strain analysis performed?** is equal to **Yes**:

Left Atrial Strain 4-Chamber

If **1.32.8 LA strain analysis performed?** is equal to **Yes** answer this question:

1.32.8.9 **LA reservoir (Es) 4-Ch***

%

Field type: Numeric field
Variable name: CMR_LA_reservoir_4Ch

If **1.32.8 LA strain analysis performed?** is equal to **Yes** answer this question:

1.32.8.10 **LA booster (Ea) 4-Ch***

%

Field type: Numeric field
Variable name: CMR_LA_booster_4Ch

If **1.32.8 LA strain analysis performed?** is equal to **Yes** answer this question:

1.32.8.11 LA conduit (Ea) 4-Ch

Calculated value

Field type: Calculation
Variable name: CMR_LA_conduit_4Ch

If **1.32.8 LA strain analysis performed?** is equal to **Yes** answer this question:

1.32.8.12 **LA EDV 4-Ch***

ml

Field type: Numeric field
Variable name: CMR_LA_EDV_4Ch

If **1.32.8 LA strain analysis performed?** is equal to **Yes** answer this question:

1.32.8.13 **LA ESV 4-Ch**

ml

Field type: Numeric field
Variable name: CMR_LA_ESV_4Ch

If **1.32.8 LA strain analysis performed?** is equal to **Yes** answer this question:

1.32.8.14 **LA EF 4-Ch**

%

Field type: Numeric field
Variable name: CMR_LA_EF_4Ch

If **1.32.8 LA strain analysis performed?** is equal to **Yes**:

Left Atrial Strain Total

If **1.32.8 LA strain analysis performed?** is equal to **Yes** answer this question:

1.32.8.16 LA reservoir (Es) 4-Ch

Calculated value

Field type: Calculation
Variable name: CMR_LA_reservoir_total

If **1.32.8 LA strain analysis performed?** is equal to **Yes** answer this question:

1.32.8.17 LA booster strain (Ea) TOTAL

Calculated value

Field type: Calculation
Variable name: CMR_LA_booster_total

If **1.32.8 LA strain analysis performed?** is equal to **Yes** answer this question:

1.32.8.18 LA conduit strain (Ee) TOTAL

Calculated value

Field type: Calculation

Variable name: CMR_LA_conduit_total

If **1.32.8 LA strain analysis performed?** is equal to **Yes** answer this question:

1.32.8.19 LA EDV TOTAL

Calculated value

Field type: Calculation

Variable name: CMR_LA_EDV_total

If **1.32.8 LA strain analysis performed?** is equal to **Yes** answer this question:

1.32.8.20 LA ESV TOTAL

Calculated value

Field type: Calculation

Variable name: CMR_LA_ESV_total

If **1.32.8 LA strain analysis performed?** is equal to **Yes** answer this question:

1.32.8.21 LA EF TOTAL

Calculated value

Field type: Calculation

Variable name: CMR_LA_EF_total

Pericardial effusion

1.33 Pericardial effusion?

- Yes
- No

Field type: Radiobutton
Option group: Yes/No
Variable name: CMR_PE

If **1.33 Pericardial effusion?** is equal to **Yes** answer this question:

1.33.1 Pericardial effusion
Calculated as the maximal diameter in the ED phase

[ ] cm

Field type: Numeric field
Variable name: CMR_PE_cm

If **1.33 Pericardial effusion?** is equal to **Yes** answer this question:

1.33.2 Localisation pericardial effusion

- Global
- Focal
- Other

Field type: Radiobutton
Option group: PE lokalisatie
Variable name: CMR_PE_localisation

If **1.33.2 Localisation pericardial effusion** is equal to **Other** answer this question:

1.33.2.1 Specify 'other' localisation

Field type: Textfield
Variable name: CMR_PE_localisation_other

Pleural effusion

1.34 Pleural effusion

- ○ None
- ○ Left sided
- ○ Right sided
- ○ Bilateral

Field type	Radiobutton	Variable name	CMR_pleuraleffusion
Option group	pleural effusion		

Interpretation by radiologist

1.35 Does the radiologist suspect a specific disease?

- ☐ Cardiac amyloidosis
- ☐ Myocarditis
- ☐ Sarcoidosis
- ☐ Fabry
- ☐ Sarcomeric HCM

Field type	Checkbox	Variable name	CMR_interpret
Option group	CMR interpretation radiologist		

Echocardiography

Echocardiography

1. Echocardiography

Report data

1.1 **Information changed after Date data cleaned?***

- ○ Yes
- ○ No

Field type	Radiobutton	Variable name	echo_cleaned_rev
Option group	Yes/No		

If **1.1 Information changed after Date data cleaned?** is equal to **Yes** answer this question:

1.1.1 **Variables changed? (report numbers of variables)***

Field type	Textfield	Variable name	echo_cleaned_rev_info

1.2 **Submission method***
semi-automated is applicable when datalists are provided. If uncertain, choose manual.

- ○ manual
- ○ semi-automated

Field type	Radiobutton	Variable name	echo_sub
Option group	Submission method		

1.3 **Date echocardiography***
If date is not exactly known, use the median date as an estimation (15th of the month if exact day is unknown, or July 1st when only the year is known). If completely unknown, use 01-01-1800.

DD MM YYYY

Error message shown if field's value is greater than **NOW**

No future dates can be entered

Field type Date

Variable name echo_d

1.4 **Type of echocardiography***

- TTE
- TEE
- TTE & TEE
- Excercise echocardiography
- High-dose dobutamine test
- Low-dose dobutamine test
- Contrast echocardiography
- CMI echo
- Unknown type

Field type Dropdown
Option group echo_type

Variable name echo_type

1.5 **Location echocardiography performed***

- Heart function
- Operating room
- ICU
- ICU CTC
- ICCU
- First heart-aid
- Outpatient clinic
- General ward
- Other hospital

Field type Dropdown
Option group Location Echocardiography

Variable name echo_loc

1.6 **Cardiac rhythm during echocardiography***

This dropdown field has too many options to list here. See Option Group - Rhythm during echo for its options.

Field type	Dropdown	Variable name	echo_rhythm
Option group	Rhythm during echo		

1.7 **Heart frequency***

bpm

Warning message shown if field's value is greater or equal than **120**

Are you sure?

Warning message shown if field's value is smaller or equal than **40**

Are you sure?

Field type	Numeric field	Variable name	echo_HF
Limits	20 - 220		

1.8 **LVEF assessed by Teichholz method manually added***

%

Warning message shown if field's value is greater or equal than **70**

Are you sure?

Warning message shown if field's value is smaller or equal than **5**

Are you sure?

Field type	Numeric field	Variable name	echo_LVEF_teich_man

If a percentage range has been reported, provide the average of this range (eg 50-55% must be

supplied as 53%).

1.10 **LVEF assessed by biplane/2D***

%

ⓘ **Warning** message shown if field's value is greater or equal than **70**

Are you sure?

ⓘ **Warning** message shown if field's value is smaller or equal than **5**

Are you sure?

Field type Numeric field Variable name echo_LVEF_bip

1.11 **Left ventricular end-diastolic diameter***

mm

ⓘ **Warning** message shown if field's value is smaller or equal than **20**

Are you sure?

ⓘ **Warning** message shown if field's value is greater or equal than **150**

Are you sure?

Field type Numeric field Variable name echo_LVEDD

1.12 **Left ventricular end-systolic diameter***

mm

ⓘ **Warning** message shown if field's value is smaller or equal than **20**

Are you sure?

ⓘ **Warning** message shown if field's value is greater or equal than **120**

Are you sure?

Field type Numeric field Variable name echo_LVESD

1.13 Left ventricular end-diastolic volume

ml

ⓘ **Warning** message shown if field's value is greater or equal than **350**

Are you sure?

ⓘ **Warning** message shown if field's value is smaller or equal than **50**

Are you sure?

Field type Numeric field Variable name echo_EDV

1.14 Left ventricular end-systolic volume

ml

ⓘ **Warning** message shown if field's value is greater than **300**

Are you sure?

ⓘ **Warning** message shown if field's value is smaller or equal than **20**

Are you sure?

Field type	Numeric field	Variable name	echo_ESV
Limits	10 - 999		

1.15 **Global logitudinal Strain (GLS)***

%

Field type	Numeric field	Variable name	echo_GLS

1.16 **GLS pattern***

- ○ normal
- ○ apical sparing
- ○ diffuse
- ○ regional

Field type	Radiobutton	Variable name	echo_GLS_pattern
Option group	GLS pattern		

1.17 **gradient LVOT (rest)***

Field type	Numeric field	Variable name	echo_lvot_rest

1.18 **gradient LVOT (Valsalva)***

Field type	Numeric field	Variable name	echo_lvot_valsalva

1.19 **gradient LVOT (exercise, e.g. 20 squats)***

Field type	Numeric field	Variable name	echo_lvot_exercise

1.20 **gradient mid-cavitair***

- ○ Yes
- ○ No
- ○ Unknown

Field type<br>Option group	Radiobutton<br>Yes/No/Unknown	Variable name	echo_mid_cavitair

If **1.20 gradient mid-cavitair** is equal to **Yes** answer this question:

1.20.1 **gradient mid-cavitair (value)***

Field type	Numeric field	Variable name	echo_mid_cavitair_value

1.21 **RV function***
Eyeballing. An empty value cannot be interpreted as normal RV function but should be checked again (evaluation often present in free text form).

- ○ Good
- ○ Moderate
- ○ Bad
- ○ Unknown

Field type Dropdown
Option group RV function

Variable name echo_RVFunc

1.22 **Basal right ventricle diameter***

mm

Warning message shown if field's value is greater or equal than **75**

Are you sure?

Warning message shown if field's value is smaller or equal than **15**

Are you sure?

Field type Numeric field

Variable name echo_RVD_basal

1.23 **Intraventricular septal thickness***

mm

Warning message shown if field's value is greater or equal than **15**

Are you sure?

Warning message shown if field's value is smaller or equal than **5**

Are you sure?

Field type Numeric field

Variable name echo_IVS

1.24 **Left ventricular posterior wall thickness***

mm

ⓘ **Warning** message shown if field's value is greater or equal than **15**

Are you sure?

ⓘ **Warning** message shown if field's value is smaller or equal than **5**

Are you sure?

Field type	Numeric field	Variable name	echo_LVPW
Limits	2 - 70		

1.25 **Left ventricular mass***

g

ⓘ **Warning** message shown if field's value is greater or equal than **500**

Are you sure?

ⓘ **Warning** message shown if field's value is smaller or equal than **45**

Are you sure?

Field type	Numeric field	Variable name	echo_LVmass
Limits	1 - 1500		

1.26 **Left ventricular mass index***

g/m2

ⓘ **Warning** message shown if field's value is smaller or equal than **25**

Are you sure?

ⓘ **Warning** message shown if field's value is greater or equal than **150**

Are you sure?

Field type Numeric field

Variable name echo_LVMI

1.27 **Left atrial diameter***

mm

ⓘ **Warning** message shown if field's value is greater or equal than **100**

Are you sure?

ⓘ **Warning** message shown if field's value is smaller or equal than **20**

Are you sure?

Field type Numeric field

Limits 10 - 999

Variable name echo_LA_d

1.28 **Left atrial volume***

ml

ⓘ **Warning** message shown if field's value is greater or equal than **200**

Are you sure?

ⓘ **Warning** message shown if field's value is smaller or equal than **20**

Are you sure?

Field type Numeric field
Limits 10 - 999
Variable name echo_LA_vol

1.29 **Left atrial volume index***

ml/m^2

ⓘ **Warning** message shown if field's value is smaller or equal than **5**

Are you sure?

ⓘ **Warning** message shown if field's value is greater or equal than **75**

Are you sure?

Field type Numeric field
Limits 10 - 999
Variable name echo_LAVI

1.30 **Right atrial volume***

ml

ⓘ **Warning** message shown if field's value is smaller or equal than **15**

Are you sure?

ⓘ **Warning** message shown if field's value is greater or equal than **150**

Are you sure?

Field type	Numeric field	Variable name	echo_RA_vol
Limits	5 - 999		

1.31 **Vena cava inferior diameter during expiration***

mm

ⓘ **Warning** message shown if field's value is greater or equal than **70**

Are you sure?

Field type	Numeric field	Variable name	echo_VCI_Exp

1.32 **Vena cava collapse***

%

Field type	Numeric field	Variable name	echo_VCIcol

1.33 **Estimated right ventricular pressure***

mmHg

ⓘ **Warning** message shown if field's value is smaller or equal than **10**

Are you sure?

ⓘ **Warning** message shown if field's value is greater or equal than **75**

Are you sure?

Field type Numeric field Variable name echo_EstRVP

1.34 **TR velocity***

m/sec

ⓘ **Warning** message shown if field's value is smaller or equal than **10**

Are you sure?

ⓘ **Warning** message shown if field's value is greater or equal than **75**

Are you sure?

Field type Numeric field Variable name echo_TR_velocity

1.35 **Mitral early filling (E-top)***

cm/s

ⓘ **Warning** message shown if field's value is greater or equal than **250**

Are you sure?

ⓘ **Warning** message shown if field's value is smaller or equal than **10**

Are you sure?

Field type	Numeric field	Variable name	echo_Etop
Limits	5 - 999		

1.36 **Mitral active filling (A-top)***

cm/s

ⓘ **Warning** message shown if field's value is smaller or equal than **5**

Are you sure?

ⓘ **Warning** message shown if field's value is greater or equal than **200**

Are you sure?

Field type	Numeric field	Variable name	echo_Atop
Limits	3 - 999		

1.37 **Aortic outflowtract narrowing***

- ☐ valvular AS (> 2 m/s)
- ☐ LVOT stenosis (dynamic end-systolic gradient)
- ☐ intraventricular gradient (dynamic end-systolic gradient)
- ☐ subvalvular AS
- ☐ supravalvular AS
- ☐ unknown

Field type Checkbox
Option group Aortic outflowtract narrowing

Variable name echo_aov_outflow

1.38 **Mitral E/A ratio**

Field type Numeric field

Variable name echo_EA

1.39 Type diastolic dysfunction based on E/A ratio

- normal
- type 1 (abnormal)
- type 2 (pseudonormal)
- type 3 (restrictive)
- unknown

Field type Radiobutton
Option group E/A diastolic dysfunction

Variable name echo_ea_t

1.40 **Mitral early deceleration time**

ms

Warning message shown if field's value is smaller or equal than **50**

Are you sure?

Warning message shown if field's value is greater or equal than **500**

Are you sure?

Field type Numeric field
Limits 10 - 999

Variable name echo_DT

1.41 **Tricuspidal regurgitation***

m/s

ⓘ **Warning** message shown if field's value is greater or equal than **10**

Are you sure?

Field type Numeric field

Variable name echo_Ti

1.42 **Peak e' speed IVS***

cm/s

ⓘ **Warning** message shown if field's value is smaller or equal than **3**

Are you sure?

ⓘ **Warning** message shown if field's value is greater or equal than **15**

Are you sure?

Field type Numeric field

Variable name echo_eprim_IVS

1.43 **E/e' IVS***

Field type Numeric field

Variable name echo_Eeprim_IVS

1.44 **Peak e' speed LV lateral***

cm/s

ⓘ **Warning** message shown if field's value is smaller or equal than **3**

Are you sure?

ⓘ **Warning** message shown if field's value is greater or equal than **15**

Are you sure?

Field type Numeric field Variable name echo_eprim_lvlat

1.45 **E/e' LV lateral***

ⓘ **Warning** message shown if field's value is greater or equal than **30**

Are you sure?

ⓘ **Warning** message shown if field's value is smaller or equal than **3**

Are you sure?

Field type Numeric field Variable name echo_eeprim_lvlat

1.46 **E/e' average ivs and LV lat***

Field type Numeric field Variable name echo_eeprim_ave

1.47 **TAPSE***
Tricuspid annular plane systolic excursion

mm

ⓘ **Warning** message shown if field's value is smaller or equal than **5**

Are you sure?

ⓘ **Warning** message shown if field's value is greater or equal than **50**

Are you sure?

Field type Numeric field Variable name echo_TAPSE

1.48 S' right ventricule

cm/s

Field type Numeric field Variable name echo_srv

1.49 S2-top of pulmonary vein

cm/s

Field type Numeric field Variable name echo_pv_s2

1.50 D-top of pulmonary vein

ms

Field type Numeric field Variable name echo_pv_d

1.51 Deceleration time D-top of pulmonary vein

ms

Field type	Numeric field	Variable name	echo_pv_d_decel
Limits	10 - 999		

1.52 PV S/D ratio

Field type	Numeric field	Variable name	echo_pv_sd

1.53 PV ar flow reversal

cm/s

Field type	Numeric field	Variable name	echo_pv_ar

1.54 Regional wall abnormalities

- Yes
- No
- Unknown

Field type	Radiobutton	Variable name	echo_wall_abn
Option group	Yes/No/Unknown		

Cardiac valve disease

1.55 **Maximum gradient aortic valve***

mmHg

Field type	Numeric field	Variable name	echo_aov_maxgrad

1.56 **Mean gradient aortic valve***

mmHg

Field type: Numeric field
Variable name: echo_aov_meangrad

1.57 **Aortic valve area (AVA)***

cm^2

Field type: Numeric field
Variable name: echo_aov_ava

1.58 **Aortic Effective orifice area (EOA)***

cm^2

Field type: Numeric field
Variable name: echo_aov_eoa

1.59 **Aortic valve insufficiency (based on ERO)***
If ERO was measured, please also record this

- Trace of AI
- Moderate AI (ERO <0,1 cm2)
- Considerable AI (ERO 0,1-0,2)
- Fairly Severe AI (ERO0,2-0,3cm2)
- Severe AI (ERO>0,3cm2)
- No AI
- Unknown

Field type: Dropdown
Option group: AI severity
Variable name: echo_aov_insuf

1.60 **Aortic valve insufficiency (AOI classification)***

- No AI
- Trace AI
- Mild AI
- Moderate AI
- Severe AI
- Unknown

Field type	Dropdown	Variable name	eho_aov_insuf_AOI
Option group	echo_aov_insuf_AOI		

1.61 **Mitral valve insufficiency (based on ERO)***
if ERO was measured, please also record this

- ○ Trace of MI
- ○ Slightly (ERO<0,2cm2)
- ○ Moderate MI (ERO 0,2-0,3cm2)
- ○ Fairly severe MI (ERO 0,3-0,4)
- ○ Severe MI (ERO>0,4cm2)
- ○ No
- ○ Unknown

Field type	Dropdown	Variable name	echo_miv_insuf
Option group	MI seriousness		

1.62 **Mitral valve insufficiency (AOI classification)***

- ○ No AI
- ○ Trace AI
- ○ Mild AI
- ○ Moderate AI
- ○ Severe AI
- ○ Unknown

Field type	Dropdown	Variable name	echo_miv_insuf_AOI
Option group	echo_aov_insuf_AOI		

If **1.61 Mitral valve insufficiency (based on ERO)** is not equal to **Trace of MI** answer this question:

1.61.1 **Mechanism of mitral valve insufficiency***

- ○ Systolic anterior motion (SAM)
- ○ Leaflet perforation
- ○ Annular dilatation (Type 1)
- ○ Leaflet prolaps (Type 2)
- ○ Leaflet restriction (Type 3)

Field type	Radiobutton	Variable name	echo_miv_insuf_mechanism
Option group	miv_insuf_mechanism		

If **1.61 Mitral valve insufficiency (based on ERO)** is not equal to **Trace of MI** answer this question:

1.61.2 **Type of leaflet restriction***

- ○ Inflammatory (type 3a)
- ○ Ischaemic (type 3b)

Field type	Radiobutton	Variable name	echo_miv_insuf_mechanism_leaf_res
Option group	miv_insuf_leaf_res_type		

1.63 **Mitral annulus calcification***
When the mitral annulus calcification is graded as "slight-moderate", please choose "moderate", and when it is graded as "moderate-severe", please choose "severe".

- ○ None
- ○ Slight
- ○ Moderate
- ○ Severe
- ○ Unknown

Field type	Radiobutton	Variable name	echo_miv_calc
Option group	MS calcification		

1.64 **Mitral valve gradient***

- ☐ Rheumatic MS (Carpentier IIIa)
- ☐ Mitral ostium reduction
- ☐ Mitral valve reconstruction
- ☐ Unknown

Field type	Checkbox	Variable name	echo_miv_grad
Option group	MV gradient		

1.65 **Maximum gradient mitral valve***

[mmHg]

Field type	Numeric field	Variable name	echo_miv_maxgrad

1.66 **Mean gradient mitral valve***

[mmHg]

Field type	Numeric field	Variable name	echo_miv_meangrad

1.67 **Mitral valve area**•

[cm^2]

Field type	Numeric field	Variable name	echo_miv_area

1.68 **Pulmonary valve stenosis**•

- ☐ Subvalvular
- ☐ Valvular
- ☐ Supravalvular
- ☐ No
- ☐ Unknown

Field type	Checkbox	Variable name	echo_puv_sten
Option group	PIV stenosis		

1.69 **Maximum gradient pulmonic valve**•

[mmHg]

Field type	Numeric field	Variable name	echo_puv_maxgrad

1.70 **Mean gradient pulmonic valve**•

[mmHg]

Field type	Numeric field	Variable name	echo_puv_meangrad

1.71 **Seriousness of pulmonic valve insufficiency***

- ○ Slightly
- ○ Moderate
- ○ Severe
- ○ None
- ○ Unknown

Field type: Dropdown
Option group: PI severity
Variable name: echo_puv_insuf

1.72 **Tricuspid valve stenosis***

- ☐ Rheumatic TS
- ☐ Tricuspid valve reconstruction
- ☐ Tricuspid valve prosthesis
- ☐ None
- ☐ Unknown

Field type: Checkbox
Option group: TRS severity
Variable name: echo_trv_sten

1.73 **Maximum gradient tricuspid valve***

[mmHg]

Field type: Numeric field
Variable name: echo_trv_maxgrad

1.74 **Mean gradient tricuspid valve***

[mmHg]

Field type: Numeric field
Variable name: echo_trv_meangrad

1.75 **Seriousness of tricuspid valve insufficiency***

- ○ Slightly
- ○ Moderate
- ○ Severe
- ○ None
- ○ Unknown

Field type	Dropdown	Variable name	echo_trv_insuf
Option group	PI severity		

ICD readout

ICD readout

1. ICD readout

1.1 **Date ICD readout***

DD MM YYYY

Error message shown if field's value is greater than **NOW**

No future dates can be entered

Field type	Date	Variable name	ICD_read_d

1.2 **Date of first VT in current read-out***

DD MM YYYY

Error message shown if field's value is greater than **NOW**

No future dates can be entered

Field type	Date	Variable name	ICD_read_first_VT

1.3 **VT count***

Field type	Numeric field	Variable name	ICD_read_VT_count

If **1.3 VT count** is greater than **0** answer this question:

1.3.1 **Dominant VT cycle length***

ms

Field type	Numeric field	Variable name	ICD_read_VT_CL

1.4 PVC count

Field type	Numeric field	Variable name	ICD_read_PVC_count

1.5 **ICD therapy given?***

- ○ Yes
- ○ No

Field type<br>Option group	Radiobutton<br>Yes/No	Variable name	ICD_read_therapy_yn

If **1.5 ICD therapy given?** is equal to **Yes** answer this question:

1.5.1 **Date of first ICD therapy***

DD MM YYYY

Error message shown if field's value is greater than **NOW**

No future dates can be entered

Field type	Date	Variable name	ICD_read_therapy_d

If **1.5 ICD therapy given?** is equal to **Yes** answer this question:

1.5.2 **ATP(s)?***

- ○ Yes
- ○ No

Field type Radiobutton
Option group Yes/no
Variable name ICD_read_ATP_yn

If **1.5.2 ATP(s)?** is equal to **Yes** answer this question:

1.5.2.1 **How many ATP(s)?***

Field type Numeric field
Variable name ICD_read_ATP_nr

If **1.5.2 ATP(s)?** is equal to **Yes** answer this question:

1.5.2.2 **Were the ATP(s) appropriate?***

- Only appropriate
- Only inappropriate
- Both appropriate and inappropriate

Field type Radiobutton
Option group ICD shocks appr/inappr
Variable name ICD_read_ATP_appr

If **1.5.2.2 Were the ATP(s) appropriate?** is equal to **Both appropriate and inappropriate** answer this question:

1.5.2.2.1 **Number of inappropriate ATP(s)?***

Field type Numeric field
Variable name ICD_read_ATP_inappr_nr

If **1.5.2.2 Were the ATP(s) appropriate?** is equal to **Both appropriate and inappropriate** answer this question:

1.5.2.2.2 Check ATP numbers

Calculated value

ⓘ **Warning** message shown if field's value is equal to **Error**

Number of inappropriate shocks is greater than the total number of shocks

Field type Calculation
Variable name check_ATP_numbers

If **1.5 ICD therapy given?** is equal to **Yes** answer this question:

1.5.3 **ICD shock(s)?***

- Yes
- No

Field type Radiobutton
Option group Yes/no
Variable name ICD_read_shock_yn

If **1.5.3 ICD shock(s)?** is equal to **Yes** answer this question:

1.5.3.1 **How many shock(s)?***

Field type Numeric field
Variable name ICD_read_shock_nr

If **1.5.3 ICD shock(s)?** is equal to **Yes** answer this question:

1.5.3.2 **Were the shock(s) appropriate?***

- Only appropriate
- Only inappropriate
- Both appropriate and inappropriate

Field type Radiobutton
Option group ICD shocks appr/inappr
Variable name ICD_read_shock_appr

If **1.5.3.2 Were the shock(s) appropriate?** is equal to **Both appropriate and inappropriate** answer this question:

1.5.3.2.1 **Number of inappropriate shock(s)?***

Field type	Numeric field	Variable name	ICD_read_shock_inappr_nr

If **1.5.3.2 Were the shock(s) appropriate?** is equal to **Both appropriate and inappropriate** answer this question:

1.5.3.2.2 Check shock numbers

Calculated value

Warning message shown if field's value is equal to **Error**

Number of inappropriate shocks is greater than the total number of shocks

Field type	Calculation	Variable name	check_shock_numbers

Medication

Medication

1. Medication

Report data

1.1 **Date Medication***

DD MM YYYY

Error message shown if field's value is greater than **NOW**

No future dates can be entered

Field type Date

Variable name Med_d

1.2 **Medication moment***

- Before outpatient visit
- After outpatient visit
- Hospital admission
- Hospital discharge
- day of EMB (both elective and non-elective)
- day of OMT

ⓘ **Warning** message shown if field's value is equal to **1**

Fill in complete medication list before outpatient visit

ⓘ **Warning** message shown if field's value is equal to **3**

Fill in complete medication list at hospital admission

ⓘ **Warning** message shown if field's value is equal to **2**

If complete list is already present BEFORE outpatient visit, only submit changes during outpatient visit

ⓘ **Warning** message shown if field's value is equal to **4**

If complete list is already present at admission, only submit changes compared to admission

Field type	Dropdown	Variable name	Med_moment
Option group	Clinical context		

If **1.3 Medication list calculated** is equal to **check** answer this question:

1.3.1 **Full medication list or only changes?***

- ◯ FULL list
- ◯ ONLY changes
- ◯ NO changes made

ⓘ **Warning** message shown if field's value is equal to **2**

Submit medication changes: stopped medication (>no), started medication (>yes, with dose), increased/decreased dose (>yes, new dose)

ⓘ **Warning** message shown if field's value is equal to **3**

Please be sure that a FULL list of medication prior this outpatient contact has been submitted to Castor. Otherwise no data regarding medication is stored. If a full list has been submitted, you can lock and close this report.

Field type	Radiobutton	Variable name	Med_list
Option group	Medication list		

If **1.3.1 Full medication list or only changes?** is not equal to **FULL list** answer this question:

1.3.1.1 **Date of FULL medication list. Changes are noted compared to this full list.***
Note down the date of the last FULL medication list prior this list of ONLY changes. This allows a check to make sure ONLY changes are connected to the correct FULL lists, to avoid any miss of medication changes. If the date of the FULL list is not representative, create a new FULL list.

DD MM YYYY

ⓘ **Warning** message shown if field's value is greater than **1800-01-01**

Make sure to check if a full list of medication is available in Castor on the date you just selected. If it does not exist, create a new full list for that date after this report submission.

Field type	Date	Variable name	Med_change_comp_d

1.4 **Cardiac myosin inhibitor***

- ○ Yes
- ○ No

Field type	Radiobutton	Variable name	med_CarMyosinInhib
Option group	Yes/No		

If **1.4 Cardiac myosin inhibitor** is equal to **Yes** answer this question:

1.4.1 **Cardiac myosin inhibitor type***

- ○ Mavacamten
- ○ Aficamten

Field type	Dropdown	Variable name	med_CarMyosinInhib_type
Option group	Cardiac Myosin Inhibitor Type		

If **1.4 Cardiac myosin inhibitor** is equal to **Yes** answer this question:

1.4.2 **Cardiac myosin inhibitor daily dose***

[mg]

Field type	Numeric field	Variable name	med_CarMyosinInhib_dose

1.5 **Beta-blocker***

- ○ Yes
- ○ No

Field type	Radiobutton	Variable name	med_bb
Option group	Yes/No		

If **1.5 Beta-blocker** is equal to **No** answer this question:

1.5.1 **Reason no beta-blocker***

- No, due to side-effect
- No, due to allergy
- No, due to contra-indication
- No, still to be started during uptitration
- No, due to no indication
- No, reason unknown

Field type: Dropdown
Option group: Medication no reason
Variable name: med_bb_no_r

If **1.5 Beta-blocker** is equal to **Yes** answer this question:

1.5.2 **Beta-blocker type***

This dropdown field has too many options to list here. See Option Group - Beta Blocker Types for its options.

Field type: Dropdown
Option group: Beta Blocker Types
Variable name: med_bb_type

If **1.5 Beta-blocker** is equal to **Yes** answer this question:

1.5.3 **Beta-blocker daily dose***
Target dose (mg) Bisoprolol 10 o.d. Carvedilol 25 b.i.d. Metoprolol succinate 200 o.d. Nebivolol 10 o.d.

[ ] mg

Field type: Numeric field
Variable name: med_bb_dose

If **1.5 Beta-blocker** is equal to **Yes** answer this question:

1.5.4 Beta-blocker OMT%

Calculated value

Field type	Calculation	Variable name	med_bb_OMT

If **1.5.4 Beta-blocker OMT%** is smaller than **100** answer this question:

1.5.4.1 Reason beta-blocker not OMT

- ◯ Due to contra-indication
- ◯ Due to uptitration phase ongoing
- ◯ Due to side-effects
- ◯ Due to no indication for heart failure OMT
- ◯ Due to maximum tolerable dose achieved
- ◯ Unknown

Field type	Dropdown	Variable name	med_bb_OMT_r
Option group	Reason no OMT		

1.6 **ACE-inhibitor***

- ◯ Yes
- ◯ No

Field type	Radiobutton	Variable name	med_acei
Option group	Yes/No		

If **1.6 ACE-inhibitor** is equal to **No** answer this question:

1.6.1 **Reason no ACE-inhibitor***

- ◯ No, due to side-effects
- ◯ No, due to allergy
- ◯ No, due to contra-indication
- ◯ No, due to ARNI use
- ◯ No, due to ARB use
- ◯ No, due to no indication
- ◯ No, reason unknown
- ◯ No, still to be started during uptitration

Field type	Dropdown	Variable name	med_acei_no_r
Option group	ACE no reason		

If **1.6 ACE-inhibitor** is equal to **Yes** answer this question:

1.6.2 **ACE-inhibitor type***

- Captopril
- Enalapril
- Fosinopril
- Lisinopril
- Perindopril
- Quinapril
- Ramipril
- Zofenopril
- Other

Field type: Dropdown
Option group: ACEi
Variable name: med_acei_type

Perindopril 4 o.d. (8 for post Myocardial infarction)

If **1.6 ACE-inhibitor** is equal to **Yes** answer this question:

1.6.4 ACE-inhibitor OMT%

Calculated value

Field type: Calculation
Variable name: med_acei_OMT

If **1.6.4 ACE-inhibitor OMT%** is smaller than **100** answer this question:

1.6.4.1 Reason ACE-inhibitor not OMT

- Due to contra-indication
- Due to uptitration phase ongoing
- Due to side-effects
- Due to no indication for heart failure OMT
- Due to maximum tolerable dose achieved
- Unknown

Field type: Dropdown
Option group: Reason no OMT
Variable name: med_acei_OMT_r

1.7 **Angiotensin receptor antagonist***

- Yes
- No

Field type Radiobutton
Option group Yes/No

Variable name med_arb

If **1.7 Angiotensin receptor antagonist** is equal to **No** answer this question:

1.7.1 **Reason no ARB***

- No, due to side-effects
- No, due to allergy
- No, due to contra-indication
- No, due to ARNI use
- No, due to ACE use
- No, due to no indication
- No, reason unknown
- No, still to be started during uptitration

Field type Dropdown
Option group ARB no reason

Variable name med_arb_no_r

If **1.7 Angiotensin receptor antagonist** is equal to **Yes** answer this question:

1.7.2 **Angiotensin receptor antagonist type***

- Candesartan
- Eprosartan
- Irbesartan
- Losartan
- Olmesartan
- Telmisartan
- Valsartan
- Other

Field type Dropdown
Option group ARB

Variable name med_arb_type

If **1.7 Angiotensin receptor antagonist** is equal to **Yes** answer this question:

1.7.3 **Angiotensin receptor antagonist daily dosage***
Target dose (mg) Candesartan 32 o.d.
Valsartan 160 b.i.d.
Losartan 150 o.d.

[ ] mg

Field type	Numeric field	Variable name	med_arb_dose

If **1.7 Angiotensin receptor antagonist** is equal to **Yes** answer this question:

1.7.4 Angiotensin receptor antagonist OMT%

Calculated value

Field type	Calculation	Variable name	med_arb_OMT

If **1.7.4 Angiotensin receptor antagonist OMT%** is smaller than **100** answer this question:

1.7.4.1 Reason ARB not OMT

- Due to contra-indication
- Due to uptitration phase ongoing
- Due to side-effects
- Due to no indication for heart failure OMT
- Due to maximum tolerable dose achieved
- Unknown

Field type	Dropdown	Variable name	med_arb_OMT_r
Option group	Reason no OMT		

1.8 **Mineralocorticoid receptor antagonist (kaliumsparend diureticum)***

- Yes
- No

Field type	Radiobutton	Variable name	med_mra
Option group	Yes/No		

If **1.8 Mineralocorticoid receptor antagonist (kaliumsp...** is equal to **No** answer this question:

1.8.1 **Reason no MRA***

- No, due to side-effects
- No, due to allergy
- No, due to contra-indication
- No, due to hyperkalemia
- No, due to no indication
- No, reason unknown
- No, still to be started during uptitration

Field type: Dropdown
Option group: MRA no reason
Variable name: med_mra_no_r

If **1.8 Mineralocorticoid receptor antagonist (kaliumsp...** is equal to **Yes** answer this question:

1.8.2 **Mineralocorticoid antagonist type***

- Eplereonne
- Spironolactone
- Other

Field type: Dropdown
Option group: MRA
Variable name: med_mra_type

If **1.8 Mineralocorticoid receptor antagonist (kaliumsp...** is equal to **Yes** answer this question:

1.8.3 **Mineralocorticoid antagonist daily dosage***
Target dose (mg) Eplerenone 50 o.d. Spironolactone 50 o.d.

[mg]

Field type: Numeric field
Variable name: med_mra_dose

If **1.8 Mineralocorticoid receptor antagonist (kaliumsp...** is equal to **Yes** answer this question:

1.8.4 mineralocorticoid antagonist OMT%

Calculated value

Field type: Calculation
Variable name: med_mra_OMT

If 1.8.4 mineralocorticoid antagonist OMT% is smaller than **100** answer this question:

1.8.4.1 Reason MRA not OMT

- Due to contra-indication
- Due to uptitration phase ongoing
- Due to side-effects
- Due to no indication for heart failure OMT
- Due to maximum tolerable dose achieved
- Unknown

Field type	Dropdown	Variable name	med_mra_OMT_r
Option group	Reason no OMT		

1.9 **Angiotensin receptor-neprilysin inhibitor***

- Yes
- No

Field type	Radiobutton	Variable name	med_ARNI
Option group	Yes/No		

If 1.9 Angiotensin receptor-neprilysin inhibitor is equal to **Yes** answer this question:

1.9.2 **Angiotensin receptor-neprilysin inhibitor dosage***

- 24/26 2dd1
- 49/51 2dd1
- 97/103 2dd1
- Other

Field type	Dropdown	Variable name	med_ARNI_dose
Option group	ARNI dose		

If 1.9 Angiotensin receptor-neprilysin inhibitor is equal to **Yes** answer this question:

1.9.3 Angiotensin receptor-neprilysin inhibitor OMT%

Calculated value

Field type	Calculation	Variable name	med_ARNI_OMT

If **1.9.3 Angiotensin receptor-neprilysin inhibitor OMT%** is smaller than **100** answer this question:

1.9.3.1 Reason ARNI not OMT

- ○ Due to contra-indication
- ○ Due to uptitration phase ongoing
- ○ Due to side-effects
- ○ Due to no indication for heart failure OMT
- ○ Due to maximum tolerable dose achieved
- ○ Unknown

Field type	Dropdown	Variable name	med_ARNI_OMT_r
Option group	Reason no OMT		

1.10 **Ivabradine***

- ○ Yes
- ○ No

Field type	Radiobutton	Variable name	med_ifbl
Option group	Yes/No		

If **1.10 Ivabradine** is equal to **Yes** answer this question:

1.10.1 **Ivabradine daily dosage***
Target dose: 7,5 b.i.d.

[mg]

Field type	Numeric field	Variable name	med_ifbl_dose

If **1.10 Ivabradine** is equal to **Yes** answer this question:

1.10.2 Ivabradine OMT%

[Calculated value]

Field type Calculation Variable name med_ifbl_OMT

If 1.10.2 **Ivabradine OMT%** is smaller than **100** answer this question:

1.10.2.1 Reason ivabradine not OMT

- Due to contra-indication
- Due to uptitration phase ongoing
- Due to side-effects
- Due to no indication for heart failure OMT
- Due to maximum tolerable dose achieved
- Unknown

Field type Dropdown Variable name med_ifbl_OMT_r
Option group Reason no OMT

1.11 **Loop diuretics use***

- None
- Furosemide
- Bumetanide

Field type Dropdown Variable name med_diu_loop
Option group Loop diuretics

If 1.11 **Loop diuretics use** is not equal to **None** answer this question:

1.11.1 **Loop diuretics daily dose (bumetanide/furosemide)***

[ ] mg

Field type Numeric field Variable name med_diu_loop_dose

1.12 **Thiazide diuretics use***

- None
- Thiazide diuretic
- Chloorthalidon

Field type	Dropdown	Variable name	med_diu_thiaz
Option group	Thiazide diuretics		

1.13 **Calcium channel blockers***

- ○ Yes
- ○ No

Field type	Radiobutton	Variable name	med_ccb
Option group	Yes/No		

If **1.13 Calcium channel blockers** is equal to **Yes** answer this question:

1.13.1 **Type of Calcium channnel blockers***

- ○ Dihydropyrine CCB
- ○ Non-dihydropyrine CCB

Field type	Radiobutton	Variable name	med_ccb_type
Option group	ccb_type		

If **1.13 Calcium channel blockers** is equal to **Yes** answer this question:

1.13.2 **Calcium channel blocker daily dose***

[ ] mg

Field type	Numeric field	Variable name	med_ccb_dose

1.14 **SGLT-2 inhibitor***

- ○ Yes
- ○ No

Field type	Radiobutton	Variable name	med_SGLT2i
Option group	Yes/No		

If **1.14 SGLT-2 inhibitor** is equal to **Yes** answer this question:

1.14.1 **SGLT-2 inhibitor***

- ○ Dapagliflozine
- ○ Empagliflozine
- ○ Other

Field type	Radiobutton	Variable name	med_SGLT2i_type
Option group	SGLT2i_type		

If **1.14.1 SGLT-2 inhibitor** is equal to **Other** answer this question:

1.14.1.1 Other SGLT2i

Field type	Textfield	Variable name	med_SGLT2i_type_other

1.15 **Digoxin***

- ○ Yes
- ○ No

Field type	Radiobutton	Variable name	med_dig
Option group	Yes/No		

1.16 **NOAC***

- ○ Yes
- ○ No

Field type	Radiobutton	Variable name	med_noac
Option group	Yes/No		

1.17 **Vitamin K antagonist***

- ○ Yes
- ○ No

Field type	Radiobutton	Variable name	med_vitk
Option group	Yes/No		

1.18 **Platelet aggregation inhibitors***

- ○ Yes
- ○ No

Field type	Radiobutton	Variable name	med_asp
Option group	Yes/No		

1.19 **Statin***

- ○ Yes
- ○ No

Field type	Radiobutton	Variable name	med_sta
Option group	Yes/No		

If **1.19 Statin** is equal to **Yes** answer this question:

1.19.1 **Statin type***

- simvastatine
- rosuvastatin
- atorvastatin
- pravastatin
- fluvastatin
- only ezetimib
- only red yeast rice

Field type: Radiobutton
Option group: Statins
Variable name: med_sta_type

If **1.19.1 Statin type** is not equal to **only red yeast rice** answer this question:

1.19.1.1 **Statin daily dose***

[ ] mg

Field type: Numeric field
Variable name: med_sta_dose

1.20 **Nitrates use***

- Yes
- No

Field type: Radiobutton
Option group: Yes/No
Variable name: med_nitr

1.21 **Amiodarone***

- Yes
- No

Field type: Radiobutton
Option group: Yes/No
Variable name: med_amio

If **1.21 Amiodarone** is equal to **Yes** answer this question:

1.21.1 **Amiodarone daily dose***

mg

Field type Numeric field

Variable name med_amio_dose

1.22 **Alpha blockers (only doxazosine or terazosine)***
only doxazosine/Cardura or terazosine/Hytrin

○ Yes
○ No

Field type Radiobutton
Option group Yes/No

Variable name med_ablock

1.23 **Anti-diabetic medication***

○ Yes
○ No

Field type Radiobutton
Option group Yes/No

Variable name med_antidm

1.24 **Thyroid medication***

○ Yes
○ No

Field type Radiobutton
Option group Yes/No

Variable name med_thyroid

1.25 **Oral iron suppletion***

○ Yes
○ No

Field type: Radiobutton
Option group: Yes/No
Variable name: med_iron_oral

1.26 **Respiratory medication***

- Yes
- No

Field type: Radiobutton
Option group: Yes/No
Variable name: med_resp

1.27 **Colchicine***

- Yes
- No

Field type: Radiobutton
Option group: Yes/No
Variable name: med_colch

Does the patient:

1.29 **Receive immunosuppressive therapy***

- Yes
- No

Field type: Radiobutton
Option group: Yes/No
Variable name: med_imu

If 1.29 Receive immunosuppressive therapy is equal to **Yes** answer this question:

1.29.1 **At which date did the patient start with immunosuppresive therapy***

DD MM YYYY

Error message shown if field's value is greater than **NOW**

No future dates can be entered

Field type Date

Variable name med_imu_d

If 1.29 **Receive immunosuppressive therapy** is equal to **Yes** answer this question:

1.29.2 **Was Inflammatory cardiomyopathy the (main) reason to start with immunosuppresive therapy***

- ○ Yes
- ○ No

Field type Radiobutton
Option group Yes/No

Variable name med_imu_inflcmp

If 1.29 **Receive immunosuppressive therapy** is equal to **Yes** answer this question:

1.29.3 **Which immunosuppresiva did the patient use***

- ☐ Unknown
- ☐ Prednison
- ☐ Azathioprin
- ☐ Ciclosporin
- ☐ Mycofenolate
- ☐ Cyclophoshamide
- ☐ Other

Field type Checkbox
Option group Immunosuppressant Drugs

Variable name med_imu_type

If 1.29.3 **Which immunosuppresiva did the patient use** is checked on **Other** answer this question:

1.29.3.1 Other immunosuppressive drug

Field type Textfield Variable name med_imu_type_other

1.30 **Did the patient receive intravenous iron suppletion?***

- Yes
- No

Field type Radiobutton Variable name med_ferinject
Option group Yes/No

If **1.30 Did the patient receive intravenous iron supple...** is equal to **Yes** answer this question:

1.30.1 **Date intravenous iron suppletion***
If date is not exactly known, use the median date as an estimation (15th of the month if exact day is unknown, or July 1st when only the year is known).

DD MM YYYY

Field type Date Variable name med_ferinject_d

1.31 Other medication
Other medication (type name, dose, unit and frequency. For example: empagliflozine 10mg 1/day) (if available, copy entire medication list here)

Field type Multiline Textfield Variable name Med_other

Please lock this report at the left menu if it has been completed and is filled in correctly

Familiy Member Sudden Cardiac Death

Familiy Member Sudden Cardiac Death

1. Family Member Sudden Cardiac Death

1.1 **Degree family member(s) with sudden cardiac death***

- ○ First degree
- ○ Second degree
- ○ Third degree
- ○ Unknown

Field type: Radiobutton
Option group: Sudden cardiac death family degree
Variable name: FH_SCD_degree

1.2 **Age of family member with sudden cardiac death***

[ ]

Field type: Numeric field
Variable name: FH_SCD_age

1.3 **Cardiomyopathy Phenotype in the family member with SCD?***

- ○ Yes
- ○ No
- ○ Unknown

Field type: Radiobutton
Option group: Yes/No/Unknown
Variable name: FH_SCD_CM_phenotype

1.4 Family member description
e.g. oldest brother, youngest sister, mother, father, uncle on mothers side, etc.

[ ]

Field type	Textfield	Variable name	FH_SCD_description

Holter

Holter

1. Holter

1.1 **Submission method***
semi-automated is applicable when datalists are provided. If uncertain, choose manual.

- ○ manual
- ○ semi-automated

Field type: Radiobutton
Option group: Submission method
Variable name: HOL_sub

1.2 **Holter date performed (aankoppeldatum)***
If date is not exactly known, use the median date as an estimation (15th of the month if exact day is unknown, or July 1st when only the year is known). If completely unknown, use 01-01-1800.

DD MM YYYY

Error message shown if field's value is greater than **NOW**

No future dates can be entered

Field type: Date
Variable name: HOL_d

1.3 **Duration holter registration***

h

Field type: Numeric field
Variable name: HOL_Dur

1.4 **QRS complexes count total***

Field type Numeric field Variable name HOL_QRS

1.5 **Rhythm holter***

- ☐ Sinus rhytm
- ☐ Atrial fibrillation
- ☐ Atrial flutter
- ☐ Pacemaker rhytm
- ☐ Nodal rhytm
- ☐ Other

Field type Checkbox Variable name HOL_rhytm
Option group Rhytm holter

If **1.5 Rhythm holter** is checked on **Pacemaker rhytm** answer this question:

1.5.1 **Rhythm holter***

%

Field type Numeric field Variable name HOL_PM_PER_PACING

If **1.5 Rhythm holter** is checked on **Other** answer this question:

1.5.2 Specify 'other' rhythm

Field type Textfield Variable name HOL_rhytm_other

1.6 **Minimal frequency holter***

bpm

Field type	Numeric field	Variable name	HOL_freq_min

1.7 **Holter frequency average***

bpm

Field type	Numeric field	Variable name	HOL_freq_av

1.8 **Holter max frequency***

bpm

Field type	Numeric field	Variable name	HOL_freq_max

1.9 **Conduction abnormalities***

This checkbox field has too many options to list here. See Option Group - conduction abnormalities for its options.

Field type	Checkbox	Variable name	HOL_geleid
Option group	conduction abnormalities		

1.10 **SVES count***

Field type	Numeric field	Variable name	HOL_SVES

1.11 **SVES percentage***

%

Field type Numeric field Variable name HOL_SVES_per

1.12 **Supra-ventricular tachycardia count***

Field type Numeric field Variable name HOL_SVT_c

If **1.12 Supra-ventricular tachycardia count** is greater than **0** answer this question:

1.12.1 **SVT type***

- ☐ Paroxysmal atrial fibrillation
- ☐ Paroxysmal atrial flutter
- ☐ Paroxysmal atrial tachycardia
- ☐ SVT other

Field type Checkbox Variable name Hol_SVT_type
Option group Supraventricular tachycardia type

If **1.12 Supra-ventricular tachycardia count** is greater than **0** answer this question:

1.12.2 **Longest SVT number of complexes on holter***

Field type Numeric field Variable name Hol_SVT_longest_com

If **1.12 Supra-ventricular tachycardia count** is greater than **0** answer this question:

1.12.3 **Longest SVT in minutes on holter***

min

Field type Numeric field Variable name HOL_SVT_min

If **1.12 Supra-ventricular tachycardia count** is greater than **0** answer this question:

1.12.4 **Longest SVT frequency***

complexes/min

Field type Numeric field Variable name HOL_SVT_max_freq

1.13 **VES holter count***

Field type Numeric field Variable name Hol_VES

If **1.13 VES holter count** is greater than **0** answer this question:

1.13.1 **VES holter percentage***

%

Field type Numeric field Variable name Hol_VES_per

If **1.13 VES holter count** is greater than **0** answer this question:

1.13.2 **VES holter percentage***

- ○ Unifocal
- ○ Multifocal

Field type Dropdown Variable name Hol_VES_focus
Option group Focus VES

If **1.13 VES holter count** is greater than **0** answer this question:

1.13.3 **VES patern***

- ☐ Bigeminy
- ☐ Trigeminy
- ☐ Couplet
- ☐ Triplet

Field type: Checkbox
Option group: VES patern
Variable name: Hol_VES_patern

1.14 **VT count***

Field type: Numeric field
Variable name: HOL_VT_c

If **1.14 VT count** is greater than **0** answer this question:

1.14.1 **VT type***

- ☐ Monomorf
- ☐ Polymorf
- ☐ Non-sustained
- ☐ Sustained
- ☐ VT other

Field type: Checkbox
Option group: VT type
Variable name: HOL_VT_Type

If **1.14 VT count** is greater than **0** answer this question:

1.14.2 **Longest VT number of complexes on holter***

Field type: Numeric field
Variable name: HOL_VT_max_com

If **1.14 VT count** is greater than **0** answer this question:

1.14.3 **Longest VT minutes***

min

Field type: Numeric field
Variable name: HOL_VT_min

If **1.14 VT count** is greater than **0** answer this question:

1.14.4 **Longest VT frequency***

complexes/min

Field type: Numeric field
Variable name: HOL_VT_max_freq

1.15 **Symptoms during holter***

- no symptoms
- no symptoms, though rhytm/conduciton abnormalities
- symptoms, no rhytm/conduciton abnormalities
- during symptoms rhytm/conduciton abnormalities
- during symptoms no rhytm/conduciton abnormalities
- notices PVCs

Field type: Radiobutton
Option group: Symptoms
Variable name: HOL_SYMP

1.16 **Conclusion supraventricular tachycardia holter***

This checkbox field has too many options to list here. See Option Group - Supraventricular tachycardia conclusion for its options.

Field type: Checkbox
Option group: Supraventricular tachycardia conclusion
Variable name: HOL_conc_Supra

1.17 **Conclusion ventricular tachycardia holter***

- ☐ VES, sporadisch
- ☐ VES, frequent
- ☐ VT, monomorf
- ☐ VT, polymorf
- ☐ VT, sustained
- ☐ VT, non-sustained
- ☐ AIVR

Field type	Checkbox	Variable name	HOL_conc_V
Option group	Ventricular tachycardia		

Hospitalization

Hospitalization

1. Hospitalization details

Please ***do not* mention elective hospitalizations (e.g. EFO, elective endo-myocardial biopsies, elective CAG)**!

If date is not exactly known, use the median date as an estimation (15th of the month if exact day is unknown, or July 1st when only the year is known).

If date is not exactly known, use the median date as an estimation (16th of the month if exact day is unknown, or July 2st when only the year is known).

1.3 Date check

Calculated value

Error message shown if field's value is equal to **Error**

Discharge date cannot be before admission date

Field type: Calculation

Variable name: Hosp_d_dif

1.4 **Admitted at cardiology department***

- Yes
- No

Field type: Radiobutton

Option group: Yes/No

Variable name: Hosp_car

If 1.4 **Admitted at cardiology department** is equal to **No** answer this question:

1.4.1 **Cardiology consultation during hospitalisation***

- Yes
- No

Field type Radiobutton
Option group Yes/No
Variable name Hosp_car_icc

1.5 **Reason for hospitalization***
Select all applicable options based on final conclusion of hospitalization, not solely based on initial hypothesis during admission. Life-threatening Ventricular Arrhythmia= Ventricular fibrillation -with or without ICD-shock- **and/or** sustained ventricular tachycardia. Including Out of Hospital Cardiac Arrest.

- ☐ Decompensatio cordis (heart failure)
- ☐ (Suspicion of) acute coronary syndrome
- ☐ Life-threatening Ventricular Arrhythmia (LTA)
- ☐ None Life-threatening Ventricular Arrhytmia (e.g NSVT, VES)
- ☐ Supraventricular arrhytmia
- ☐ Other cardiac
- ☐ Other non-cardiac
- ☐ Unknown

Field type Checkbox
Option group Hospitalization reasons
Variable name Hosp_r

If **1.5 Reason for hospitalization** is checked on **Supraventricular arrhytmia** answer this question:

1.5.1 **Specify supraventricular arrhytmia reason for hospitalization***

- ○ AF/AFL
- ○ Other

Field type Radiobutton
Option group SVT AF/other
Variable name Hosp_r_SVT_type

If **1.5 Reason for hospitalization** is checked on **Other cardiac** answer this question:

1.5.2 **Specify other cardiac reason for hospitalization***

Field type Textfield
Variable name Hosp_r_oth_car

If **1.5 Reason for hospitalization** is checked on **Other non-cardiac** answer this question:

1.5.3 **Specify other non-cardiac reason for hospitalization***

Field type Textfield
Variable name Hosp_r_oth_ncar

1.6 Remarks

Field type Multiline Textfield
Variable name Hosp_rem

Complications during hospitalisation

If 1.5 **Reason for hospitalization** is not checked on **Decompensatio cordis (heart failure)** answer this question:

1.5.4 **Was the patient decompensated during hospitalisation?***
e.g. required further intensification of diuretics or prolongation of hospitalization

- Yes
- No

Field type Radiobutton
Option group Yes/No
Variable name Hosp_inhospital_DC

if multiple LTA occurred during hospitalisation, please mention the first LTA

If **1.7 Did a Life-threatening Arrhythmia occur during...** is equal to **Yes** answer this question:

1.7.1 **Date of Life-threatening Arrhythmia during hospitalisation?***
ventricular fibrillation (with or without ICD-shock), and/or sustained ventricular tachycardia

DD MM YYYY

Error message shown if field's value is greater than **NOW**

date cannot be a future date!

Field type Date

Variable name Hosp_inhospital_LTA_d

1.8 **Detailed information during hospitalization (heart failure specific)***

- Yes
- No

Field type Radiobutton
Option group Yes/No

Variable name Hosp_HF_detail

If 1.8 Detailed information during hospitalization (he... is equal to **Yes** answer this question:

1.8.1 **NYHA class at admission***

- NYHA 1 (No symptoms during ordinary activity)
- NYHA 1-2
- NYHA 2 (Mild symptoms during ordinary activity)
- NYHA 2-3
- NYHA 3 (Marked limitation; comfortable only at rest)
- NYHA 3-4
- NYHA 4 (Symptoms while at rest)
- Unknown

Field type Radiobutton
Option group NYHA

Variable name Hosp_HF_NYHA_adm

If 1.8 Detailed information during hospitalization (he... is equal to **Yes** answer this question:

1.8.2 **NYHA class at discharge**•

- NYHA 1 (No symptoms during ordinary activity)
- NYHA 1-2
- NYHA 2 (Mild symptoms during ordinary activity)
- NYHA 2-3
- NYHA 3 (Marked limitation; comfortable only at rest)
- NYHA 3-4
- NYHA 4 (Symptoms while at rest)
- Unknown

Field type	Radiobutton	Variable name	Hosp_HF_NYHA_dis
Option group	NYHA		

If **1.8 Detailed information during hospitalization (he...** is equal to **Yes** answer this question:

1.8.3 **Weight at admission**•
use 1 decimal

[kg]

Field type	Numeric field	Variable name	Hosp_HF_weight_adm
Limits	30 - 250		

If **1.8 Detailed information during hospitalization (he...** is equal to **Yes** answer this question:

1.8.4 **Weight at discharge**•
use 1 decimal

[kg]

Field type	Numeric field	Variable name	Hosp_HF_weight_dis
Limits	30 - 250		

If **1.8 Detailed information during hospitalization (he...** is equal to **Yes** answer this question:

1.8.5 **Cumulative dose intravenous loop diuretics during hospitalization**•
bumetanide 1mg = furosemide 40mg

[mg furosemide-equivalent]

Field type Numeric field

Variable name Hosp_HF_loop_diu_iv_dose_cum

If **1.8 Detailed information during hospitalization (he...** is equal to **Yes** answer this question:

1.8.6 **Number of days treated with IV loop diuretics during admission***

days

Field type Numeric field

Variable name Hosp_HF_loop_diu_iv_days

If **1.8 Detailed information during hospitalization (he...** is equal to **Yes** answer this question:

1.8.7 **Intravenous vasodilators used during admission***

- Yes
- No
- Unknown

Field type Radiobutton
Option group Yes/No/Unknown

Variable name Hosp_HF_vasodilator

If **1.8 Detailed information during hospitalization (he...** is equal to **Yes** answer this question:

1.8.8 **Inotropes used during admission***

- Yes
- No
- Unknown

Field type Radiobutton
Option group Yes/No/Unknown

Variable name Hosp_HF_inotropes

If **1.8 Detailed information during hospitalization (he...** is equal to **Yes** answer this question:

1.8.9 Thiazide used during admission

- ◯ Not used
- ◯ Already in use
- ◯ Started during admission
- ◯ Unknown

Field type	Radiobutton	Variable name	Hosp_HF_thiazide
Option group	Medication start during admission		

Medical consultations

Medical consultations

1. Consultations

Report data

1.1 **Information changed after Date data cleaned?***

- Yes
- No

Field type: Radiobutton
Option group: Yes/No
Variable name: CP_cleaned_rev

If 1.1 **Information changed after Date data cleaned?** is equal to **Yes** answer this question:

1.1.1 **Variables changed? (report numbers of variables)***

Field type: Textfield
Variable name: CP_cleaned_rev_info

1.2 **Date of consultation***
If date is not exactly known, use the median date as an estimation (15th of the month if exact day is unknown, or July 1st when only the year is known).

DD MM YYYY

Error message shown if field's value is greater than **NOW**

No future dates can be entered

Field type: Date
Variable name: Consult_d

1.3 **Type of consultation***

- Outpatient visit (poli/CP/NP)
- Telephone consultation (TC)
- Inpatient visit (tijdens opname)

Field type: Dropdown
Option group: Consultation type
Variable name: Consult_t

1.4 Consult moment
Consult moment according to NHR guidelines 2021.

Calculated value

Field type: Calculation
Variable name: Consult_moment

Anamnesis

1.5 **NYHA classification***

- NYHA 1 (No symptoms during ordinary activity)
- NYHA 1-2
- NYHA 2 (Mild symptoms during ordinary activity)
- NYHA 2-3
- NYHA 3 (Marked limitation; comfortable only at rest)
- NYHA 3-4
- NYHA 4 (Symptoms while at rest)
- Unknown

Field type: Radiobutton
Option group: NYHA
Variable name: CP_NYHA

If 1.5 NYHA classification is equal to **NYHA 1 (No symptoms during ordinary activity)** answer this question:

1.5.1 **Fatigue***
If unknown use missing.

- Yes
- No

Field type	Radiobutton	Variable name	CP_fatigue
Option group	Yes/No		

1.6 **Chest pain***

- ◯ None
- ◯ Aspecific
- ◯ Atypical angina
- ◯ partially atypical/partially typical angina
- ◯ Typical angina
- ◯ Chest pain, type unknown
- ◯ Unknown

Field type	Dropdown	Variable name	CP_ChP_type
Option group	Chest Pain type		

If **1.7 Chest pain calculation** is equal to **1** answer this question:

1.7.1 CCS classification

- ◯ CCS 0 (mild myocardial ischemia with no symptoms)
- ◯ CCS 1 (angina only with streneous exertion)
- ◯ CCS 2 (angina with moderate exertion)
- ◯ CCS 3 (angina with mild exertion; one stairs at normal pace)
- ◯ CCS 4 (angina at rest)
- ◯ unknown

Field type	Radiobutton	Variable name	CP_ChP_CCS
Option group	CCS		

1.8 **Palpitations***

- ◯ Yes
- ◯ No
- ◯ Unknown

Field type	Radiobutton	Variable name	CP_palp
Option group	Yes/No/Unknown		

1.9 **Dizziness***

- Yes
- No
- Unknown

Field type	Radiobutton	Variable name	CP_dizziness
Option group	Yes/No/Unknown		

If **1.9 Dizziness** is equal to **Yes** answer this question:

1.9.1 **Complaints of orthostasis***

- Yes
- No
- Unknown

Field type	Radiobutton	Variable name	CP_orthostasis
Option group	Yes/No/Unknown		

1.10 **Recent syncope***

- Yes
- No
- Unknown

Field type	Radiobutton	Variable name	CP_sync
Option group	Yes/No/Unknown		

1.11 **Physical activity***

- very poor (ADL dependent)
- poor (ADL independent)
- moderate (1km walking/day)
- fair (>1km walking/day)
- good (exercise 2x/week)
- unknown

Field type	Dropdown	Variable name	CP_physical
Option group	Physical activity		

1.12 Other relevant symptoms

Field type	Multiline Textfield	Variable name	CP_other

If **1.3 Type of consultation** is not equal to **Telephone consultation (TC)**:

Physical examination

If **1.3 Type of consultation** is not equal to **Telephone consultation (TC)** answer this question:

1.3.2 **Height (cm)***

cm

Field type	Numeric field	Variable name	PE_height
Limits	80 - 250		

If **1.3 Type of consultation** is not equal to **Telephone consultation (TC)** answer this question:

1.3.3 **Weight (kg, use 1 decimal)***

kg

Field type	Numeric field	Variable name	PE_weight
Limits	30 - 250		

If **1.3 Type of consultation** is not equal to **Telephone consultation (TC)** answer this question:

1.3.4 Body mass index

Calculated value

Field type	Calculation	Variable name	PE_BMI

If **1.3 Type of consultation** is not equal to **Telephone consultation (TC)** answer this question:

1.3.5 **Signs of cardiac decompensation***
such as pulmonary crackles, peripheral edema or elevated central venous pressure.

- ○ Yes
- ○ No

Field type	Radiobutton	Variable name	PE_CDC
Option group	Yes/No		

If **1.3.5 Signs of cardiac decompensation** is equal to **Yes** answer this question:

1.3.5.1 **Pulmonary crackles***

- ○ no, normal breathing sounds
- ○ yes, pulmonary crackles present
- ○ unknown

Field type	Dropdown	Variable name	PE_Pulm_cr
Option group	Pulmonary crackles		

If **1.3.5 Signs of cardiac decompensation** is equal to **Yes** answer this question:

1.3.5.2 **Peripheral edema***

- no
- yes, ankle level
- yes, tibial level
- yes, knee level
- yes, higher than knee level
- yes, level unknown
- unknown

Field type: Dropdown
Option group: Peripheral edema
Variable name: PE_Ed

If **1.3.5 Signs of cardiac decompensation** is equal to **Yes** answer this question:

1.3.5.3 **Central venous pressure***

- normal
- elevated
- inconclusive/not judgeable
- unknown

Field type: Dropdown
Option group: CVP
Variable name: PE_CVP

If **1.3.5 Signs of cardiac decompensation** is equal to **No** answer this question:

1.3.5.4 **Signs of dehydration***

- Yes
- No
- Unknown

Field type: Radiobutton
Option group: Yes/No/Unknown
Variable name: PE_dehydr

1.13 **Systolic blood pressure***

mmHg

Field type	Numeric field	Variable name	PE_RR_sys
Limits	60 - 250		

1.14 **Diastolic blood pressure***

mmHg

Field type	Numeric field	Variable name	PE_RR_dia
Limits	30 - 150		

Conclusion & plan

1.15 **Conclusion: Heart failure status***

- ◯ Compensated/euvolemia
- ◯ Imminent decompensation
- ◯ Decompensated
- ◯ Dehydrated
- ◯ Unknown

Field type	Dropdown	Variable name	Consult_con_HF_status
Option group	Volume status		

Please lock this report at the left menu if it has been completed and is filled in correctly

Laboratory

Laboratory

1. Laboratory

1.1 **Submission method**

- manual
- semi-automated

Field type	Radiobutton	Variable name	lab_sub
Option group	Submission method		

1.2 **Lab date**

If date is not exactly known, use the median date as an estimation (15th of the month if exact day is unknown, or July 1st when only the year is known).

DD MM YYYY

Error message shown if field's value is greater than **NOW**

No future dates can be entered

Field type	Date	Variable name	lab_d

1.3 **Lab time**

HH MM

Field type	Time	Variable name	lab_time

1.4 **Laboratory analysis location***

- Internal
- External
- Unknown

Field type Radiobutton
Option group Lab Location
Variable name lab_loc

Blood

1.5 serum hemoglobin

mmol/L

Field type Numeric field
Variable name lab_hb

1.6 Serum hematocrit

L/L

Field type Numeric field
Variable name lab_ht

1.7 Serum thrombocytes

10E9/L

Field type Numeric field
Variable name lab_trc

1.8 Serum leukocytes

10E9/L

Field type	Numeric field	Variable name	lab_leu

1.9 Mean corpuscular volume (MCV)

fL

Field type	Numeric field	Variable name	lab_mcv

1.10 Serum sodium

mmol/L

Field type	Numeric field	Variable name	lab_na
Limits	100 - 180		

1.11 Serum potassium

mmol/L

Field type	Numeric field	Variable name	lab_pot
Limits	2 - 8		

1.12 serum ureum/BUN

mmol/L

Field type	Numeric field	Variable name	lab_bun

1.13 Serum creatinine

umol/L

Field type Numeric field Variable name lab_creat

1.14 NT-pro-BNP
Recalculation factors: 1 pg/ml –> 0,1182 pmol/L — 1pmol/L –> 8,457 pmol/L

pmol/L

ⓘ **Warning** message shown if field's value is not equal to **0**

Use unit pmol/L! Use calculator for other units: https://unitslab.com/node/163

Field type Numeric field Variable name lab_ntbnp

1.15 High-sensitive cardiac troponin T (hs-cTnT)

ng/L

Field type Numeric field Variable name lab_hs_ctnt

1.16 **High-sensitive cardiac troponin I (hs-cTnI)***

Field type Numeric field Variable name lab_hs_ctni

1.17 C-reactive protein (CRP)

mg/L

Field type Numeric field Variable name lab_crp

1.18 LDL (indirectly calculcated)

mmol/L

Field type Numeric field Variable name lab_ldl

1.19 TSH

mU/l

Field type Numeric field Variable name lab_tsh

1.20 Free thyroxine (FT4)

pmol/L

Field type Numeric field Variable name lab_ft4

1.21 High-sensitivity C-reactive protein (hs-CRP)

mg/L

Field type Numeric field Variable name lab_hscrp

1.22 Serum ferritin

ug/L

Field type Numeric field Variable name lab_ferrit

1.23 Serum transferrin

g/L

Field type Numeric field Variable name lab_transferrin

1.24 Serum transferritin saturation

%

Field type Numeric field Variable name lab_tsat

1.25 HbA1c (IFCC)

mmol/mol

Field type Numeric field Variable name lab_hba1c_ifcc

1.26 Serum kappa free light chain

mg/L

Field type Numeric field Variable name lab_kappa

1.27 Serum lambda free light chain

mg/L

Field type Numeric field Variable name lab_lambda

1.28 M-proteine serum

- no monoclonal band
- monoclonal band, kappa
- monoclonal band, lambda
- monoclonal band, other
- unknown

Field type	Dropdown	Variable name	lab_m_prot_serum
Option group	M-proteine		

Urine

1.29 Ceatinine in urine (midstream sample)

mmol/L

Field type	Numeric field	Variable name	lab_ur_creat

1.30 Albumin/creatinine in urine (midstream sample)

g/mol creatinine

Warning message shown if field's value is greater than **300**

Is the value above 300 correct? If checked and value really is above 300, proceed.

Field type	Numeric field	Variable name	lab_ur_albcreat

1.31 Urine kappa free light chain (midstream sample)

mg/L

Field type	Numeric field	Variable name	lab_ur_kappa

1.32 Urine lambda free light chain (midstream sample)

mg/L

Field type	Numeric field	Variable name	lab_ur_lambda

1.33 M-proteine urine

- no monoclonal band
- monoclonal band, kappa
- monoclonal band, lambda
- monoclonal band, other
- unknown

Field type	Dropdown	Variable name	lab_ur_m_prot
Option group	M-proteine		

Electrocardiography

Electrocardiography

1. Electrocardiography

1.1 **Submission method***

- manual
- semi-automated

Field type: Radiobutton
Option group: Submission method
Variable name: ecg_sub

1.2 **Date ECG***
If date is not exactly known, use the median date as an estimation (15th of the month if exact day is unknown, or July 1st when only the year is known). If completely unknown, use 01-01-1800.

DD MM YYYY

Error message shown if field's value is greater than **NOW**

No future dates can be entered

Field type: Date
Variable name: ecg_d

1.3 **Rhythm***

- Sinus-/atrial- rhytm
- Atrial fibrillation/flutter
- Nodal Rhytm
- Pacemaker Rhytm
- Ventricular tachycardia
- Other

Field type: Dropdown
Option group: Rhytm
Variable name: ecg_rhytm

If **1.3 Rhythm** is equal to **Other** answer this question:

1.3.1 **Rhythm other***

Field type: Multiline Textfield
Variable name: ecg_rhytm_other

1.4 **Ventricular rate (average)***

bpm

Warning message shown if field's value is smaller or equal than **35**

Are you sure?

Error message shown if field's value is greater or equal than **150**

Are you sure?

Field type: Numeric field
Variable name: ecg_vr

If **1.1 Submission method** is equal to **semi-automated** answer this question:

1.1.1 **P axis***

Field type: Numeric field
Variable name: ecg_axis_p

1.5 **R axis***

Field type Numeric field Variable name ecg_axis_r

If **1.1 Submission method** is equal to **semi-automated** answer this question:

1.1.2 **T axis***

Field type Numeric field Variable name ecg_axis_t

1.6 **PR duration***

ms

Field type Numeric field Variable name ecg_PR

1.7 **QRS duration***

ms

Warning message shown if field's value is smaller or equal than **80**

Are you sure?

Warning message shown if field's value is greater or equal than **200**

Are you sure?

Field type Numeric field Variable name ecg_QRS

1.8 **QT duration***

ms

ⓘ **Warning** message shown if field's value is smaller or equal than **200**

Are you sure?

ⓘ **Warning** message shown if field's value is greater or equal than **600**

Are you sure?

Field type Numeric field Variable name ecg_QT

If 1.1 **Submission method** is equal to **semi-automated** answer this question:

1.1.3 Automatic statements from MUSE

Field type Multiline Textfield Variable name ecg_auto_statements

1.9 Comments

Field type Multiline Textfield Variable name ecg_comments

Blood pressure

1.10 **Pathological Q-waves***

○ Yes
○ No

Field type	Radiobutton	Variable name	ecg_path_qwaves
Option group	Yes/No_		

1.11 **T-wave inversion***

○ Yes
○ No

Field type	Radiobutton	Variable name	ecg_Twave_invers
Option group	Yes/No_		

1.12 **T-top abnormalities***

○ Yes
○ No

Field type	Radiobutton	Variable name	ecg_Ttop_abnorm
Option group	Yes/No_		

1.13 **ST-segment depression***

○ Yes
○ No

Field type	Radiobutton	Variable name	ecg_ST_depr
Option group	Yes/No_		

1.14 **AV Block***

- Yes
- No

Field type	Radiobutton	Variable name	ecg_AV_block
Option group	Yes/No_		

1.15 **Right Bundle Branch Block (RBBB)***

- Yes
- No

Field type	Radiobutton	Variable name	ecg_RBBB
Option group	Yes/No_		

1.16 **Left Bundle Branch Block (LBBB)***

- Yes
- No

Field type	Radiobutton	Variable name	ecg_LBBB
Option group	Yes/No_		

1.17 **Microvoltages***

- Yes
- No

Field type	Radiobutton	Variable name	ecg_micro_V
Option group	Yes/No_		

1.18 **Left Ventricle Hypertrophy***

- ○ Yes
- ○ No

Field type	Radiobutton	Variable name	ecg_LVH
Option group	Yes/No_		

1.19 **Strain***

- ○ Yes
- ○ No

Field type	Radiobutton	Variable name	ecg_strain
Option group	Yes/No_		

1.20 **Pre-excitation***

- ○ Yes
- ○ No

Field type	Radiobutton	Variable name	ecg_pre_excitation
Option group	Yes/No_		

Cardiac implantable electronic devices

Cardiac implantable electronic devices

1. Cardiac implantable electronic devices

1.1 **Type of device***

- ◯ Pacemaker- VVI
- ◯ Pacemaker- DDD
- ◯ CRT-P
- ◯ CRT-D
- ◯ ICD -VVI
- ◯ ICD- DDD
- ◯ ICD
- ◯ Yes, type unknown

Field type: Dropdown
Option group: Device types
Variable name: Device_implant_type

1.2 **Reason for device implantation***

- ◯ primary prevention
- ◯ secondary prevention
- ◯ battery end of life
- ◯ removal of device without new implantation (or device turned off)
- ◯ other

Field type: Dropdown
Option group: reason device
Variable name: Device_implant_reason

1.3 **Date of device implantation***

DD MM YYYY

Error message shown if field's value is greater than **NOW**

No future dates can be entered

Field type: Date
Variable name: Device_implant_d

1.4 **Remarks***

Field type Textfield

Variable name Device_implant_remark

Option Groups

Supraventricular tachycardia conclusion

- SVES Sporadisch
- SVES, Frequent
- Paroxysmaal boezemfibrilleren
- Paroxysmale boezemflutter
- Paroxysmale atriale tachycardie
- SVTs
- Permanent boezemfibrilleren, goede rate-control
- Permanent boezemfibrilleren, trage ventrikelrespons
- Permanent boezemfibrillene, snelle ventrikelrespons
- permanent boezemfibrilleren, zeer uiteenlopende ventrikelrespons
- AV-nodale tachycardie
- CMT

Type cancer

- Lung
- Breast
- Esophageal
- Colo-rectal
- Hematological
- Prostate
- Uterine/ovary
- Melanoma
- Urinary/Bladder
- Thyroid
- Kidney/Renal
- Oropharynx
- Other

type of SVT

- Inappropriate sinustachycardia (IST)
- Sinus nodal reentrant tachycardia (SNRT)
- Ectopic atrial tachycardia
- Multifocal atrial tachycardia (MAT)
- Sinus tachycardia
- Atrioventricular nodal reentrant tachycardia (AVNRT)
- Atrioventricular reentrant tachycardia (AVRT)
- Junctional ectopic tachycardia (JET)
- Nonparoxysmal junctional tachycardia (NPJT)
- Other
- Unknown type

GENE_LIST

- ACTC1
- ACTN2
- ANKRD1
- BAG3
- CALR3
- CAV3
- CRYAB
- CSRP3
- CTNNA3
- DES
- DSC2
- DSG2

- DSP
- EMD
- FHL1
- FLNC
- GLA
- JPH2
- JUP
- LAMA4
- LAMP2
- LDB3
- LMNA
- MIB1
- MYBPC3
- MYH6
- MYH7
- MYL2
- MYL3
- MYOZ2
- MYPN
- NEXN
- PKP2
- PLN
- PRDM16
- PRKAG2
- RBM20
- SCN5A
- TAZ
- TCAP
- TMEM43
- TNNC1
- TNNI3
- TNNT2
- TPM1
- TTN
- TTR
- VCL
- Other

HF etiologie

- Ischemic cardiomyopathy
- Hypertensive cardiomyopathy
- Valvular cardiomyopathy
- Metabolic cardiomyopathy
- Arrhythmia / tachycardia
- Hypertrophic cardiomyopathy
- Idiopathic dilated cardiomyopathy
- Infectious (a.o myocarditis)
- Restrictive cardiomyopathy
- Peripartum cardiomyopathy
- Genetic/Familial
- Pericardial disease
- Toxic cardiomyopathy by alcohol abuses
- Toxic cardiomyopathy by chemotherapy
- Toxic cardiomyopathy by radiation
- Toxic cardiomyopathy by drugs abuses
- Toxic cardiomyopathy by other causes
- Other reasons
- Unknown

conduction abnormalities

- None
- first degree AV-block

- second degree AV-block
- Wenkebach at night
- Wenkebach during the day
- Mobitz II
- Third degree AV-block
- Left bundle branch block
- Right bundle branch block
- Freq. dependent bundle branch block

Rhythm during echo

- sinusrhytm
- atrial fibrillation
- atrial flutter
- AVNR
- Escape rhythm
- AV-pacing
- V-pacing
- Biv-Pacing
- Pacing old Database
- Unknown

auto-immune diseases

- (rheumatoid) arthritis
- Gout
- Bechterew
- Psoriasis
- Crohn/Ulcerative Colitis
- Celiac disease
- Lupus
- Sjogren's syndrome
- Vasculitis
- Other

Beta Blocker Types

- Atenolol
- Bisoprolol
- Carvedilol
- Celiprolol
- Labetalol
- Metoprolol
- Nebivolol
- Pindolol
- Propranolol
- Sotalol
- Other
