# Supplementary material for: The Dutch cardiomyopathy registry (DCR); rationale and registry design
Source: Neth Heart J. 2026 Aug 10;34(9):288–98. doi: 10.1007/s12471-026-02063-5 (PMC13504138; doi:10.1007/s12471-026-02063-5)
Supplement: Supplementary file 2 [file 12471_2026_2063_MOESM2_ESM.docx]

**Supplemental File 2. Governance, Data Sharing, Logstics and Publication Policies**

**Governance**

The registry is coordinated by the NLHI and overseen by the Executive Board, which includes members from the founding partners, Dutch Cardiovascular Alliance (DCVA) and the NLHI. The Executive Board will meet at least every three months, being responsible for overseeing the registry, funding, and costs, potential new partners, research domains and submitted study proposals spanning multiple domains. A majority of >70% is considered enough for voting.

The DCR HCM/DCM Steering Committee is initiated for the HCM/DCM domains, with two participants per founding partner. This Steering committee will meet at least every three months and will discuss data entry, data dictionaries, data quality, submitted study proposals, and data/sample requests. A majority of >70% is considered enough for voting.

**Data Sharing and Logistics**

The central database is hosted by NLHI, with certification for security and data protection, and IT support through the NLHI Durrer Center (https://heart-institute.nl/services/data-biobank/). Involved local researchers will only have restricted access to data from patients known in their center. Only mandated study coordinators have access to the complete database to ensure data quality, prevent duplicates, coordinate family linkage, and handle data distribution after proposal approval. Data access and entry are coordinated in collaboration with local researchers at participating centers. Data issuance is approved by the steering committee (>70% in favor) and individual centers (after steering committee approval each center approves or disapproves the data issuance of its own site), after which mandated study coordinators will process this request to a trusted research environment for the analyses. The local informed consents and national joint data registry agreement will provide the basis for the sharing of data and samples.

Future interested centers will be able to join the DCR by requesting permission through the registry’s Executive Board, officially enrolling in an eligible registry protocol and signing the joint data registry agreement. Abstracts and publications will be discussed within the Governance Structure, to ensure a fair and uniform authorship policy for internal and external DCR projects that lead to publications and/or abstracts. The guiding principle will be that no single site should lead an entire project and ideally the first (junior) and last (senior) authors should be affiliated with different sites.
