# Supplementary material for: The Dutch cardiomyopathy registry (DCR); rationale and registry design
Source: Neth Heart J. 2026 Aug 10;34(9):288–98. doi: 10.1007/s12471-026-02063-5 (PMC13504138; doi:10.1007/s12471-026-02063-5)
Supplement: Supplementary file 3 [file 12471_2026_2063_MOESM3_ESM.docx]

**Supplementary Table 1.** Harmonization of informed consents on the following content

Data storage and usage for research purposes (tiered consent procedure)

- Data sharing (different options)

a. Academic researchers (EU)
b. Academic researchers (non-EU)
c. Commercial companies and other parties

- (virtual) Biobanking (optional)

a. Plasma/Serum
b. Whole blood
c. Peripheral blood mononuclear cells (also basis of iPSC generation)
d. Cardiac and non-cardiac tissue
